# Debiased inference for heterogeneous subpopulations in a high-dimensional logistic regression model

**DOI:** 10.1038/s41598-023-48903-x

**Published:** 2023-12-11

**Authors:** Hyunjin Kim, Eun Ryung Lee, Seyoung Park

**Affiliations:** Department of Statistics, Sungkyunkwan University, Seoul, 100190 South Korea

**Keywords:** Statistics, Cancer genomics, Cancer, Computational biology and bioinformatics, Medical research

## Abstract

Due to the prevalence of complex data, data heterogeneity is often observed in contemporary scientific studies and various applications. Motivated by studies on cancer cell lines, we consider the analysis of heterogeneous subpopulations with binary responses and high-dimensional covariates. In many practical scenarios, it is common to use a single regression model for the entire data set. To do this effectively, it is critical to quantify the heterogeneity of the effect of covariates across subpopulations through appropriate statistical inference. However, the high dimensionality and discrete nature of the data can lead to challenges in inference. Therefore, we propose a novel statistical inference method for a high-dimensional logistic regression model that accounts for heterogeneous subpopulations. Our primary goal is to investigate heterogeneity across subpopulations by testing the equivalence of the effect of a covariate and the significance of the overall effects of a covariate. To achieve overall sparsity of the coefficients and their fusions across subpopulations, we employ a fused group Lasso penalization method. In addition, we develop a statistical inference method that incorporates bias correction of the proposed penalized method. To address computational issues due to the nonlinear log-likelihood and the fused Lasso penalty, we propose a computationally efficient and fast algorithm by adapting the ideas of the proximal gradient method and the alternating direction method of multipliers (ADMM) to our settings. Furthermore, we develop non-asymptotic analyses for the proposed fused group Lasso and prove that the debiased test statistics admit chi-squared approximations even in the presence of high-dimensional variables. In simulations, the proposed test outperforms existing methods. The practical effectiveness of the proposed method is demonstrated by analyzing data from the Cancer Cell Line Encyclopedia (CCLE).

## Introduction

Significant efforts have been made in recent research to perform screening genetic profiling and drug testing in human cancer cell lines to explore how genomic backgrounds influence response to therapy^1^. These efforts have resulted in valuable cancer cell line (CCL) data resources, such as the Cancer Cell Line Encyclopedia (CCLE)^2^. CCLE data provide responses to 24 anticancer drugs in hundreds of cell lines across multiple tumor types, along with genomic information about these cell lines, such as the expression of nearly 20,000 genes. These data are often used to build computational models to predict drug response^3^. For example, to predict drug response using gene expression, researchers have used several methods, including Ridge regression^4^, mixture regression^5^, support vector machine^6^, random forest^7^, and neural networks^8^.

Given that recent large public CCL datasets, such as CCLE, include multiple cancer types, there is a need to develop statistical inference to assess the heterogeneity of the effects of gene expression on a drug across multiple tumor types. Quantifying the heterogeneity of the effects of genes across different tumors can provide valuable information in drug response modeling, as cancer tissue heterogeneity must be considered when modeling drug response across different cancer types^9^. Different tumor types may be characterized by their own tumor-specific genes, and thus tumor type interactions may need to be considered. In addition, it is often observed that patients with different types of cancer have similar expression patterns of certain genes, but the efficacy of treatments varies. For example, HER2 overexpression has been observed in subsets of patients with cancers such as breast and gastric cancer, but a HER2 inhibitor, pertuzumab, is more effective in treating HER2-positive breast cancer compared to HER2-positive gastric cancer^10^. Therefore, interactions with tumor type may need to be considered for more accurate statistical analysis. Several studies have attempted to develop statistical modeling methods for drug response that account for heterogeneity between different cancer types^11,12^. However, a common approach is still to analyze several tumor types simultaneously^2,13^. This approach assumes that there are no interaction effects with tumor-specific factors. Such inconsistencies in the current literature raises a critical research question regarding the uniformity of the effects of gene expression on drug response across different cancer types. To date, this question remains unanswered. This paper aims to fill this gap by developing a statistical test for homogeneity in the effects of gene expression on drug response across multiple cancer types. This task is particularly challenging due to the high-dimensional nature of CCLE data. In a different context, heterogeneity testing between studies in meta-analyses has been investigated^14,15^.

In this study, we focus on statistical inference for the heterogeneity of the effects of gene expression on drug sensitivity across different cancer types representing distinct subpopulations. We consider a binary response setting, where the response for a cell line represents whether that cell line is sensitive or resistant to a drug, because determining whether a patient is sensitive or resistant to anticancer drugs is critical to treatment. More specifically, we focus on statistical inference in a high-dimensional logistic regression model where the population is stratified into heterogeneous subpopulations, i.e., cancer types. We consider the problem of testing whether a given covariate has the same effect on the binary response in different subpopulations in high-dimensional logistic regression settings as follows: for some $j \in \{1,\ldots ,p\}$ and $c \in {\mathbb {R}}$,

1 $$\begin{aligned} {} H_0: \beta ^{(1)}_{j} = \cdots = \beta ^{(G)}_{j} = c \quad \text{ vs } \quad H_1: \text{ not } H_0, \end{aligned}$$

where $\beta ^{(g)}_{j}$ is the underlying regression coefficient of the *j*th covariate for the *g*th subpopulation, and *c* can be specified, e.g. $c=0$, or *c* can be unspecified. The null hypothesis with unspecified *c* indicates homogeneity, i.e., equal effects of the covariate. Testing (1) with unspecified *c* can provide valuable information in pharmacogenomics research, which studies how genes influence response to a drug. In addition, it may be helpful in understanding variations in drug response between cancer types in terms of gene expression. On the other hand, specifying *c*, e.g. $c=0$, suggests that the covariate has zero effect, i.e., it is not significant. Testing (1) with $c=0$ can provide candidates for gene expression markers of drug sensitivity that can be applied to multiple cancer types. Such versatile gene expressions are very useful for research and clinical settings^16^. While testing (1) can provide valuable insights, standard maximum likelihood estimation cannot be used for CCLE data analysis due to the high dimensionality of genes compared to the number of cell lines for tumor types. In recent years, much effort has been devoted to statistical inference for high-dimensional generalized linear models. Several studies have considered inference for either low-dimensional coefficients or a single coefficient in the presence of a large number of nuisance parameters. For example, Van de Geer et al.^17^ studied the theoretical properties of a bias-corrected Lasso^18^ estimator called desparsified Lasso or debiased Lasso. Theoretical properties of different types of bias-corrected estimators have also been studied under high-dimensional linear regression settings^19,20^. In addition, Ning and Liu^21^ proposed a decorrelated score test that can be applied to generic penalized M estimators. Other researchers have considered interval estimation for a single coefficient^22,23^. More recently, Ma et al.^24^ considered the multiple testing problem for high-dimensional logistic regression in two-sample settings.

Despite the nice theoretical properties of these methods in high-dimensional regimes, these methods were developed in classical single-population settings and thus may not be optimal for analyzing data consisting of heterogeneous subpopulations. In addition, the combination of limited sample sizes for cancer types and the high dimensionality of gene expression data poses challenges in obtaining accurate results when testing the homogeneity or significance of a gene’s effect on a drug across different cancer types. Instead of considering inference based on Lasso penalized estimation, a common approach in existing high-dimensional inference, we propose statistical inference based on the fused group Lasso. Our proposed testing procedure consists of two steps. In the first step, we compute a suitable estimator for the underlying coefficients using variable selection and homogeneity detection. Our proposed objective function includes a negative log-likelihood, a group Lasso-type penalty^25^, and a fusion-type penalty^26^. The group Lasso-type penalty controls overall sparsity in the model, i.e., removes irrelevant covariates in all subpopulations, while the fusion-type penalty promotes similarities between coefficients across subpopulations, which can improve estimation efficiency by clustering regression coefficients^27^. Thus, the combination of group Lasso-type and fusion-type penalties allows for sparsity control and integration of samples from different cancer types, effectively addressing the challenges posed by small sample sizes for cancer types. The effectiveness of our tests over Lasso-based approaches is demonstrated by a simulation model in section “Simultation study for an imbalanced design”, where sample sizes for subpopulations are relatively limited. In the second step, we develop a bias correction procedure to correct the bias of our fused group Lasso estimator obtained in the first step. In essence, we extend the de-sparsified Lasso concept of Javanmard and Montanari^19^ to the fused group Lasso method, thereby facilitating statistical inference in high-dimensional regression scenarios. This advancement requires rigorous theoretical investigations of the fused group Lasso technique. However, to the best of our current knowledge, there are limited theoretical investigations of fused group Lasso problems.

Note that Zhou et al.^28^ considered fused sparse group Lasso in a multiple response linear regression model without rigorous convergence and inference analyses. The theoretical analysis of the combined penalty of group Lasso-type and fusion-type is highly non-trivial. In particular, the penalty function is not decomposable with respect to the support of the parameter, which means that the existing theory of decomposable regularizers^29^ cannot be applied^30^. In addition, we provide a computationally efficient algorithm to address the computational difficulties arising from the logistic log-likelihood and the fused Lasso penalty. By integrating the principles of the proximal gradient method and the ADMM into our framework, we effectively handle the computational complexity associated with our settings. Furthermore, our theory and methods can be easily extended to general fused group lasso settings, such as those with discrete responses.

To test the hypothesis (1), existing high-dimensional inference methods based on $\ell _1$ norm penalization, such as the Lasso, could be considered. A naive approach would be to test (1) by applying a separate penalized logistic regression for each subpopulation, using an existing $\ell _1$ norm-based penalization method, such as the debiased Lasso^17^. However, when the sparsity patterns of the underlying coefficients are similar across different subpopulations—as is the case in the CCLE data example—the proposed fused group Lasso approach can combine subpopulations and increase the sample size, resulting in better performance. This was also observed in our simulation analysis and in different contexts^31^.

The rest of the paper is organized as follows. Section “Method and theory” describes the proposed penalization method, its debiased version, and the test statistics with their theoretical properties. Section “Implementation” presents an algorithm that solves the proposed penalization method. Section “Simulation study” examines the finite sample performance of the proposed test along with other competing methods. Section “Application to the CCLE data” illustrates the application of our approach to the Cancer Cell Line Encyclopedia (CCLE) data^2^. Finally, Section “Conclusion” concludes the paper. Additional simulation results are presented in the Supplementary material.

## Method and theory

In this section, we introduce the proposed penalization and test statistics. First, we introduce the notations that will be used throughout the paper. For any positive integer *d*, let $\varvec{I}_d$ be the $d \times d$ identity matrix, and $\varvec{1}_d$ and $\varvec{0}_d$ be the $d \times 1$ vectors of all 1’s and 0’s, respectively. Let ${\varvec{0}_{d_1 \times d_2}}$ and ${\varvec{1}_{d_1 \times d_2}}$ be $d_1\times d_2$ matrices whose entries are all 0’s and 1’s, respectively. When the size of the matrix is obvious, the subscript is sometimes omitted. For an index $1 \le l \le d$, let ${ \varvec{e}_l}$ denote the $p \times 1$ vector with one at the *l*th location and zero everywhere else. For any $d \times 1$ vector ${\varvec{a}}=(a_1,\ldots ,a_d)^\top$, let $\Vert {\varvec{a}}\Vert _q := \left( \sum _{\ell =1}^{d} a^q_\ell \right) ^{1/q}$ for $1 \le q < \infty$ and $\Vert {\varvec{a}}\Vert _{\max } := \max _{1\le \ell \le d}|a_\ell |$. For a set *S*, let |*S*| denote the cardinality of *S*. For a vector ${\varvec{a}}$ and an index set of elements, say *S*, let $\varvec{a}_{S}$ be the $|S|\times 1$ sub-vector of $\varvec{a}$ with elements in *S*. For a matrix ${\varvec{A}}=(A_{ij})_{d_1 \times d_2}$, we let

$$\Vert {\varvec{A}}\Vert _{\max } := \max _{i,j} |A_{ij} |, \quad \Vert {\varvec{A}}\Vert _1 := \sum _{i,j}|A_{ij}| , \quad \Vert {\varvec{A}}\Vert _F := \sqrt{\sum _{i,j}A^2_{ij}}, \quad |\!|\!|\!{\varvec{A}}|\!|\!|\!_1 := \max _j \sum _i |A_{ij}|,$$

and use $\text{ vec }({\varvec{A}})$ to represent vectorization by staking the columns of a matrix ${\varvec{A}}$. For a symmetric matrix ${\varvec{A}}$, let $\lambda _{\min }({\varvec{A}})$ be the minimum eigenvalue of ${\varvec{A}}$. Given a matrix ${\varvec{A}}$ and an index set of rows, say *R*, let ${{\varvec{A}}_{R,\cdot }}$ denote sub-matrix of ${\varvec{A}}$ with rows in *R*. We also use ${\varvec{A}}_{i,\cdot }$ to represent the *i*th row of ${\varvec{A}}$. For any vectors ${\varvec{a}}$ and $\varvec{b}$ of equal length, let $\langle {\varvec{a}}, \varvec{b} \rangle := \sum _i a_i b_i$. For any matrices ${\varvec{A}}$ and ${\varvec{B}}$ with equal dimensions, let $\langle {\varvec{A}},{\varvec{B}}\rangle :=\sum _{i,j}A_{ij}B_{ij}$. For non-negative sequences $\{a_n\}_{n=1}^{\infty }$ and $\{b_n\}_{n=1}^{\infty }$, we write $a_n \ll b_n$ or $a_n = o(b_n)$ if $b_n>0$ and $a_n/b_n \rightarrow 0$. We also write $a_n = O(b_n)$ or $a_n \lesssim b_n$ if $a_n \le C b_n$ for some positive constant *C*. Let $a \vee b$ and $a \wedge b$ denote $\max (a,b)$ and $\min (a,b)$, respectively.

### Setting and problem

Here we present a logistic regression model for heterogeneous subpopulations. Suppose the data come from *G* independent subpopulations, such as tumor sample groups generated independently in CCLE data. For each subpopulation $1 \le g \le G$, there exist $n_g$ pairs $\left\{ \textbf{x}^{(g)}_{i},y^{(g)}_{i}\right\} _{i=1}^{n_g}$, where $y^{(g)}_{i} \in \{0,1\}$ represents a binary response (e.g., binary drug response) of the *i*th subject in the *g*th group (e.g., tumor group) and $\textbf{x}^{(g)}_{i} =\left( x^{(g)}_{i1},\ldots , x^{(g)}_{ip}\right) ^\top \in {\mathbb {R}}^p$ represents a *p*-dimensional vector of covariates, e.g. gene expression variables. We consider the following logistic regression model

2 $$\begin{aligned} y^{(g)}_{i} \mid \textbf{x}^{(g)}_{i} {\sim } \; \text{ Bernoulli }\left( \frac{\exp \left( \left[ \textbf{x}^{(g)}_{i} \right] ^\top {\varvec{\beta }}^{(g)}\right) }{1+\exp \left( \left[ \textbf{x}^{(g)}_{i} \right] ^\top {\varvec{\beta }}^{(g)}\right) }\right) \end{aligned}$$

for each group $g=1,\ldots ,G$ and samples $i=1,\dots ,n_g$, where ${\varvec{\beta }^{(g)}}:=(\beta ^{(g)}_1,\ldots , \beta ^{(g)}_p)^\top$ represents the underlying group-specific coefficient vector for the *g*th group. Let ${\varvec{\beta }}_{(j)}:= \left( \beta ^{(1)}_j,\ldots , \beta ^{(G)}_j \right) ^\top$ denote the underlying coefficient vector for the *j*th covariate. We consider the high-dimensional setting where the number of covariates *p* increases with samples sizes $n_1, \ldots ,n_G$. Let $n = \sum _{g=1}^G n_g$ be the total sample size. We assume that the groups are heterogeneous but share similar characteristics in the sense that most regression covariates have similar effects on the response across different groups. In this study, we consider the case where only a few covariates, e.g., a small number of gene expressions in the CCLE data example, are relevant to the response across different groups, i.e., the index set $S := \{j: \Vert {\varvec{\beta }}_{(j)} \Vert _2 \ne 0 \}$ is sparse in that $s := |S| \ll p$. We also assume that only a few pairs of groups have different covariate effects, i.e., $\Omega :=\left\{ (j,g,g'): \beta ^{(g)}_{j} \ne \beta ^{(g')}_{j}\right\}$ is sparse in that ${\tilde{s}}:=|\Omega | \ll sG^2$.

For a matrix notation, let

$$\begin{aligned} \textbf{y}^{(g)}&= { \left[ y^{(g)}_1,\ldots , y^{(g)}_{n_g} \right] ^\top } \in {\mathbb {R}}^{n_g} \\ \textbf{X}^{(g)}&= \left[ \textbf{x}^{(g)}_1, \ldots , \textbf{x}^{(g)}_{n_g} \right] ^\top \in {\mathbb {R}}^{n_g \times p} \end{aligned}$$

for $1 \le g \le G$, where each column of $\textbf{X}^{(g)}$ has a zero mean and $\ell _2$ norm $\sqrt{n_g}$. Let $\textbf{y}$ be the binary response vector and $\textbf{X}$ be the covariate matrix defined by

$$\begin{aligned} \textbf{y}&= \left[ (\textbf{y}^{(1)})^\top , \ldots , (\textbf{y}^{(G)})^\top \right] \in {\mathbb {R}}^{n} \\ \textbf{X}&= \text{ diag } \left( \textbf{X}^{(1)},\ldots , \textbf{X}^{(G)} \right) \in {\mathbb {R}}^{n \times pG}, \end{aligned}$$

where $\textbf{X}$ is a block-diagonal matrix, consisting of $\textbf{X}^{(g)}$’s. Define the coefficient matrix ${\varvec{B}}$ as ${\varvec{B}}= \left[ {\varvec{\beta }}^{(1)},\ldots , {\varvec{\beta }}^{(G)} \right] \in {\mathbb {R}}^{p \times G}.$ Our main goal is to test the homogeneity of the effects of the *l*th covariate across the *G* groups, i.e., for $1 \le l \le p$,

3 $$\begin{aligned} {} H_0: \beta ^{(1)}_l = \cdots = \beta ^{(G)}_l\quad \text{ vs } \quad H_1: \text{ not } H_0. \end{aligned}$$

Another goal is to test whether the *l*th covariate is significant to at least one of the groups:

4 $$\begin{aligned} {} H_0: \beta ^{(1)}_l = \cdots = \beta ^{(G)}_l =0 \quad \text{ vs } \quad H_1: \text{ not } H_0. \end{aligned}$$

### The penalization method

We propose to test (3) or (4) based on the penalized method using the fused group Lasso. For any $y \in \{0,1\}$ and $\nu \in {\mathbb {R}}$, define $\ell (y,\nu )= -y\nu + \log (1+\exp (\nu ))$. For the loss function $\ell (y,\nu )$, let ${\dot{\ell }}(y,\nu )$ and $\ddot{\ell }(y,\nu )$ denote its first and second derivatives with respect to $\nu$, respectively. For a matrix ${\varvec{\Delta }}= ({\Delta }_{jg})_{1 \le j \le p, 1 \le g \le G} = \left( {\varvec{\Delta }}^{(1)},\ldots , {\varvec{\Delta }}^{(G)}\right) = \left( {\varvec{\Delta }}^\top _{(1)}, \ldots , {\varvec{\Delta }}^\top _{(p)}\right) ^\top$, let

$$\begin{aligned} L_n({\varvec{\Delta }})= \frac{1}{n} \sum _{g=1}^{G} \sum _{i=1}^{n_g}\ell \left( y^{(g)}_i, \left( \textbf{x}^{(g)}_i \right) ^\top {\varvec{\Delta }}^{(g)} \right) . \end{aligned}$$

We propose ${\hat{{\varvec{B}}}}$, which solves the following optimization problem:

5 $$\begin{aligned} {\hat{{\varvec{B}}}}&:= \mathop {\mathrm {arg\,min}}\limits _{{\varvec{\Delta }}\in {\mathbb {R}}^{p \times G}} L_n({\varvec{\Delta }}) + { \lambda _1\sum _{j=1}^{p} w_{j} \Vert {\varvec{\Delta }}_{(j)}\Vert _2 + \lambda _2\sum _{j=1}^{p} \sum _{g < g'} v_{j,gg'} |{\Delta }_{jg} - {\Delta }_{jg'}|}, \end{aligned}$$

where $\lambda _1$ and $\lambda _2$ are non-negative penalty parameters and $w_j$’s and $v_{j,gg'}$’s are non-negative weights.

Let ${{\hat{{\varvec{B}}}}}= \left( {\hat{\beta }}_{jg} \right) _{1 \le j \le p, 1 \le g \le G} = \left( {\hat{{\varvec{\beta }}}}^{(1)},\ldots , {\hat{{\varvec{\beta }}}}^{(G)} \right) = \left( {\hat{{\varvec{\beta }}^\top }}_{(1)}, \cdots , {\hat{{\varvec{\beta }}^\top }}_{(p)} \right) ^\top$. In (5), the two penalty terms are based on the sparsity assumption, as explained in section “Setting and problem”: The group Lasso-type penalty promotes overall sparsity in the total coefficients, i.e., many ${\hat{{\varvec{\beta }}}}_{(j)}$’s are the zero vector; while the fusion-type penalty promotes similarities among ${{\hat{\beta }}}_{jg}$’s for $1 \le g\le G$. For the weights $w_j$ and $v_{j,gg'}$, we consider the following optimization problem:

6 $$\begin{aligned} {\tilde{{\varvec{B}}}}&:=\mathop {\mathrm {arg\,min}}\limits _{{\varvec{\Delta }}\in {\mathbb {R}}^{p \times G}} L_n({\varvec{\Delta }}) + {{\tilde{\lambda }}}_1 \sum _{j=1}^{p}\Vert {\varvec{\Delta }}_{(j)}\Vert _2 + {{\tilde{\lambda }}}_2 \sum _{j=1}^{p} \sum _{g < g'} |{\Delta }_{jg} - {\Delta }_{jg'}|. \end{aligned}$$

Let ${{\tilde{{\varvec{B}}}}}= \left( {{\tilde{\beta }}}_{jg}\right) _{1 \le j \le p, 1 \le g \le G} = \left( {{\tilde{{\varvec{\beta }}}}}^{(1)},\ldots , {{\tilde{{\varvec{\beta }}}}}^{(G)}\right) = \left( {{\tilde{{\varvec{\beta }}}}}^\top _{(1)}, \ldots , {{\tilde{{\varvec{\beta }}}}}^\top _{(p)}\right) ^\top$. Following Zou^32^, we set

$$\begin{aligned} w_{j} = 1/\Vert {{\tilde{{\varvec{\beta }}}}}_{(j)}\Vert _2, \quad v_{j,gg'} = 1/|{\tilde{\beta }}_{jg}-{\tilde{\beta }}_{jg'}|. \end{aligned}$$

The following theorem presents an estimation error bound for the initial estimator ${\tilde{{\varvec{B}}}}$. For the details of the Conditions 1–2 assumed to derive Theorem 1, please refer to section S3.1 of the Supplementary materials.

#### Theorem 1

Assume that Conditions 1–2 in the Supplementary materials hold, and $\max _{1\le g\le G} \Vert {\varvec{\beta }}^{(g)}\Vert _1 \le \tilde{C}$ for some absolute constant ${{\tilde{C}}}>0$. Let the penalty parameters ${{\tilde{\lambda }}}_1$ and ${{\tilde{\lambda }}}_2$ be chosen so that

$$\begin{aligned} {{\tilde{\lambda }}}_1 \ge \sqrt{\frac{16G (\log p + \log G)}{n}}, \quad {{\tilde{\lambda }}}_2 = G^{-3/2} {{\tilde{\lambda }}}_1. \end{aligned}$$

Then, it holds that with probability at least $1- 1/(pG)$,

$$\begin{aligned} \Vert {\tilde{{\varvec{B}}}}-{\varvec{B}}\Vert _F^2 \lesssim {{\tilde{\lambda }}}_1^2 s + {{\tilde{\lambda }}}_2^2 {\tilde{s}}. \end{aligned}$$

Theorem 1 implies that if we take

$$\begin{aligned} {{\tilde{\lambda }}}_1 \asymp \sqrt{\frac{G(\log p + \log G)}{n}}, \quad {{\tilde{\lambda }}}_2 \asymp \sqrt{\frac{\log p +\log G}{nG^2}}, \end{aligned}$$

then the initial estimator ${\tilde{{\varvec{B}}}}$ satisfies

$$\begin{aligned} \Vert {\tilde{{\varvec{B}}}}-{\varvec{B}}\Vert _F^2 = O_p\left( \Big (sG + \frac{{\tilde{s}}}{G^2}\Big ) \frac{\log (p \vee G)}{n}\right) . \end{aligned}$$

The following theorem presents the theoretical properties of the estimator ${\hat{{\varvec{B}}}}$. For the details of the additional Conditions 3–4 required to prove Theorem thm2, please see section S3.1 in the Supplementary materials.

#### Theorem 2

Assume that the conditions of Theorem 1 and Conditions 3–4 in the Supplementary materials hold. Consider a minimizer ${\hat{{\varvec{B}}}}$ with $\lambda _2 = G^{-3/2} \lambda _1$ and

$$\begin{aligned} 8{{\tilde{C}}} \sqrt{\frac{G(\log p + \log G)}{n}} \le \lambda _1 \asymp \sqrt{\frac{G(\log p + \log G)}{n}}. \end{aligned}$$

Define the estimators of *S* and $\Omega$ by

$$\begin{aligned} {\hat{S}}:= \{j:\ \Vert {{\hat{{\varvec{\beta }}}}}_{(j)} \Vert _2 \ne 0 \}, \ {{\hat{\Omega }}}:=\{(j,g,g'): {\hat{\beta }}_{jg} \ne {\hat{\beta }}_{jg'}\}. \end{aligned}$$

Then, $$P({\hat{S}} = S) \rightarrow 1$$ and $$P({{\hat{\Omega }}} = \Omega ) \rightarrow 1$$.

Theorem 2 shows that the proposed fused group Lasso consistently selects relevant covariates and finds the same covariate effects across groups under additional conditions on the correlations between relevant and irrelevant covariates and on the minimum signal strengths. However, if a covariate of interest *j* is not selected in ${\hat{{\varvec{B}}}}$, i.e., $j \notin {\hat{S}}$, statistical inference under the original model is impossible for ${\hat{{\varvec{B}}}}$, while the debiased version of ${\hat{{\varvec{B}}}}$ may give some useful information, e.g., asymptotic distributions and p-values.

### Debiased test

In this subsection, we develop the debiased version of the fused group Lasso estimator ${\hat{{\varvec{B}}}}$ for statistical inference. For $1\le g \le G$, let

$$\begin{aligned} {\varvec{\Sigma }^{(g)}}&:= E\left[ n_g^{-1}\sum _{i=1}^{n_g}\ddot{\ell }\left( y^{(g)}_i, [\textbf{x}^{(g)}_{i}]^\top {\varvec{\beta }}^{(g)}\right) \textbf{x}^{(g)}_{i} [\textbf{x}^{(g)}_{i}]^\top \right] \\ {\varvec{M}^{(g)}}&:= ({\varvec{\Sigma }^{(g)}})^{-1}. \end{aligned}$$

Let $\hat{{\varvec{\Sigma }}}^{(g)}$ be an estimator of ${\varvec{\Sigma }^{(g)}}$ defined by

$$\begin{aligned} \hat{{\varvec{\Sigma }}}^{(g)}:=n_g^{-1}\sum _{i=1}^{n_g}\ddot{\ell }\left( y^{(g)}_i, [\textbf{x}^{(g)}_i]^\top {{\hat{{\varvec{\beta }}}}}^{(g)}\right) \textbf{x}^{(g)}_{i} [\textbf{x}^{(g)}_{i}]^\top . \end{aligned}$$

Using the main idea of Javanmard and Montanari^19^, let ${\hat{\varvec{M}}}^{(g)} := \left[ {\hat{\varvec{m}}}^{(g)}_{1},\ldots , {\hat{ \varvec{m}}}^{(g)}_{p} \right] ^\top \in {\mathbb {R}}^{p \times p}$ be the estimate of ${\varvec{M}^{(g)}}$ defined by solving the quadratic programming

7 $$\begin{aligned} {\hat{\varvec{m}}}^{(g)}_{j}&= \mathop {\mathrm {arg\,min}}\limits _{{\varvec{m}} \in {\mathbb {R}}^{p}} \frac{1}{2} {\varvec{m}}^\top { {\hat{{\varvec{\Sigma }}}}^{(g)} } {\varvec{m}} \quad \text{ subject } \text{ to } { \Vert {\hat{{\varvec{\Sigma }}}}^{(g)} \varvec{m} -\varvec{e}_j\Vert _{\max }} \le \mu _g \end{aligned}$$

for each $1 \le j \le p$ and $1 \le g\le G$, where $\mu _g =C_1 \sqrt{{\log p}/{n_g}}$ for some constant $C_1>0$. We propose a debiased estimator: for $g=1,\dots ,G$,

$$\begin{aligned} {\hat{\varvec{b}}^{(g)}}:={\hat{{\varvec{\beta }}}}^{(g)} - \frac{ {\hat{\varvec{ M}}}^{(g)} }{n_g}\sum _{i=1}^{n_g}{\dot{\ell }}\left( y^{(g)}_{i}, [\textbf{x}^{(g)}_i]^\top {\hat{{\varvec{\beta }}}}^{(g)}\right) \textbf{x}^{(g)}_{i}. \end{aligned}$$

In the debiased estimation approach, Javanmard and Montanari^19^ considered only linear regression, while Van de Geer et al.^17^ examined a generalized linear model. Van de Geer et al.^17^ used nodewise regression^33^ to estimate standard errors for the debiased Lasso estimator. While we use the bias correction technique developed by Javanmard and Montanari^19^, our proposed method differs from existing methods. Specifically, we use the quadratic programming considered in Javanmard and Montanari^19^ to estimate ${\varvec{M}}^{{(g)}}$s instead of approximating the inverse of the sample covariance matrix for covariates. Compared to our method, most existing debiased inference methods for high-dimensional generalized linear models use nodewise regression (e.g. Ma et al.^24^, Tian and Feng^34^, Caner^35^). In addition, most of these existing methods were developed for statistical inference about a parameter in the classical single-population setting, and thus are Lasso to estimate parameters for a single population. In contrast, we develop tests based on our fused group Lasso for inference about parameters for multiple subpopulations.

To derive an asymptotic normality of the debiased Lasso^17^, Van de Geer et al.^17^ assumed that each row of ${\varvec{M}^{(g)}}$ has a small number of non-zero entries. However, this assumption of exact sparsity may not hold in the generalized linear model, as pointed out by Xia et al.^36^. As demonstrated in Javanmard and Montanari^19^, we can achieve asymptotic normality of the proposed debiased estimator without assuming exact sparsity for ${\varvec{M}^{(g)}}$. Theorem 3 presents the theoretical properties of the proposed debiased estimator ${\hat{\varvec{b}}^{(g)}}$. See section S3.2 of the Supplementary materials for the details of Condition 5, which is crucial for proving Theorem thm123.

#### Theorem 3

Suppose conditions of Theorem 2 and Condition 5 in the Supplementary materials hold. Then, the debiased estimator satisfies

$$\begin{aligned} \sqrt{n_g} \left( {\hat{\varvec{b}}^{(g)}}-{\varvec{\beta }}^{(g)}\right) = - \frac{ {\hat{\varvec{ M}}}^{(g)}}{\sqrt{n_g}}\sum _{i=1}^{n_g} {\dot{\ell }}\left( y^{(g)}_{i}, \left[ \textbf{x}^{(g)}_i \right] ^\top {\varvec{\beta }}^{(g)}\right) \textbf{x}^{(g)}_i + {\varvec{\Delta }_g}, \end{aligned}$$

where $$\max _g \Vert {\varvec{\Delta }_g}\Vert _{\max } = o_p(1)$$.

Let ${\hat{\varvec{V}}}^{(g)} :={{\hat{{\varvec{M}}}}^{{(g)}}{\hat{{\varvec{\Sigma }}}}^{{(g)}}} \left[ {\hat{\varvec{M}}}^{(g)}\right] ^\top$ and ${\hat{\varvec{V}}_{(j)}}$ be the $G \times G$ diagonal matrix with diagonal elements $\left\{ \frac{1}{n_g} \hat{V}^{(g)}_{jj}\right\} _{g=1}^{G}$. Let ${\hat{\varvec{b}}_{(j)}}:= \left[ {\hat{b}}^{(1)}_j, \ldots , {\hat{b}}^{(G)}_j \right] ^\top$. Define ${\varvec{S}_j} = \left[ S_{j1},\ldots , S_{jG}\right] ^\top := {\hat{\varvec{V}}_{(j)}}^{-1/2} \left( {\hat{\varvec{b}}_{(j)}}- {\varvec{\beta }}_{(j)}\right)$ and ${\varvec{S}^0_j}:= {\hat{\varvec{V}}^{-1/2}_{(j)}} {\hat{\varvec{b}}_{(j)}}$. For a given significance level $0<\alpha <1$, we reject the null hypothesis $H_0$ in (4) when $\Vert {\varvec{S}^0_j}\Vert _2^2 > \chi ^2_{\alpha }(G)$, where $\chi ^2_{\alpha }(G)$ represents the upper $\alpha$-quantile of a central $\chi ^2$ distribution with *G* degrees of freedom. The notion of weak convergence is not well defined in our setting, since we consider the case where *G* diverges with *n*. Theorem 4 shows that our test for the hypothesis (4) is still valid in the $\chi ^2$ approximation.

#### Theorem 4

Suppose conditions of Theorem 3 hold, and $G^{7/2} = o(\min _{1\le g\le G} n_g)$. Then, we have

$$\begin{aligned} \sup _x |P \left( \Vert {\varvec{S}_j}\Vert _2^2 \le x \right) - P({\chi ^2(G)} \le x)| \rightarrow 0. \end{aligned}$$

The following Corollary implies ${\hat{\varvec{b}}_{(j)}}^\top \hat{V}^{-1}_{(j)}{\hat{\varvec{b}}_{(j)}}\overset{d}{\rightarrow }\ \chi ^2_G$ under the null hypothesis $H_0: \beta ^{(1)}_j = \cdots = \beta ^{(G)}_j =0$ in (4).

#### Corollary 1

Assume Conditions of Theorem 4. Then, under the null hypothesis $H_0$ in (4), for any significance level $0<\alpha < 1$, we have

$$\begin{aligned} \lim _{n \rightarrow \infty } P\big (\Vert {\varvec{S}^0_j}\Vert _2^2 > \chi ^2_{\alpha }(G) \big ) = \alpha . \end{aligned}$$

Based on this result, the corresponding chi-square test statistic for the hypothesis (4) is $\Vert S^0_j\Vert _2^2$, i.e., reject $H_0$ in (4) if $\Vert S^0_j\Vert _2^2 > \chi ^2_{\alpha }(G)$.

Next, we consider testing the homogeneity hypothesis in (3), which can be rewritten as

$$\begin{aligned} H_0: {\varvec{D}}{\varvec{\beta }}_{(j)} = 0 \quad \text{ vs } \quad H_1: \text{ not } H_0, \end{aligned}$$

where ${\varvec{D}}$ represents the $(G-1) \times G$ matrix such that $D_{\ell \ell }=1$ and $D_{\ell , \ell +1}=-1$ for $\ell =1,\ldots ,G-1$. Define

$$\begin{aligned} {\varvec{K}_j}&:={\left( {\varvec{D}}{\hat{{\varvec{V}}}}_{(j)}{\varvec{D}}^\top \right) }^{-1/2} {\varvec{D}}({\hat{\varvec{b}}_{(j)}}-{{\varvec{\beta }}_{(j)}}) \\ {\varvec{K}_j^0}&:={\left( {\varvec{D}}{\hat{\varvec{{\varvec{V}}}}_{(j)}}{\varvec{D}}^\top \right) }^{-1/2} {\varvec{D}}{\hat{\varvec{b}}_{(j)}}. \end{aligned}$$

Theorem 5 shows that the test procedure for the hypothesis (3) based on $\Vert {\varvec{K}_j}\Vert _2^2$ admits a $\chi ^2$ approximation.

#### Theorem 5

Assume Conditions of Theorem 4. Then, we have

$$\begin{aligned} \sup _x \Big |P\big ( \Vert {\varvec{K}_j}\Vert ^2_2 \le x \big ) - P\big ({\chi ^2(G-1)}\le x \big ) \Big | \rightarrow 0. \end{aligned}$$

#### Corollary 2

Assume the conditions of Theorem 4. Then, under the null hypothesis $H_0$ in (3), for any significance level $0<\alpha < 1$, we have

$$\begin{aligned} \lim _{n \rightarrow \infty } P\left( \Vert {\varvec{K}_j^0}\Vert _2^2> \chi ^2_{\alpha }(G-1)\right) =\alpha . \end{aligned}$$

Corollary 2 implies that under $H_0: \beta ^{(1)}_{j} = \cdots =\beta ^{(G)}_{j}$ in (3), it holds that

$$\begin{aligned} {\hat{\varvec{b}}_{(j)}}^\top {\varvec{D}}^\top ({\varvec{D}}{\hat{\varvec{{\varvec{V}}}}_{(j)}}{\varvec{D}}^\top )^{-1}{\varvec{D}}{\hat{\varvec{b}}_{(j)}}\overset{d}{\rightarrow }\ \chi ^2_{G-1}. \end{aligned}$$

Based on this result, the corresponding chi-square test statistic for the hypothesis (3) is $\Vert {\varvec{K}_j^0}\Vert _2^2$, i.e., reject $H_0$ in (3) if $\Vert {\varvec{K}_j^0}\Vert _2^2 > \chi ^2_{\alpha }(G-1)$.

## Implementation

In this section we present the computational algorithm for solving (6). The algorithm for (5) can be obtained in a similar way. We use the proximal gradient method to solve (6). Let ${\tilde{{\varvec{B}}}}^{(t)}$ be the *t*th update in the proximal gradient method. Then the $(t+1)$th update ${\tilde{{\varvec{B}}}}^{(t+1)}$ is given by

8 $$\begin{aligned}&\mathop {\mathrm {arg\,min}}\limits _{{\varvec{\Delta }}\in {\mathbb {R}}^{p \times G}}\frac{1}{n}\ L_n \left( \tilde{\varvec{B}}^{(t)}\right) + \left\langle \nabla L_n({{\tilde{{\varvec{B}}}}}^{(t)}), {\varvec{\Delta }}- \tilde{\varvec{B}}^{(t)} \right\rangle + \frac{\eta }{2} { \Vert {\varvec{\Delta }}-{\tilde{{\varvec{B}}}}^{(t)}\Vert ^2_F} + \tilde{\lambda }_1\sum _{j=1}^{p}\Vert {\varvec{\Delta }}_{j,\cdot }\Vert _2 + \tilde{\lambda }_2\sum _{j=1}^{p}\sum _{g < g'}|{\Delta }_{jg}-{\Delta }_{jg'}|. \end{aligned}$$

We set $\eta =\sum _{g=1}^{G}\Vert \textbf{X}^{(g)}\Vert ^2_F/4n$ based on the convergence properties of the proximal gradient method^37^. Note that $\nabla L_n({\varvec{\Delta }})$ is Lipschitz continuous with Lipschitz parameter $\sum _{g=1}^{G}\Vert \textbf{X}^{(g)}\Vert ^2_F/(4n)$. Let $L_{n,{\varvec{A}}}({\varvec{\Delta }})$ be the first-order approximation of $L_n({\varvec{\Delta }})$ at a matrix ${\varvec{A}}$. Then, (8) can be rewritten as

9 $$\begin{aligned}&\mathop {\mathrm {arg\,min}}\limits _{{\varvec{\Delta }}} L_{n,{\tilde{{\varvec{B}}}}^{(t)}}({\varvec{\Delta }}) + {\frac{\eta }{2}\Vert {\varvec{\Delta }}-{\tilde{{\varvec{B}}}}^{(t)}\Vert ^2_F}+ \tilde{\lambda }_1\sum _{j=1}^{p}\Vert {\varvec{\Delta }}_{j,\cdot }\Vert _2 + {{\tilde{\lambda }}}_2\sum _{j=1}^{p}\sum _{g < g'}|{\Delta }_{jg}-{\Delta }_{jg'}|. \end{aligned}$$

To compute (9), we use the alternating direction method of multipliers (ADMM)^38^. Let ${\varvec{H}}$ be the *G* by $G(G-1)/2$ matrix satisfying

$$\begin{aligned} \sum _{j=1}^{p}\sum _{g < g'}|{\Delta }_{jg}-{\Delta }_{jg'}| = \Vert {\varvec{\Delta }}{\varvec{H}}\Vert _1. \end{aligned}$$

By introducing surrogate variables ${\varvec{A}}$ and ${\varvec{F}}$, (9) can be converted to solving the following optimization problem:

10 $$\begin{aligned} \min _{{\varvec{\Delta }}, {\varvec{A}}, {\varvec{F}}}&\, \, L_{n,{\tilde{{\varvec{B}}}}^{(t)}}({\varvec{\Delta }}) + \frac{\eta }{2}\Vert {\varvec{\Delta }}- {\tilde{{\varvec{B}}}}^{(t)} \Vert ^2_F + {{\tilde{\lambda }}}_1 \sum _{j=1}^{p}\Vert {\varvec{A}}_{j,\cdot }\Vert _2+ {{\tilde{\lambda }}}_2 \Vert {\varvec{F}}\Vert _1 \nonumber \\ {}&\text{ subject } \text{ to } {\varvec{A}}={\varvec{\Delta }} \text{ and } {\varvec{\Delta }}{\varvec{H}}= {\varvec{F}}. \end{aligned}$$

The corresponding augmented Lagrangian is

$$\begin{aligned} K^{(t)}({\varvec{\Delta }}, {\varvec{A}},{\varvec{F}},{\varvec{W}},{\varvec{U}}) :=&L_{n,{\tilde{{\varvec{B}}}}^{(t)}}({\varvec{\Delta }}) + \frac{\eta }{2}\Vert {\varvec{\Delta }}-{\tilde{{\varvec{B}}}}^{(t)}\Vert ^2_F + {{\tilde{\lambda }}}_1 \sum _{j=1}^{p}\left\| {\varvec{A}}_{j,\cdot }\right\| _2 + {{\tilde{\lambda }}}_2 \Vert {\varvec{F}}\Vert _1 \\&+ \langle {\varvec{U}},{\varvec{\Delta }}-{\varvec{A}}\rangle + \langle {\varvec{W}}, {\varvec{\Delta }}{\varvec{H}}- {\varvec{F}}\rangle + \frac{\rho }{2}\Vert {\varvec{\Delta }}-{\varvec{A}}\Vert ^2_F + \frac{\rho }{2}\Vert {\varvec{\Delta }}{\varvec{H}}-{\varvec{F}}\Vert ^2_F, \end{aligned}$$

where ${\varvec{U}}$ and ${\varvec{W}}$ represent dual variables and $\rho >0$ is a fixed parameter. Let ${\tilde{{\varvec{B}}}}^{(t+1,s)}$ be the *s*th update in the ADMM to compute ${\tilde{{\varvec{B}}}}^{(t+1)}$. Then, ${\tilde{{\varvec{B}}}}^{(t+1)}$ for $t=0,1,2,\ldots$ is obtained by iterating the following updates: starting with ${\varvec{A}}^{(0)} = {\varvec{U}}^{(0)} ={\varvec{0}_{p \times G}}$ and ${\varvec{F}}^{(0)} = {\varvec{W}}^{(0)}={\varvec{0}_{p \times \frac{G(G-1)}{2}}}$, we repeat for $s=1,\ldots ,S$,

11 $$\begin{aligned} {\tilde{{\varvec{B}}}}^{(t+1, s)}&=\mathop {\mathrm {arg\,min}}\limits _{{\varvec{\Delta }}}K^{(t)}\left( {\varvec{\Delta }},{{\varvec{A}}}^{(s-1)},{{\varvec{F}}}^{(s-1)},{{\varvec{W}}}^{(s-1)},{{\varvec{U}}}^{(s-1)}\right) \nonumber \\ {{\varvec{A}}}^{(s)}&=\mathop {\mathrm {arg\,min}}\limits _{{\varvec{A}}}K^{(t)} \left( {\tilde{{\varvec{B}}}}^{(t+1,s)},{\varvec{A}},{{\varvec{F}}}^{(s-1)},{{\varvec{W}}}^{(s-1)},{{\varvec{U}}}^{(s-1)} \right) \nonumber \\ {{\varvec{F}}}^{(s)}&=\mathop {\mathrm {arg\,min}}\limits _{{\varvec{F}}} K^{(t)} \left( {\tilde{{\varvec{B}}}}^{(t+1,s)},{{\varvec{A}}}^{(s)},{\varvec{F}},{{\varvec{W}}}^{(s-1)},{{\varvec{U}}}^{(s-1)} \right) \nonumber \\ {{\varvec{W}}}^{(s)}&=\ {{\varvec{W}}}^{(s-1)} + { \rho \left( {\tilde{{\varvec{B}}}}^{(t+1,s)}{\varvec{H}}-{{\varvec{F}}}^{(s)} \right) }, \nonumber \\ {{\varvec{U}}}^{(s)}&=\ {{\varvec{U}}}^{(s-1)} + {\rho \left( {\tilde{{\varvec{B}}}}^{(t+1,s)}-{{\varvec{A}}}^{(s)} \right) }, \end{aligned}$$

and ${\tilde{{\varvec{B}}}}^{(t+1)}$ is updated as ${\tilde{{\varvec{B}}}}^{(t+1)} = {\tilde{{\varvec{B}}}}^{(t+1,S)}$, where the derivations of each update in (11) can be found in section “ADMM update”. We set $S=50$, $\rho =1$, and the maximum iteration number $T=200$ by analysis. The proposed ADMM algorithm is summarized in Algorithm 1. In our simulation and real data examples, it was observed that the algorithm achieves fast convergence. On average, it completes in less than one second, implemented on an Intel Xeon (2.20 GHz). See also section “Computational time comparison”.

**Algorithm 1:**
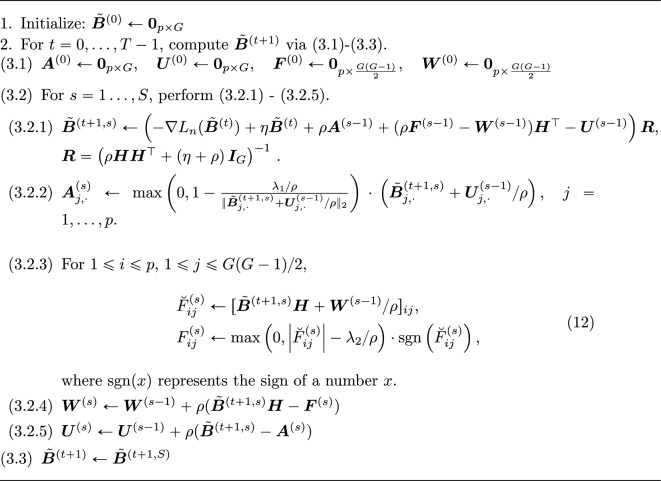
ADMM algorithm for the fused group Lasso logistic regression in (6)

### ADMM update

In this section, we include details of the ADMM update presented at (11).

**Update for **${\tilde{{\varvec{B}}}}^{(t+1,s)}$: For simplicity, let

$$\begin{aligned} K^{(t,s-1)}\left( {\varvec{\Delta }}\right) :=K^{(t)} \left( {\varvec{\Delta }},{\varvec{A}}^{(s-1)}, {\varvec{F}}^{(s-1)}, {\varvec{W}}^{(s-1)}, {\varvec{U}}^{(s-1)}\right) . \end{aligned}$$

By the convexity, ${\tilde{{\varvec{B}}}}^{(t+1,s)}$ must satisfy

$$\begin{aligned} \frac{\partial K^{(t,s-1)}\left( {\varvec{\Delta }}\right) }{\partial {\varvec{\Delta }}} \mid _{{\varvec{\Delta }}={\tilde{{\varvec{B}}}}^{(t+1,s)}} = {\varvec{0}_{p \times G}}. \end{aligned}$$

Thus, it holds that

$$\begin{aligned} {\tilde{{\varvec{B}}}}^{(t+1,s)}\left( \rho {\varvec{H}}{\varvec{H}}^\top + \left( \eta +\rho \right) {\varvec{I}_{G}}\right) = {\varvec{V}}, \end{aligned}$$

where $${\varvec{V}}:= -\nabla L_n \left( \tilde{{\varvec{B}}}^{(t)}\right) +\eta \tilde{{\varvec{B}}}^{(t)} + \rho {\varvec{A}}^{(s-1)} + \rho {\varvec{F}}^{(s-1)} {\varvec{H}}^\top - {\varvec{U}}^{(s-1)} - {\varvec{W}}^{(s-1)} {\varvec{H}}^\top .$$

**Update for **${\varvec{A}}^{(s)}$: ${\varvec{A}}^{(s)}$ is defined by

$$\begin{aligned} {\varvec{A}}^{(s)}&=\mathop {\mathrm {arg\,min}}\limits _{{\varvec{A}}} \ -\langle {\varvec{U}}^{(s-1)}, {\varvec{A}} \rangle + \frac{\rho }{2}\Vert {\tilde{{\varvec{B}}}}^{(t+1,s)}- {\varvec{A}}\Vert ^2_F +{{\tilde{\lambda }}_1}\sum _{j=1}^{p}\Vert {\varvec{A}}_{j,\cdot }\Vert _2, \end{aligned}$$

which is separable with respect to *j*’s. For each $1\le j \le p$, let ${\varvec{s}}_{j}$ be the subgradient of $\Vert {\varvec{x}}\Vert _2$ at ${\varvec{x}}= {\varvec{A}}^{(s)}_{j.}$, i.e.,

$$\begin{aligned} {\varvec{s}}_j={\left\{ \begin{array}{ll} \text{ some } {\varvec{x}} \in {\mathbb {R}}^{1 \times G} \text{ with } \Vert {\varvec{x}}\Vert _2 \le 1 &{} \text{ if } {\varvec{A}}^{(s)}_{j.} = {\varvec{0}^\top _G} \\ {\varvec{A}}^{(s)}_{j,\cdot } /\Vert {\varvec{A}}^{(s)}_{j,\cdot } \Vert _2 &{} \text{ otherwise. } \end{array}\right. } \end{aligned}$$

By the convexity, it holds that for $1 \le j \le p$,

$$\begin{aligned} -\rho \left( {\tilde{{\varvec{B}}}}^{(t+1,s)}_{j,\cdot } - {\varvec{A}}^{(s)}_{j,\cdot }\right) - {\varvec{U}}^{(s-1)}_{j,\cdot } + {{\tilde{\lambda }}_1} {\varvec{s}}_j ={\varvec{0}^\top _{G}}. \end{aligned}$$

By the definition of ${\varvec{s}}_j$, we obtain that for $j=1,\ldots ,p$,

$$\begin{aligned} {\varvec{A}}^{(s)}_{j,\cdot }&=\max \left( 0,1-\frac{{{\tilde{\lambda }}_1}/ \rho }{ \Vert {\tilde{{\varvec{B}}}}^{(t+1,s)}_{j,\cdot } + {\varvec{U}}^{(s-1)}_{j,\cdot }/\rho \Vert _2}\right) \cdot \Bigg (\tilde{ {\varvec{B}}}^{(t+1,s)}_{j,\cdot } + \frac{{\varvec{U}}^{(s-1)}_{j,\cdot }}{\rho } \Bigg ). \end{aligned}$$

**Update for **${\varvec{F}}^{(s)}$: ${\varvec{F}}^{(s)}$ can be derived using the definition of subgradient of $\ell _1$ norm. For each $(i,j) \in \{1,\ldots , p\} \times \left\{ 1,\ldots ,\frac{G(G-1)}{2}\right\}$, it must hold that

$$\begin{aligned} -W^{(s-1)}_{ij} -\rho \left[ {{\tilde{{\varvec{B}}}}}^{(t+1,s)} {\varvec{H}}-{\varvec{F}}^{(s)}\right] _{ij} + {{{\tilde{\lambda }}}_2} \zeta _{ij} = 0, \end{aligned}$$

where $\zeta _{ij}$ is defined by

$$\begin{aligned} \zeta _{ij} = {\left\{ \begin{array}{ll} 1 &{} \text{ if }\quad F^{(s)}_{ij} > 0\\ -1 &{} \text{ if }\quad F^{(s)}_{ij} < 0\\ \text{ some } x \text{ with } |x| \le 1 &{} \text{ if }\quad F^{(s)}_{ij} =0. \end{array}\right. } \end{aligned}$$

By the definition, ${\varvec{F}}^{(s)}=(F^{(s)}_{ij})_{1\le i\le p,\ 1\le j\le \frac{G(G-1)}{2}}$ is given by

$$\begin{aligned} F^{(s)}_{ij} = {\left\{ \begin{array}{ll} \left[ {{\tilde{{\varvec{B}}}}}^{(t+1,s)} {\varvec{H}}\right] _{ij} + \frac{W^{(s-1)}_{ij}}{\rho } - \frac{ {{{\tilde{\lambda }}}_2} }{\rho } &{} \text{ if } {\breve{F}^{(s)}_{ij}} > \frac{ {{{\tilde{\lambda }}}_2} }{\rho } \\ {[}{{\tilde{{\varvec{B}}}}}^{(t+1,s)} {\varvec{H}}]_{ij} + \frac{W^{(s-1)}_{ij}}{\rho } + \frac{{{{\tilde{\lambda }}}_2}}{\rho } &{} \text{ if } {\breve{F}^{(s)}_{ij}} < \frac{-{{{\tilde{\lambda }}}_2}}{\rho }\\ 0 &{} \text{ otherwise }\\ \end{array}\right. } \end{aligned}$$

where $${\breve{F}^{(s)}_{ij}} = \left[ {{\tilde{{\varvec{B}}}}}^{(t+1,s)} {\varvec{H}}\right] _{ij} + \frac{W^{(s-1)}_{ij}}{\rho }$$.

## Simulation study

In this section, we present empirical results through simulation analysis. A main objective of the simulation analysis is to investigate the finite sample performance of the proposed method (Debiased Fused Group Lasso; DFGL) in testing the following hypotheses:

13 $$\begin{aligned} H_0: \beta ^{(1)}_j&= \cdots = \beta ^{(G)}_j = 0\ \text{ vs } \ H_{1}: \text{ not } H_{0}, \end{aligned}$$

14 $$\begin{aligned} H_0: \beta ^{(1)}_j&= \cdots = \beta ^{(G)}_j\ \text{ vs } \ H_{1}: \text{ not } H_{0}. \end{aligned}$$

To investigate the advantages of the proposed tests over existing $\ell _1$ penalized approaches that do not use a fusion penalty, we compared the proposed method with the following methods:

- DL (Debiased Lasso) : Chi-squared test based on applying debiased Lasso^17^ separately for each subpopulation
- DL-B: Bonferroni correction using p-values obtained by applying debiased Lasso^17^ separately for each subpopulation
- DL-E (Debiased Lasso based on the Exact inverse of the information matrix): Chi-squared test based on applying debiased Lasso^36^ separately for each subpopulation, where this bias-correction method is developed for the scenario where the sample size is greater than the number of regressors
- DL-E-B: Bonferroni correction using p-values obtained by applying debiased Lasso^36^ separately for each subpopulation

We also compared the proposed method with DR-B, Bonferroni correction based on debiased Ridge^39^. R code (https://github.com/luxia-bios/DebiasedLassoGLMs) was used to implement DL-E, and R package hdi^40^ was used to implement DL and DR-B. We also used R code (https://web.stanford.edu/~montanar/ssLasso/) to solve the quadratic programming (7). Following Javanmard and Montanari^19^, we set $\mu _g$ in (7) as $\mu _g =c\sqrt{{\log p}/{n_g}}$ for some positive constant *c*. Specifically, we set $c=0.7$ based on the results of the sensitivity analysis of *c* summarized in the Supplementary material. 5-fold cross-validation was used to determine the regularization parameters for the proposed penalization method.

### Simulation setting

In our simulation study, we fix $G=7$ and simulate $\textbf{x}^{(g)}_{i}$ for all (*g*, *i*) from the *p*-dimensional multivariate normal distribution with mean ${{\varvec{0}}_{p}}$ and covariance matrix ${\varvec{\Sigma }_{x}}$. Specifically, we consider the following two different covariance matrices: (1) AR(1): $[{\varvec{\Sigma }_{x}}]_{ij}=0.5^{|i-j|}$; or (2) Block: ${\varvec{\Sigma }_{x}}$ is a $p \times p$ block diagonal matrix consisting of *p*/4 identical blocks, where the $4 \times 4$ sub-block matrix, denoted by ${\varvec{\Sigma }_b}$, is a Toeplitz matrix such that $[{\varvec{\Sigma }_b}]_{ij}=0.5^{|i-j|}$, $i=1,\ldots ,4;~ j=1,\ldots ,4$. Then, the response variables are generated independently as follows: for $1 \le g \le G, \, 1 \le i \le n_g$,

$$\begin{aligned} y^{(g)}_{i} {\sim } \text{ Bernoulli }\left( \frac{\exp ([\textbf{x}^{(g)}_{i}]^\top {\varvec{\beta }}^{(g)})}{ 1+ \exp ([\textbf{x}^{(g)}_{i}]^\top {\varvec{\beta }}^{(g)}) }\right) , \end{aligned}$$

where ${\varvec{\beta }}_{(j)}=\left( \beta ^{(1)}_{j},\ldots , \beta ^{(G)}_{j}\right) ^\top \in {\mathbb {R}}^{G}$ is set as

$$\begin{aligned} {\varvec{\beta }}_{(j)}:= {\left\{ \begin{array}{ll} (-\,0.6, -\,0.6, 0.6, 0.6, 0.6, 0.6, 0.6) &{} j=1 \\ (0.6, 0.6, -\,0.6, -\,0.6, 0.6, 0.6, 0.6) &{} j=2 \\ (0.6, 0.6, 0.6, 0.6, -\,0.4, -\,0.4, 0.6) &{} j=3 \\ (-\,0.4, 0.6, 0.6, 0.6, 0.6, 0.6, -\,0.4) &{} j=4 \\ (0.4, 0.4, 0.4, 0.4, 0.4, 0.4, 0.4) &{} j=5 \\ (0.0, 0.0, 0.0, 0.0, 0.0, 0.0, 0.0) &{} j=6,7 \\ -\,(0.4, 0.4, 0.4, 0.4, 0.4, 0.4, 0.4) &{} j=8 \\ (1.5, 1.5, 1.5, 1.5, 1.5, 1.5, 1.5) &{} j=9,10 \\ (0.0, 0.0, 0.0, 0.0, 0.0, 0.0, 0.0) &{} j=11 \\ (2.5, 2.5, 2.5, 2.5, 2.5, 2.5, 2.5) &{} j=12 \\ (0.0, 0.0, 0.0, 0.0, 0.0, 0.0, 0.0) &{} otherwise\\ \end{array}\right. }. \end{aligned}$$

We set $n_1=\cdots =n_G=m$ and consider the following two specifications of (*m*, *p*): $(m,p)=(200,80)$ or $(m,p)=(300,120)$. For each case, we simulate $M=100$ Monte Carlo samples and summarize the results over 100 replications.

### Simulation results

First, we present simulation results when testing for homogeneity (14). Next, we present simulation results when testing for overall significance (13).

#### Testing homogeneity

We consider $j=1,2,3,4$ and $j=5,8,9,10,12$ to measure powers and type I errors, respectively. Tables 1 and 2 record the powers and type I errors of different methods when the significance level $\alpha$ is set to $\alpha =0.05$. As shown in Table 1, DFGL outperforms the other approaches in terms of higher power. DL ranks second in terms of higher power in most cases, but fails to control Type I errors when the covariates of interest have strong signals. Competing approaches based on a debiased Lasso using the exact inverse of the information matrix also produce type I errors higher than the nominal level when the covariate with the strongest signal is considered. In contrast to the despecified Lasso-based chi-squared test procedures, our proposed DFGL and other Bonferroni-corrected test procedures, including the Ridge-based DR-B, yield type I errors less than or close to the nominal level $\alpha =0.05$, but these Bonferroni-corrected tests are conservative, as shown in Table 1. Note that the Ridge-based approaches are known to be conservative in various contexts^19,41^.

**Table 1:** Power for testing $H_0:\beta ^{(1)}_{j}=\cdots =\beta ^{(G)}_{j}$ vs $H_1:$ not $H_0$ at $\alpha =0.05$, where $n_1,\ldots ,n_G$ are set as $n_1=\cdots =n_G=m$.

(*m*, *p*)	*j*	$\min _{g}\beta ^{(g)}_j$	$\max _{g}\beta ^{(g)}_j$	Methods					
				DFGL	DL	DL-E	DR-B	DL-B	DL-E-B
AR(1)									
(200, 80)	1	$-0.6$	0.6	0.93	0.84	0.54	0.00	0.00	0.00
	2	$-\,0.6$	0.6	0.87	0.76	0.33	0.00	0.00	0.00
	3	$-\,0.4$	0.6	0.62	0.44	0.21	0.00	0.00	0.00
	4	$-\,0.4$	0.6	0.76	0.54	0.22	0.00	0.00	0.00
(300, 120)	1	$-\,0.6$	0.6	1.00	0.97	0.78	0.00	0.00	0.00
	2	$-\,0.6$	0.6	0.97	0.93	0.61	0.00	0.00	0.00
	3	$-\,0.4$	0.6	0.90	0.75	0.44	0.00	0.00	0.00
	4	$-\,0.4$	0.6	0.88	0.87	0.48	0.00	0.00	0.00
Block									
(200, 80)	1	$-\,0.6$	0.6	0.95	0.84	0.57	0.00	0.02	0.01
	2	$-\,0.6$	0.6	0.82	0.60	0.38	0.00	0.00	0.00
	3	$-\,0.4$	0.6	0.66	0.44	0.20	0.00	0.00	0.00
	4	$-\,0.4$	0.6	0.79	0.63	0.31	0.00	0.00	0.00
(300, 120)	1	$-\,0.6$	0.6	1.00	0.97	0.76	0.00	0.00	0.00
	2	$-\,0.6$	0.6	0.99	0.91	0.61	0.00	0.00	0.00
	3	$-\,0.4$	0.6	0.88	0.78	0.40	0.00	0.00	0.00
	4	$-\,0.4$	0.6	0.97	0.91	0.57	0.00	0.01	0.00

**Table 2:** Type I error for testing $H_0:\beta ^{(1)}_{j}=\cdots =\beta ^{(G)}_{j}$ vs $H_1:$ not $H_0$ at $\alpha =0.05$, where $n_1,\ldots ,n_G$ are set as $n_1=\cdots =n_G=m$.

(*m*, *p*)	*j*	$\min _{g}\beta ^{(g)}_j$	$\max _{g}\beta ^{(g)}_j$	Methods					
				DFGL	DL	DL-E	DR-B	DL-B	DL-E-B
AR(1)									
(200, 80)	5	0.4	0.4	0.01	0.02	0.00	0.00	0.00	0.00
	8	$-\,0.4$	$-\,0.4$	0.01	0.03	0.01	0.00	0.00	0.00
	9	1.5	1.5	0.02	0.21	0.05	0.00	0.00	0.00
	10	1.5	1.5	0.05	0.16	0.04	0.00	0.00	0.00
	12	2.5	2.5	0.04	0.35	0.12	0.00	0.00	0.00
(300, 120)	5	0.4	0.4	0.04	0.02	0.02	0.00	0.00	0.00
	8	$-\,0.4$	$-\,0.4$	0.02	0.02	0.00	0.00	0.00	0.00
	9	1.5	1.5	0.03	0.11	0.05	0.00	0.00	0.00
	10	1.5	1.5	0.02	0.09	0.08	0.00	0.00	0.00
	12	2.5	2.5	0.00	0.22	0.14	0.00	0.00	0.00
Block									
(200, 80)	5	0.4	0.4	0.04	0.03	0.02	0.00	0.00	0.00
	8	$-\,0.4$	$-\,0.4$	0.02	0.01	0.00	0.00	0.00	0.00
	9	1.5	1.5	0.05	0.28	0.15	0.00	0.01	0.00
	10	1.5	1.5	0.04	0.15	0.04	0.00	0.00	0.00
	12	2.5	2.5	0.05	0.36	0.21	0.00	0.01	0.01
(300, 120)	5	0.4	0.4	0.01	0.00	0.00	0.00	0.00	0.00
	8	$-\,0.4$	$-\,0.4$	0.03	0.02	0.00	0.00	0.00	0.00
	9	1.5	1.5	0.02	0.26	0.14	0.00	0.00	0.00
	10	1.5	1.5	0.02	0.15	0.07	0.00	0.00	0.00
	12	2.5	2.5	0.03	0.33	0.19	0.00	0.01	0.00

#### Testing significance

In this subsection, we examine the performance of the proposed significance test. We consider $j=1,2,3,4,5,8,9,10,12$ to measure power, while we consider $j=6,7,11,15,20$ to measure type I error. Tables S5 and S6 in the Supplementary materials report the performance of each method in terms of power and Type I error, respectively, when $\alpha =0.05$. The proposed method generally has higher power compared to the competing approaches. In particular, the proposed method is superior to the competing approaches in terms of higher power when the covariates of interest have relatively weak signals. This result was also observed in a previous study^41^, which investigated debiased group Lasso for linear regression. When sample sizes are set as $n_1=\ldots =n_G=300$, DL and the proposed DFGL sometimes provide similar power. However, DL fails to control Type I errors when the covariate of interest is correlated with covariates with strong signals. The proposed DFGL and the other methods, except DL, have type I errors less than or close to the significance level in all cases considered, but the other methods are conservative.

#### Multiple testing

We evaluate the empirical performance of the proposed testing procedures in the context of multiple testing. We consider the following two multiple testing problems, (15) and (16), respectively:

15 $$\begin{aligned} H_{0,j}: \beta ^{(1)}_j&= \ldots = \beta ^{(G)}_j\ {\rm vs } \ H_{1}: {\rm not\, } H_{0,j}, \quad j=1,\ldots ,p \end{aligned}$$

16 $$\begin{aligned} H_{0,j}: \beta ^{(1)}_j&= \cdots = \beta ^{(G)}_j = 0\ {\rm vs } \ H_{1}: { \rm not\, } \,H_{0,j}, \quad j=1,\ldots ,p. \end{aligned}$$

To control the familywise error rate (FWER), we adjust p-values using the Bonferroni-Holm (BH) procedure^42^. We also apply the BH procedure to p-values from DL and those from DL-E. We don’t consider methods using Bonefrroni-correction, i.e., DL-B, DL-E-B, and DR-B, for multiple testing. This is because they are too conservative, as observed in sections “Testing homogeneity” and “Testing significance”.

When considering multiple testing for significance (16), we measure FWER and power as follows:

- FWER: The percentage of the cases where $H_{0,j}$ is rejected for some $j \in S^c$,
- Power: Average of the empirical power $\sum _{j \in {S}} I\left( H_{0,j} \text { is rejected }\right) / s$,

where $S=\{j: H_{0,j} \text{ is } \text{ false }\}$ with cardinality $s=|S|.$ Power and FWER are measured in the same way when considering homogeneity tests. Table 3 summarizes the results. The proposed DFGL has the highest power in all cases, while providing FWER below the nominal level $\alpha =0.05$. Among the competing methods, DL has incorrect control of FWER when considering the homogeneity test (15) and DL-E has poor power, especially when considering the homogeneity test (15).

**Table 3:** Performances of multiple testing at $\alpha =0.05$, where $n_1,\ldots ,n_G$ are set as $n_1=\cdots =n_G=m$.

Testing	Covariate	m	p	Power			FWER		
				DFGL	DL	DL-E	DFGL	DL	DL-E
Homogeneity	AR(1)	200	80	0.408 (0.253)	0.170 (0.197)	0.015 (0.069)	0.000	0.190	0.000
		300	120	0.618 (0.229)	0.358 (0.208)	0.120 (0.172)	0.000	0.060	0.020
	Block	200	80	0.395 (0.291)	0.178 (0.231)	0.045 (0.097)	0.000	0.140	0.000
		300	120	0.690 (0.236)	0.380 (0.245)	0.085 (0.139)	0.010	0.140	0.000
Significance	AR(1)	200	80	0.649 (0.131)	0.518 (0.104)	0.352 (0.048)	0.000	0.030	0.000
		300	120	0.791 (0.092)	0.684 (0.090)	0.428 (0.088)	0.000	0.010	0.000
	Block	200	80	0.690 (0.141)	0.501 (0.110)	0.377 (0.067)	0.010	0.030	0.000
		300	120	0.841 (0.110)	0.677 (0.116)	0.421 (0.085)	0.010	0.020	0.000

### Simultation study for an imbalanced design

In this section, we perform a simulation analysis to assess the performance of DFGL when the sample sizes for the groups vary. Specifically, we consider the following model parameters: $G=3$, $p=300$, $n_1=90$, $n_2=70$, and $n_3=40$, reflecting the dimension of a subset of the CCLE data analyzed in section “Application to the CCLE data”. For each group, we simulate the covariates $\textbf{x}^{{(g)}}_i$ from *p*-dimensional multivariate normal distribution with mean ${{\varvec{0}}_{p}}$ and covariance matrix ${\varvec{\Sigma }_{x}}$ where $[{\varvec{\Sigma }_{x}}]_{ij}=0.5^{|i-j|}$. We set $s=|S|=6$ where $S=\{j:\Vert {\varvec{\beta }}_{(j)}\Vert _2 > 0 \}$ and randomly draw elements of *S* from $\{1,\ldots ,p\}$. As a result, we obtained $S=\{85,129,167,187,211,270\}$, and we set ${\varvec{\beta }}_{(j)}=(\beta ^{(1)}_{j},\cdots , \beta ^{(G)}_{j})^\top \in {\mathbb {R}}^{G}$ as follows:

$$\begin{aligned} {\varvec{\beta }}_{(j)}:= {\left\{ \begin{array}{ll} (1.5,1.5,1.5) &{} j=85 \\ (2.0,2.0,2.0) &{} j=129 \\ (1.0,1.0,-1.0) &{} j=167 \\ (-1.0,1.0,1.0) &{} j=187 \\ (0.8,0.8,-0.8) &{} j=211 \\ (0.8,-0.8,0.8) &{} j=270 \\ (0.0,0.0,0.0) &{} otherwise\\ \end{array}\right. }. \end{aligned}$$

Due to the relatively small sample sizes ($n_1=90$, $n_2=70$, and $n_3=40$), we consider DPL (Debiased Pooled-Lasso) as an additional competing approach. DPL refers to a chi-squared test based on applying bias correction^17^ to Pooled-Lasso, which adapts to Lasso to analyze samples from different groups together, i.e., $\{\textbf{y}, \textbf{X}\}$. Here, $\textbf{y}= \left[ (\textbf{y}^{(1)})^\top , \ldots , (\textbf{y}^{(G)})^\top \right] ^\top$, and $\textbf{X}=\text{ diag } \left( \textbf{X}^{(1)},\ldots , \textbf{X}^{(G)}\right)$ represents a block diagonal matrix consisting of $\textbf{X}^{(1)},\ldots , \textbf{X}^{(G)}$. Note that DL-E can’t be used in this simulation analysis because $p > n_g$ for $g=1,2,3$.

#### Testing homogeneity

We consider $j=167,187,211,270$ and $j=1,2,3,85,129$ to measure powers and type I errors, respectively. Tables 4 and 5 show the powers and type I errors of different methods when $\alpha =0.05$. DFGL outperforms the other methods in terms of higher power. In addition, DFGL successfully controls Type I errors; however, Lasso-based methods, including DL, DPL, and DL-B, produce Type I errors higher than the significance level when testing the null hypothesis $H_0: \beta ^{(1)}_{129}=\beta ^{(2)}_{129}=\beta ^{(3)}_{129}$ where $\beta ^{(1)}_{129}=\beta ^{(2)}_{129}=\beta ^{(3)}_{129}=2$.

**Table 4:** Power for testing $H_0:\beta ^{(1)}_{j}=\cdots =\beta ^{(G)}_{j}$ vs $H_1:$ not $H_0$ at $\alpha =0.05$.

*j*	$\min _{g}\beta ^{(g)}_j$	$\max _{g}\beta ^{(g)}_j$	Methods				
			DFGL	DL	DPL	DR-B	DL-B
167	$-\,1.0$	1.0	0.56	0.32	0.33	0.07	0.30
187	$-\,1.0$	1.0	0.77	0.53	0.54	0.11	0.54
211	$-\,0.8$	0.8	0.26	0.18	0.13	0.00	0.18
270	$-\,0.8$	0.8	0.42	0.24	0.28	0.02	0.22

**Table 5:** Type I error for testing $H_0:\beta ^{(1)}_{j}=\cdots =\beta ^{(G)}_{j}$ vs $H_1:$ not $H_0$ at $\alpha =0.05$.

*j*	$\min _{g}\beta ^{(g)}_j$	$\max _{g}\beta ^{(g)}_j$	Methods				
			DFGL	DL	DPL	DR-B	DL-B
1	0.0	0.0	0.03	0.03	0.04	0.00	0.02
2	0.0	0.0	0.02	0.01	0.00	0.00	0.00
3	0.0	0.0	0.00	0.00	0.00	0.00	0.01
85	1.5	1.5	0.02	0.04	0.02	0.00	0.04
129	2.0	2.0	0.04	0.09	0.09	0.00	0.10

#### Testing signficance

We consider $j=85,129,167,187,211,270$ and $j=1,2,3$ to measure powers and type I errors, respectively. Tables 6 and 7 show the powers and type I errors of different methods when $\alpha =0.05$. While all methods produce type I errors close to or below the significance level, DFGL has higher powers compared to all other methods.

The observed higher power of DFGL in testing for significance and homogeneity compared to Lasso-based approaches is attributed to the fused group Lasso regularization. This regularization method allows for increasing sample sizes in regression parameter estimation by combining subpopulations, leading to more accurate statistical inference. These results suggest the effectiveness of our DFGL in analyzing data characterized by limited sample sizes for groups, such as the CCLE data.

**Table 6:** Power for testing $H_0:\beta ^{(1)}_{j}=\cdots =\beta ^{(G)}_{j}$ vs $H_1:$ not $H_0$ at $\alpha =0.05$.

*j*	$\min _{g}\beta ^{(g)}_j$	$\max _{g}\beta ^{(g)}_j$	Methods				
			DFGL	DL	DPL	DR-B	DL-B
85	1.5	1.5	0.99	0.92	0.91	0.64	0.90
129	2.0	2.0	1.00	1.00	1.00	0.97	1.00
167	$-\,1.0$	1.0	0.67	0.43	0.47	0.14	0.37
187	$-\,1.0$	1.0	0.71	0.47	0.46	0.09	0.42
211	$-\,0.8$	0.8	0.41	0.30	0.23	0.05	0.22
270	$-\,0.8$	0.8	0.39	0.26	0.26	0.04	0.25

**Table 7:** Type I error for testing $H_0:\beta ^{(1)}_{j}=\cdots =\beta ^{(G)}_{j}$ vs $H_1:$ not $H_0$ at $\alpha =0.05$.

*j*	$\min _{g}\beta ^{(g)}_j$	$\max _{g}\beta ^{(g)}_j$	Methods				
			DFGL	DL	DPL	DR-B	DL-B
1	0.0	0.0	0.04	0.04	0.03	0.00	0.02
2	0.0	0.0	0.02	0.01	0.00	0.00	0.00
3	0.0	0.0	0.01	0.02	0.01	0.00	0.03

#### Multiple testing

In this section, we consider the two multiple testing problems (15) and (16), respectively. As in section Multiple testing, we use the BH procedure to control the familywise error rate. In this analysis, we don’t consider DR-B because it is conservative as observed in sections “Testing homogeneity” and “Testing signficance”. Table 8 summarizes the results at the $\alpha =0.05$ significance level. Despite the limited sample size, which results in relatively low power, especially when testing for homogeneity, DFGL outperforms other methods in terms of higher power. All methods provide FWER below the nominal level $\alpha =0.05$.

**Table 8:** Performances of multiple testing at $\alpha =0.05$.

Testing	Power				FWER			
	DFGL	DL	DPL	DL-B	DFGL	DL	DPL	DL-B
Homogeneity	0.120 (0.161)	0.020 (0.068)	0.015 (0.060)	0.025 (0.075)	0.040	0.000	0.000	0.000
Significance	0.408 (0.119)	0.227 (0.096)	0.227 (0.099)	0.133 (0.085)	0.010	0.010	0.000	0.010

### Computational time comparison

In this subsection, we discuss the computational cost of computing the DFGL estimate and compare it with the costs of computing three estimates used in DL, DL-E, and DR-B. To compute Lasso or Ridge penalized estimate, we use R package glmnet. For the penalty parameters, we consider 100 candidates for $(\lambda _1,\lambda _2)$ for DFGL, and 100 candidates for the penalty parameter for DL, DL-E, and DR-B. The penalty parameters are determined using 5-fold Cross-Validation in all methods. Parallel computing is employed to compute estimates for each subpopulation separately in DL and DL-E.

In order to assess computational efficiency, we examine a simulation scenario with the AR(1) covariance matrix for the covariates, $n_1=\cdots =n_G=200$, and $p=80$. Table 9 presents the computational times required to compute four estimates, determined from 100 Monte Carlo simulations and executed on an Intel Xeon (2.20 GHz) processor, are presented in Table 9. A noteworthy observation from Table 9 is that while the DFGL implementation requires slightly more time in comparison to the other methods, it remains significantly expeditious. This can be explained by the fact that DFGL involves addressing a more intricate large-scale problem, entailing a combined penalty of both group Lasso and fusion-type components, whereas the other methods involve simpler Lasso or Ridge techniques entailing a single penalty.

**Table 9:** Average computational times (in seconds) for implementing estimates.

DFGL	DL	DL-E	DR-B
59.58 (0.30)	23.13 (0.63)	1.89 (0.19)	16.98 (1.46)

## Application to the CCLE data

### The dataset

In this section, we present real data analyses when our method is applied to the CCLE data. The CCLE data contains information on cancer treatment responses for 24 drugs on 504 cancer cell lines of 23 cancer types, where the transcription profile of each cell line is characterized by the measured expression levels of 19,177 probes. Cancer cell lines are widely used to understand cancer biology and test the efficacy of novel therapies^43^, and are also used to identify predictive biomarkers for anticancer drug sensitivity^13,44^. We consider three cancer types that include at least 30 cancer cell lines: Lymphoid, Lung, and Skin in our CCLE data analysis. Our main objective is to check whether a specific gene is significant to binary drug response in at least one of these cancer types, and whether such significant genes have heterogeneous effects on a drug across the cancer types. Analyses similar to ours could be useful in two ways. First, examining the significance of the effects of a gene across different cancer types may lead to the identification of potential gene expression markers of drug response that can be used for multiple cancer types. Such versatile gene expression markers are valuable in research^16^. Second, studying the heterogeneity of a gene’s effects across cancer types can provide insights into understanding differences in drug sensitivity across cancer types.

Following Park et al.^45^, we classify cancer cell lines into two categories for each drug. If a drug response value (IC50) is less than 0.5, then the cancer cell line is assigned to the “sensitive” category; otherwise, it is assigned to the “resistant” category. Then, most cancer cell lines are either sensitive or resistant to most drugs in some of the three cancer types. For example, all lymphoid cancer cell lines are resistant to Erlotinib, and only one of the lung cancer cell lines is sensitive to Panobinostat. After removing these imbalanced drugs in our analysis, we consider the following five drugs: 17-AAG, AZD6244, Irinotecan, PD-0325901, and Topotecan. Table 10 presents the number of sensitive cell lines for each of these five drugs.

**Table 10:** The number of sensitive cell lines for each drug across cancer types.

Drug	Cancer type					
	Lung		Lymphoid		Skin	
	Sensitive	Resistance	Sensitive	Resistance	Sensitive	Resistance
17-AAG	20	70	20	48	9	30
AZD6244	84	5	56	12	18	21
Irinotecan	4	46	15	30	12	18
PD-0325901	74	16	52	16	10	29
Topotecan	55	35	8	60	21	18

The analysis of ultra-high-dimensional (UHD) data, such as CCLE data analysis, is accompanied by several challenges, including high collinearity, spurious correlation, noise accumulation, and a significant computational burden. To alleviate these difficulties inherent in UHD data, it is desirable to reduce the dimensionality of the feature space^46,47^. Similar to sure independence screening^46^, we removed relatively irrelevant genes to each drug, respectively, before fitting models. The gene screening procedure is as follows:

1. We selected the top 3,000 genes with the largest sample variances.
2. For $j=1,\ldots , 3000$, we applied logistic regression using each gene and two dummy variables indicating cancer types.
3. We selected the top 300 genes with the smallest p-values for the significance test.

These screening procedures have been used in the literature on high-dimensional regressions (e.g. Park et al.^48^, Wang et al.^49^, Li et al.^45^).

While we analyze a set of $p=300$ genes obtained from the screening procedure described above, the number of genes still exceeds the sample sizes for the cancer types. As a result, standard maximum likelihood estimation couldn’t be used for statistical inference in a model containing the $p=300$ genes. Lasso-based approaches, including debiased Lasso^17^, which do not incorporate a fusion penalty, can be considered to test for heterogeneity or significance of a gene’s effects. However, it is worth noting that approaches based on lasso penalization may yield inaccurate results when applied to data characterized by limited sample sizes, as in the case of our CCLE dataset. This limitation was demonstrated in our simulation study in section “Simultation study for an imbalanced design”. To achieve more accurate statistical inference, we use our DFGL based on a fusion penalty that combines subpopulations.

### Results

**Figure 1:**
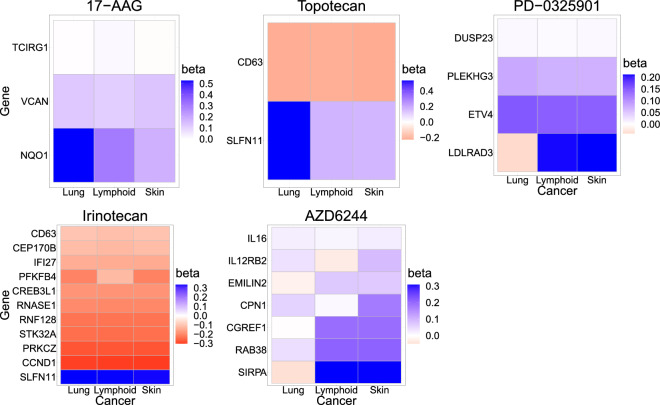
Heatmaps for fused group Lasso estimates ${\hat{\beta }}^{(g)}_{j}$. Genes with estimated regression coefficients of 0’s are omitted when drawing the heatmap. The heatmap was created using the ggplot2 package^50^ (version 3.4.3; https://cran.r-project.org/web/packages/ggplot2/index.html) in R software^51^ (version 4.2.2 for Windows; https://cran.r-project.org/bin/windows/base/old/).

**Figure 2:**
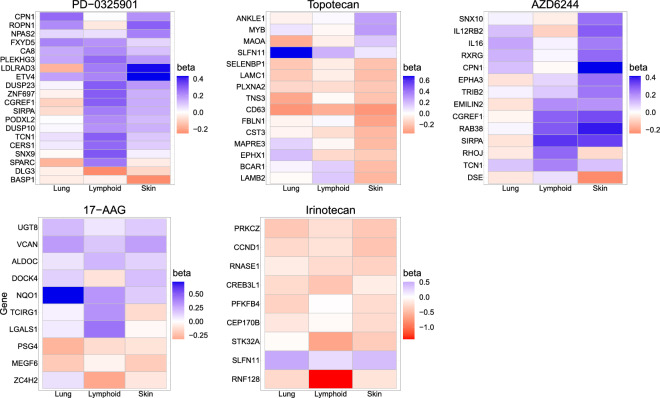
Heatmaps for debiased estimates $\hat{b}^{(g)}_j$. Genes with p-value for the simultaneous significance test greater than 0.05 were omitted when drawing the heatmap. The heatmap was created using the ggplot2 package^50^ (version 3.4.3; https://cran.r-project.org/web/packages/ggplot2/index.html) in R software^51^ (version 4.2.2 for Windows; https://cran.r-project.org/bin/windows/base/old/).

First, we identify important genes using the proposed penalized estimation (5). Figure 1 shows the estimated cancer-specific coefficients for selected genes. We can see that for each drug, the penalized estimates of the coefficients for most genes are similar for at least two of the three cancer types. Next, we apply the proposed simultaneous significance test to investigate whether the genes detected through the penalization have significant effects on the drug when the effects of the other genes are adjusted. Figure 2 shows the debiased estimates corresponding to genes identified by the proposed significance test at the significance level $\alpha =0.05$. Comparing Figs. 1 and 2, we found that most genes selected by the penalization are also significant at the significance level 0.05. However, some genes that were not detected by the penalization were found to be significant for those drugs. Such genes seem to have a significant effect in some of the cancer types or have weak signals across cancer types, as shown in Fig. 2. For example, the absolute value of the estimated effect of BASP1 on PD-0325901 is approximately 0.25 in the skin cancer and less than 0.04 in the other cancer types.

**Figure 3:**
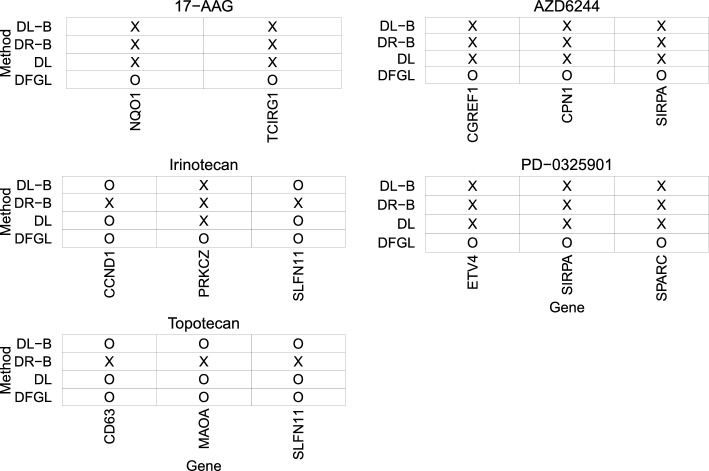
The results of testing significance for some selective genes. The heatmap was created using the ggplot2 package^50^ (version 3.4.4; https://cran.r-project.org/web/packages/ggplot2/index.html) in R software^51^ (version 4.3.1 for Windows; https://cran.r-project.org/bin/windows/base/old/)..

We also use DL, DL-B, and DR-B for comparison with DFGL. In the simulation in section “Simultation study for an imbalanced design”, DPL shows similar performance to DL, so we do not consider DPL in the analysis. Note that DL-E can not be used in this analysis because the number of genes is larger than the sample sizes for the cancer types. Figure 3 compares the results of these methods in terms of testing significance for specific genes. We observe that DR-B seems to be too conservative, as observed in our simulation analysis. However, both the Lasso-based approaches and our method identify some common genes. For example, as shown in Fig. 3, all methods except for DR-B indicate the significance of the effects of SLFN11 on Irinotecan and Topotecan across cancer types. SLFN11 was previously identified as relevant to Irinotecan and Topotecan when a penalized mixture regression was applied to the CCLE data^5^.

When performing a sensitivity analysis based on Bootstrap in section S4.4 in the Supplementary materials, we observe that the following drug-gene pairs are relatively frequently identified by our significance test: SLFN11-Topotecan, SLFN11-Irinotecan, NQO1-17AAG, SIRPA-AZD6244, and ETV4-PD-0325901. These results demonstrate the significance of SLFN11 for Topotecan and Irinotecan. Notably, the remaining three gene-drug pairs were not detected by Lasso-based approaches at $\alpha =0.05$, as shown in Fig. 3. However, NQO1 was identified as an important gene for 17-AAG in the previous analyses of CCLE data^5,52^, and ETV4 was also identified as a related gene to PD-0325901 in the CCLE data analysis performed by Liang et al.^52^. In addition, expression of ETV4, detected as a significant gene expression to PD-0325901, might modulate sensitivity to a MEK inhibitor trametinib^53^. Hayashi et al.^54^ discovered that activation of MEK was induced by ligation of SIRP$\beta$, while SIRP$\alpha$ (SIRPA) is significant to AZD6244. Despite the empirical evidence supporting our significant test results, the gene-drug pairs NQO1-17AAG, ETV4-PD-0325901, and SIPR$\alpha$-AZD6244 were not identified by any of the other significance tests at $\alpha =0.05$. These results suggest that DFGL may provide more accurate results for the significance of the association between a gene and a drug by combining different cancer types, as opposed to approaches based on Lasso or Ridge. Furthermore, as shown in Fig. 2, we expect the associations SIRPA-AZD6244 and ETV4-PD-0325901 to be relatively weak in specific cancer types. The relatively weak effects of SIPRA and ETV4 may result in Lasso-based approaches failing to detect them at the 0.05 significance level. This observation is consistent with our simulation results presented in section “Simultation study for an imbalanced design”, which indicate that Lasso-based approaches have lower power compared to our approach, especially when testing the significance of effects for covariates with relatively weak signals in simulated data with small sample sizes.

**Table 11:** Genes identified by the proposed homogeneity test at $\alpha =0.05$. Genes are listed in order of p-value.

Drug	Target	Gene	# gene
Irinotecan	TOP I	RNF128	1
17-AAG	HSP90	TCIRG1, FUCA2, ZC4H2, LGALS1	4
AZD6244	MEK	CPN1, SIRP$\alpha$, IL12RB2, RAB38, RHOJ, CGREF1	6
PD-0325901	MEK	LDLRAD3, SPARC, CGREF1, CYP27A1, BTBD19, PMP22, SNX9, BAMBI	8
Topotecan	TOP I	SLFN11, MAOA, MAPRE3, BCAR1, EPHX1, RCSD1, FBLN1, LAMB2	8

Our equivalence test shows different results for different cancer types, as summarized in Table 11, i.e., some genes have heterogeneous coefficients depending on the cancer type at the significance level $\alpha =0.05$. The estimated effect of SLFN11 on Topotecan is relatively large in lung cancer cell lines compared to other cancer types. The p-value for the equivalence test corresponding to SLFN11 was less than 0.05 when Topoptecan was considered. However, we note that other methods suggest a lack of significance for the heterogeneity of the effects of SLFN11 on Topotecan at the $\alpha =0.05$ significance level. Further investigation is needed to determine whether SLFN11 has heterogeneous effects on the response to Topotecan in patients with different cancer types. However, in the sensitivity analysis based on Bootstrap in Section S4.4 in the Supplementary materials, the association between SLFN11 and Topotecan appears to be the most significant among all gene-drug pairs in terms of heterogeneous effects across cancer types. In addition, in the previous analysis of CCLE data^5^, a penalized mixture regression suggested that the effects of SLFN11 are different between some of the clusters. These results suggest that our method may uncover underlying heterogeneous effects of a gene across cancer types that are difficult to capture using Lasso or Ridge-based methods. We observed that some genes identified by the homogeneity test have positive estimated coefficients in specific cancer types. For Topotecan, the estimated effect of gene MAOA is only positive in skin cancer cell lines. Low expression of MAOA has been observed in melanoma skin cancer compared with normal samples^55^, but high expression of MAOA has been observed in lung cancer tissues^56^ and lymphoma^57^.

## Conclusion

In this paper, we propose two different tests: (1) testing the homogeneity of the effects of the covariate across different groups and (2) testing the significance of the covariate over groups. We develop non-asymptotic analyses for the proposed fused group Lasso and prove that the debiased test statistics admit chi-squared approximations even in the presence of high dimensional variables. The proposed tests generally outperform the existing bias-correction methods based on Lasso^17,36^ or Ridge^39^ in that it proves higher power, while it controls type I error quite well as shown in section “Simulation study”. Through CCLE data analysis, we can observe that the proposed method can make significant scientific discoveries.

From a methodological point of view, there are some extensions to our method. First, our tests can be applied to generalized linear models, including linear regression and the Poisson regression model, although we focus on logistic regression. In addition, our theoretical analyses can be extended to the generalized linear regression (GLM) setting. Second, we expect that the performance of the proposed tests can be improved by simultaneously estimating the inverse of the information matrices across subpopulations, as in the joint estimation of precision matrices^58,59^.

From the perspective of CCLE data analysis, there are several interesting directions for future research. Although our primary focus in CCLE data analysis was on the use of gene expression, which is known to be predictive of drug response^60^, other omics features such as DNA copy number are available in the analysis of CCL data. It is of great interest to investigate which omics data are most predictive of drug response in a specific cancer type, or have heterogeneous effects on drug response across cancer types. Given the different characteristics of different types of omics data, it is expected that our method may have some limitations in the analysis of multi-omics data. Therefore, a sophisticated extension of our method in estimation and construction of test statistics will be needed for the analysis of multi-omics data. In CCLE data, there are cell lines with missing responses to a drug. Therefore, an extension of our method to include cell lines with missing drug responses would be beneficial for the analysis of CCLE data.

### Supplementary Information

Supplementary Information.

## Data Availability

All data generated or analyzed during this study are included in supplementary information files.
