# Supplementary material for: Debiased inference for heterogeneous subpopulations in a high-dimensional logistic regression model
Source: Sci Rep. 2023 Dec 11;13:21979. doi: 10.1038/s41598-023-48903-x (PMC10713553; doi:10.1038/s41598-023-48903-x)
Supplement: Supplementary file 1 — Supplementary Information. [file 41598_2023_48903_MOESM1_ESM.pdf]

Supplementary materials of 'Debiased inference for heterogeneous subpopulations in a high-dimensional logistic regression model"

Eun Ryung Lee, Hyunjin Kim, and Seyoung Park

Department of Statistics, Sungkyunkwan University, Seoul, Korea

November 22, 2023

In Section S1 and Section S2, we present detailed technical proofs. Section S3 provides the technical conditions assumed to derive technical proofs in Sections S1-S2. In Section S4 and Section S5, we provide additional numerical results.

S1 Proofs

Recall that the row-wise support set is $S = \{j \in \{1, \ldots, p\} : \boldsymbol{\beta}_{(j)} \neq 0\}$ and the indices sets of different coefficient values on the same covariates is $\Omega = \{(j, g, g') : \beta_j^{(g)} \neq \beta_j^{(g')}\}$. Let

$$w_{\min} = \min_{j \in S^c} w_j, \; v_{\min} = \min_{(j,g,g') \in \Omega^c} v_{jgg'}$$
$$\tilde{w}_{\max} = \max_{j \in S} w_j, \; \tilde{v}_{\max} = \max_{(j,g,g') \in \Omega} v_{jgg'}.$$

For a matrix $\boldsymbol{\Delta} \in \mathbb{R}^{p \times G}$, let $\nabla L_n(\boldsymbol{\Delta}) := \nabla L_n(\text{vec}(\boldsymbol{\Delta})) \in \mathbb{R}^{pG}$. For a pG-dimensional vector $\boldsymbol{\delta} = [\boldsymbol{\delta}^{(1)}, \ldots, \boldsymbol{\delta}^{(G)}]^\top$ consisting of p-dimensional vectors $\boldsymbol{\delta}^{(1)}, \ldots, \boldsymbol{\delta}^{(G)}$, let $\text{mat}[\boldsymbol{\delta}] = [\boldsymbol{\delta}^{(1)}, \ldots, \boldsymbol{\delta}^{(G)}] \in \mathbb{R}^{p \times G}$. For a matrix $\boldsymbol{\Delta}$ and index set of columns, say C, let $\boldsymbol{\Delta}_{\cdot,C}$ denote sub-matrix of $\boldsymbol{\Delta}$ with columns in the set C.

Lemma 1. *Let $\boldsymbol{A}$ be a $pG(G-1)/2 \times pG$ matrix consisting of an identity matrix $\boldsymbol{I}_p$ and $-\boldsymbol{I}_p$ satisfying $\boldsymbol{A}^\top \boldsymbol{A} = G \times I_{pG} - \boldsymbol{E} \otimes I_p$, where $\boldsymbol{E} \in \mathbb{R}^{G \times G}$ is a matrix with all ones,*

i.e.,

$$\boldsymbol{A} = \begin{pmatrix} \boldsymbol{I}_p & -\boldsymbol{I}_p & \mathbf{0}_{p\times p} & \cdots & \mathbf{0}_{p\times p} \\ \boldsymbol{I}_p & \mathbf{0}_{p\times p} & -\boldsymbol{I}_p & \cdots & \mathbf{0}_{p\times p} \\ \vdots & \cdots & \cdots & \cdots & \vdots \\ \mathbf{0}_{p\times p} & \cdots & \mathbf{0}_{p\times p} & \boldsymbol{I}_p & -\boldsymbol{I}_p \end{pmatrix}.$$

Suppose that for $1 \leqslant g \leqslant G$ *and* $1 \leqslant j \leqslant p$, $\sum_{i=1}^{n_g}(x_{ij}^{(g)})^2 = n_g$. *It holds that with probability at least* $1 - 1/(pG)$,

$$\max_j \|mat[\nabla L_n(\boldsymbol{B})]_{j,\cdot}\|_2 \leqslant 4\sqrt{G\log(pG)/n}$$
$$\|\boldsymbol{A}\nabla L_n(\boldsymbol{B})\|_{\max} \leqslant 8\sqrt{\log(pG)/n}.$$

Proof. For $1 \leqslant j \leqslant p$ and $1 \leqslant g \leqslant G$, we have

$$\mathrm{mat}[\nabla L_n(\boldsymbol{B})]_{jg} = \sum_{i=1}^{n_g} \frac{1}{n}\epsilon_i^{(g)} x_{ij}^{(g)},$$

where $\epsilon_i^{(g)} := E[y_i^{(g)}] - y_i^{(g)}$. In a matrix form, $\mathrm{mat}[\nabla L_n(\boldsymbol{B})]_{\cdot,g} = \frac{1}{n}[\mathbf{X}^{(g)}]^\top \boldsymbol{E}^{(g)}$, where $\boldsymbol{E}^{(g)} := (\epsilon_1^{(g)}, \ldots, \epsilon_n^{(g)})^\top$. We have that for some $t > 0$,

$$\begin{aligned}
&E[\exp(t\epsilon_i^{(g)} x_{ij}^{(g)}) \mid \mathbf{X}] \\
&= 1 + E\Big[t\epsilon_i^{(g)} x_{ij}^{(g)} + 0.5t^2(\epsilon_i^{(g)})^2(x_{ij}^{(g)})^2 \\
&\qquad + o\left\{t^2(\epsilon_i^{(g)})^2(x_{ij}^{(g)})^2\right\} \mid \mathbf{X}\Big] \\
&= 1 + 0.5t^2(x_{ij}^{(g)})^2 E[(\epsilon_i^{(g)})^2] \\
&\qquad + E\left[o\left\{t^2(\epsilon_i^{(g)})^2(x_{ij}^{(g)})^2\right\} \mid \mathbf{X}\right] \\
&\leqslant 1 + t^2(x_{ij}^{(g)})^2 + o\left(t^2(x_{ij}^{(g)})^2\right) \\
&\leqslant \exp\left(2t^2(x_{ij}^{(g)})^2\right),
\end{aligned}$$

where the second equation holds due to a Taylor series expansion, the first inequality follows from the fact that $(\epsilon_i^{(g)})^2 \leqslant 1$, and the second inequality follows from the fact that $1 + x \leqslant \exp(x)$ for any x. Thus, $\epsilon_i^{(g)} x_{ij}^{(g)}$ is sub-Gaussian with variance proxy $4(x_{ij}^{(g)})^2$. Hence, by the Hoeffding bound (Proposition 2.5 of Wainwright [1]), we obtain

$$\begin{aligned}
P\left[\left|\frac{1}{n}\sum_{i=1}^{n_g}\epsilon_i^{(g)} x_{ij}^{(g)}\right| \geqslant \delta \mid X\right] &\leqslant \exp\left(-\frac{n^2\delta^2}{8\sum_{i=1}^{n_g}(x_{ij}^{(g)})^2}\right) \\
&= \exp\left(-\frac{n^2\delta^2}{8n_g}\right).
\end{aligned}$$

By the uniform bound, we obtain

$$P\left[\max_{1\leqslant j\leqslant p, 1\leqslant g\leqslant G}|\mathrm{mat}[\nabla L_n(\boldsymbol{B})]_{jg}| \geqslant \delta \mid \mathbf{X}\right]$$
$$\leqslant \sum_{g=1}^{G} p \ \exp\left(-\frac{n^2\delta^2}{8n_g}\right)$$
$$\leqslant \exp\left(\log(pG) - \frac{n\delta^2}{8}\right),$$

where the last inequality follows from $n_g \leqslant n$. Hence, with probability at least $1-1/(pG)$, we have

$$\|\nabla L_n(\boldsymbol{B})\|_{\max} := \max_{j,g}|\mathrm{mat}[\nabla L_n(\boldsymbol{B})]_{jg}|$$
$$\leqslant \sqrt{\frac{16\log(pG)}{n}},$$

which implies

$$\max_j \|[\nabla L_n(\boldsymbol{B})]_{j,\cdot}\|_2 \leqslant \sqrt{\frac{16G\log(pG)}{n}}.$$

Because each row of $\boldsymbol{A}$ has only one 1 and one -1, respectively, we have $\|\boldsymbol{A}\nabla L_n(\boldsymbol{B})\|_{\max} \leqslant 2\|\nabla L_n(\boldsymbol{B})\|_{\max} \leqslant 8\sqrt{\log(pG)/n}$. This completes the proof. □

Proof of Theorem 1

Define

$$g(\boldsymbol{\Delta}, \tilde{\lambda}_1, \tilde{\lambda}_2) := \tilde{\lambda}_1\sum_{j=1}^{p}\|\boldsymbol{\Delta}_{(j)}\|_2 + \tilde{\lambda}_2\sum_{j=1}^{p}\sum_{g<g'}|\Delta_{jg} - \Delta_{jg'}|$$

Recall that the initial estimator $\tilde{\boldsymbol{B}}$ is defined by

$$\tilde{\boldsymbol{B}} := \operatorname*{arg\,min}_{\boldsymbol{\Delta}\in\mathbb{R}^{p\times G}} \frac{1}{n}\sum_{g=1}^{G}\sum_{i=1}^{n_g}\ell(y_i^{(g)}, (\mathbf{x}_i^{(g)})^\top\boldsymbol{\Delta}^{(g)}) + g(\boldsymbol{\Delta}, \tilde{\lambda}_1, \tilde{\lambda}_2).$$

Hence, we have

$$\frac{1}{n}\sum_{g=1}^{G}\sum_{i=1}^{n_g}\ell(y_i^{(g)}, (\mathbf{x}_i^{(g)})^\top\tilde{\boldsymbol{\beta}}^{(g)}) + g(\tilde{\boldsymbol{B}}, \tilde{\lambda}_1, \tilde{\lambda}_2)$$
$$\leqslant \frac{1}{n}\sum_{g=1}^{G}\sum_{i=1}^{n_g}\ell(y_i^{(g)}, (\mathbf{x}_i^{(g)})^\top\boldsymbol{\beta}^{(g)}) + g(\boldsymbol{B}, \tilde{\lambda}_1, \tilde{\lambda}_2).$$

For $1 \leqslant g \leqslant G$, denote

$$\ell_g(\boldsymbol{\beta}^{(g)}) := \frac{1}{n}\sum_{i=1}^{n_g} \ell(y_i^{(g)}, (\mathbf{x}_i^{(g)})^\top \boldsymbol{\beta}^{(g)}).$$

Let $\boldsymbol{\delta}^{(g)} := \tilde{\boldsymbol{\beta}}^{(g)} - \boldsymbol{\beta}^{(g)} \in \mathbb{R}^p$. By Taylor's theorem, we have with $\ddot{\boldsymbol{\beta}}^{(g)} = u\tilde{\boldsymbol{\beta}}^{(g)} + (1-u)\boldsymbol{\beta}^{(g)}$ for some $u \in (0,1)$,

$$\begin{aligned}
&\ell_g(\boldsymbol{\beta}^{(g)} + \boldsymbol{\delta}^{(g)}) - \ell_g(\boldsymbol{\beta}^{(g)}) - \langle \nabla \ell_g(\boldsymbol{\beta}^{(g)}), \boldsymbol{\delta}^{(g)} \rangle \\
&= (\boldsymbol{\delta}^{(g)})^\top \left\{ \frac{1}{n}\sum_{i=1}^{n_g} \frac{\exp\left((\mathbf{x}_i^{(g)})^\top \ddot{\boldsymbol{\beta}}^{(g)}\right)}{\left(1+\exp((\mathbf{x}_i^{(g)})^\top \ddot{\boldsymbol{\beta}}^{(g)})\right)^2} \mathbf{x}_i^{(g)} (\mathbf{x}_i^{(g)})^\top \right\} \boldsymbol{\delta}^{(g)} \\
&\geqslant (\boldsymbol{\delta}^{(g)})^\top \left\{ \frac{1}{n}\sum_{i=1}^{n_g} \frac{\exp(-2C\tilde{C})}{\left(1+\exp(-2C\tilde{C})\right)^2} \mathbf{x}_i^{(g)} (\mathbf{x}_i^{(g)})^\top \right\} \boldsymbol{\delta}^{(g)} \\
&= \frac{\epsilon}{n} \|\mathbf{X}^{(g)} \boldsymbol{\delta}^{(g)}\|_2^2,
\end{aligned}$$

where $\epsilon := \exp(-2C\tilde{C})/\left(1+\exp(-2C\tilde{C})\right)^2$, and the first inequality follows from $\|\ddot{\boldsymbol{\beta}}^{(g)}\|_1 \leqslant \|\tilde{\boldsymbol{\beta}}^{(g)}\|_1 + \|\boldsymbol{\beta}^{(g)}\|_1 \leqslant 2\tilde{C}$ and $|(\mathbf{x}_i^{(g)})^\top \ddot{\boldsymbol{\beta}}^{(g)}| \leqslant \|\mathbf{x}_i^{(g)}\|_{\max} \|\ddot{\boldsymbol{\beta}}^{(g)}\|_1 \leqslant 2C\tilde{C}$, which holds due to Conditions 1 and 4. Hence, we have

$$\begin{aligned}
&\frac{\epsilon}{n}\sum_g \|\mathbf{X}^{(g)} \boldsymbol{\delta}^{(g)}\|_2^2 + g(\tilde{\boldsymbol{B}}, \tilde{\lambda}_1, \tilde{\lambda}_2) \\
&\leqslant \sum_g |\langle \nabla \ell_g(\boldsymbol{\beta}^{(g)}), \boldsymbol{\delta}^{(g)} \rangle| + g(\boldsymbol{B}, \tilde{\lambda}_1, \tilde{\lambda}_2).
\end{aligned} \tag{S1}$$

Let $\boldsymbol{\beta} := \mathrm{vec}(\boldsymbol{B}) \in \mathbb{R}^{pG}$, $\tilde{\boldsymbol{\beta}} := \mathrm{vec}(\tilde{\boldsymbol{B}}) \in \mathbb{R}^{pG}$, $\boldsymbol{\Delta} := \tilde{\boldsymbol{B}} - \boldsymbol{B} \in \mathbb{R}^{p\times G}$, and $\boldsymbol{\delta} := \mathrm{vec}(\boldsymbol{\Delta}) = \tilde{\boldsymbol{\beta}} - \boldsymbol{\beta}$. Let $\boldsymbol{\delta}^{(g)} \in \mathbb{R}^p$ be the gth vector in $\boldsymbol{\delta}$, i.e., $\boldsymbol{\delta} = [(\boldsymbol{\delta}^{(1)})^\top, \ldots, (\boldsymbol{\delta}^{(G)})^\top]^\top \in \mathbb{R}^{pG}$. Let

$$\begin{aligned}
P_{\tilde{\lambda}_1,\tilde{\lambda}_2}(\boldsymbol{B}, \tilde{\boldsymbol{B}}) := \tilde{\lambda}_1 \sum_{j=1}^p \|\boldsymbol{\beta}_{(j)}\|_2 - \tilde{\lambda}_1 \sum_{j=1}^p \|\tilde{\boldsymbol{\beta}}_{(j)}\|_2 \\
+ \tilde{\lambda}_2 \|\boldsymbol{A}\boldsymbol{\beta}\|_1 - \tilde{\lambda}_2 \|\boldsymbol{A}\tilde{\boldsymbol{\beta}}\|_1,
\end{aligned}$$

where $\boldsymbol{A}$ is the matrix defined in Lemma 1. Then, combining (S1) and the fact that $\langle \mathrm{mat}[\nabla L_n(\boldsymbol{B})], \boldsymbol{\Delta} \rangle = \langle \nabla L_n(\boldsymbol{B}), \boldsymbol{\delta} \rangle$, we get

$$\begin{aligned}
&\frac{\epsilon}{n}\|\mathbf{X}\boldsymbol{\delta}\|_2^2 \\
&\leqslant |\langle \mathrm{mat}[\nabla L_n(\boldsymbol{B})], \boldsymbol{\Delta} \rangle| + P_{\tilde{\lambda}_1,\tilde{\lambda}_2}(\boldsymbol{B}, \tilde{\boldsymbol{B}}) \\
&\leqslant \max_j \|\mathrm{mat}[\nabla L_n(\boldsymbol{B})]_{j,\cdot}\|_2 \sum_{j=1}^p \|\boldsymbol{\Delta}_{j,\cdot}\|_2 \\
&\quad + P_{\tilde{\lambda}_1,\tilde{\lambda}_2}(\boldsymbol{B}, \tilde{\boldsymbol{B}}).
\end{aligned}$$

Hence, we have by Lemma 1 and $\mathrm{mat}[\nabla L_n(\boldsymbol{B})] = [\nabla \ell_1(\boldsymbol{\beta}^{(1)}), \ldots, \nabla \ell_G(\boldsymbol{\beta}^{(G)})]$, it holds that with probability at least $1 - 1/(pG)$,

$$
\begin{aligned}
&\frac{\epsilon}{n}\|\mathbf{X}\boldsymbol{\delta}\|_2^2 \\
&\leqslant \sqrt{\frac{16G\log(pG)}{n}} \sum_{j=1}^{p} \|\boldsymbol{\Delta}_{j,\cdot}\|_2 + P_{\tilde{\lambda}_1,\tilde{\lambda}_2}(\boldsymbol{B}, \tilde{\boldsymbol{B}}) \\
&\leqslant \frac{\tilde{\lambda}_1}{2}\sum_{j=1}^{p} \|\boldsymbol{\Delta}_{j,\cdot}\|_2 + P_{\tilde{\lambda}_1,\tilde{\lambda}_2}(\boldsymbol{B}, \tilde{\boldsymbol{B}}) \\
&= \frac{\tilde{\lambda}_1}{2}\sum_{j\in S} \|\boldsymbol{\Delta}_{j,\cdot}\|_2 + \tilde{\lambda}_1\sum_{j\in S}\|\boldsymbol{\beta}_{(j)}\|_2 - \tilde{\lambda}_1\sum_{j\in S}\|\tilde{\boldsymbol{\beta}}_{(j)}\|_2 \\
&\quad + \frac{\tilde{\lambda}_1}{2}\sum_{j\in S^c} \|\boldsymbol{\Delta}_{j,\cdot}\|_2 - \tilde{\lambda}_1\sum_{j\in S^c}\|\tilde{\boldsymbol{\beta}}_{(j)}\|_2 + \tilde{\lambda}_2\|(\boldsymbol{A}\boldsymbol{\beta})_{\Omega}\|_1 \\
&\quad - \tilde{\lambda}_2\|(\boldsymbol{A}\tilde{\boldsymbol{\beta}})_{\Omega}\|_1 - \tilde{\lambda}_2\|(\boldsymbol{A}\tilde{\boldsymbol{\beta}})_{\Omega^c}\|_1, \\
&\leqslant \frac{3\tilde{\lambda}_1}{2}\sum_{j\in S} \|\boldsymbol{\Delta}_{j,\cdot}\|_2 - \frac{\tilde{\lambda}_1}{2}\sum_{j\in S^c} \|\boldsymbol{\Delta}_{j,\cdot}\|_2 + \tilde{\lambda}_2\|(\boldsymbol{A}\boldsymbol{\delta})_{\Omega}\|_1 \\
&\quad - \tilde{\lambda}_2\|(\boldsymbol{A}\boldsymbol{\delta})_{\Omega^c}\|_1,
\end{aligned}
\tag{S2}
$$

where the second inequality follows from the conditions of $\tilde{\lambda}_1$ and $\tilde{\lambda}_2$ in Theorem 1, and the third inequality follows from the triangle inequality and the fact that $\sum_{j\in S^c}\|\boldsymbol{\Delta}_{j,\cdot}\|_2 = \sum_{j\in S^c}\|\tilde{\boldsymbol{\beta}}_{(j)}\|_2$ and $\|(\boldsymbol{A}\boldsymbol{\delta})_{\Omega^c}\|_1 = \|(\boldsymbol{A}\tilde{\boldsymbol{\beta}})_{\Omega^c}\|_1$. Then, (S2) with $\tilde{\lambda}_2 = G^{-3/2}\tilde{\lambda}_1$ gives the following inequality:

$$
\begin{aligned}
&G\sqrt{G}\sum_{j\in S^c}\|\boldsymbol{\Delta}_{j,\cdot}\|_2 + \|(\boldsymbol{A}\boldsymbol{\delta})_{\Omega^c}\|_1 \\
&\qquad \leqslant 3G\sqrt{G}\sum_{j\in S}\|\boldsymbol{\Delta}_{j,\cdot}\|_2 + 3\|(\boldsymbol{A}\boldsymbol{\delta})_{\Omega}\|_1.
\end{aligned}
$$

Using the restricted strong convexity presented in Condition 2, we obtain

$$
\begin{aligned}
\tilde{c}\epsilon\|\boldsymbol{\delta}\|_2^2 \leqslant \frac{\epsilon}{n}\|\mathbf{X}\boldsymbol{\delta}\|_2^2 &\leqslant 1.5\tilde{\lambda}_1\sum_{j\in S}\|\boldsymbol{\Delta}_{j,\cdot}\|_2 + \tilde{\lambda}_2\|(\boldsymbol{A}\boldsymbol{\delta})_{\Omega}\|_1 \\
&\leqslant 1.5\tilde{\lambda}_1\sqrt{s}\|\boldsymbol{\delta}\|_2 + \tilde{\lambda}_2\sqrt{\tilde{s}}\|\boldsymbol{A}\boldsymbol{\delta}\|_2 \\
&\leqslant \frac{9}{4\tilde{c}\epsilon}\tilde{\lambda}_1^2 s + \frac{4}{\tilde{c}\epsilon}\tilde{\lambda}_2^2 s + \frac{1}{2}\tilde{c}\epsilon\|\boldsymbol{\delta}\|_2^2,
\end{aligned}
$$

where the third inequality follows from the fact that $ab \leqslant a^2 + b^2$ for any numbers a and b and the fact that $\|\boldsymbol{A}\boldsymbol{\delta}\|_2 \leqslant 2\|\boldsymbol{\delta}\|_2$. This gives

$$
\|\boldsymbol{\delta}\|_2^2 \leqslant \frac{9}{2\tilde{c}\epsilon}\tilde{\lambda}_1^2 s + \frac{8}{\tilde{c}\epsilon}\tilde{\lambda}_2^2\tilde{s}.
$$

Combining with the definitions of $\tilde{\lambda}_1$ and $\tilde{\lambda}_2$, this completes the proof. □

Proof of Theorem 2

Note that the rate conditions of λ_1 and λ_2 in Theorem 2, Condition 4 and Theorem 1 imply

$$\begin{aligned}
&\sqrt{\frac{G\log(s+G)}{n}} = o\big(\lambda_1 w_{\min}\big),\ \sqrt{\frac{64\log(pG)}{nG^2}} < \lambda_2 \tilde{v}_{\max} \\
&G^{1/2} = o(\frac{v_{\min}}{\tilde{v}_{\max}}),\ s^{1/2} = o(\frac{w_{\min}}{\tilde{w}_{\max}}),\ \lambda_2 \tilde{s}^{1/2} = o(\lambda_1 \frac{w_{\min}}{\tilde{v}_{\max}}), \\
&\sqrt{\frac{G\log(sG)}{n}} \vee \lambda_1 \tilde{w}_{\max} G^{1/2} \vee \lambda_2 \tilde{v}_{\max} \sqrt{\frac{\tilde{s}G}{s}} = o(\lambda_2 v_{\min}).
\end{aligned} \tag{S3}$$

Suppose that $\hat{\boldsymbol{B}}_{j,\cdot} \neq 0$ for some $j \in S^c$. Denote $\ddot{\boldsymbol{B}}$ by $\ddot{\boldsymbol{B}}_{l,\cdot} = \hat{\boldsymbol{B}}_{l,\cdot}$ for $l \neq j$ and $\ddot{\boldsymbol{B}}_{j,\cdot} = \mathbf{0}_G$. Because $L_n(\cdot)$ is convex, we have

$$\begin{aligned}
L_n(\hat{\boldsymbol{B}}) &\geqslant L_n(\ddot{\boldsymbol{B}}) + \langle \text{mat}[\nabla L_n(\ddot{\boldsymbol{B}})], \hat{\boldsymbol{B}} - \ddot{\boldsymbol{B}} \rangle \\
&\geqslant L_n(\ddot{\boldsymbol{B}}) - \|\text{mat}[\nabla L_n(\ddot{\boldsymbol{B}})]_{j,\cdot}\|_2 \, \|\hat{\boldsymbol{B}}_{j,\cdot} - \ddot{\boldsymbol{B}}_{j,\cdot}\|_2.
\end{aligned} \tag{S4}$$

Let $\ddot{\boldsymbol{Q}} = \int_0^1 \nabla^2 L_n\big(\boldsymbol{B} + t(\ddot{\boldsymbol{B}} - \boldsymbol{B})\big)dt$, where $\ddot{\boldsymbol{Q}}$ is a $pG \times pG$ block-diagonal matrix, consisting of G different $p \times p$ sub-matrices $\ddot{\boldsymbol{Q}}^{(g)}$. Specifically, we can write $\ddot{\boldsymbol{Q}} := \text{diag}(\ddot{\boldsymbol{Q}}^{(1)}, \ldots, \ddot{\boldsymbol{Q}}^{(G)})$ for some $\tilde{\boldsymbol{\beta}}^{(g)}$, where

$$\begin{aligned}
\ddot{\boldsymbol{Q}}^{(g)} &:= \sum_{i=1}^{n_g} \frac{1}{n} e_i^{(g)} \mathbf{x}_i^{(g)} (\mathbf{x}_i^{(g)})^\top \\
e_i^{(g)} &:= \frac{\exp\big(y_i^{(g)} (\mathbf{x}_i^{(g)})^\top \tilde{\boldsymbol{\beta}}^{(g)}\big)}{\Big(1 + \exp\big(y_i^{(g)} (\mathbf{x}_i^{(g)})^\top \tilde{\boldsymbol{\beta}}^{(g)}\big)\Big)^2}.
\end{aligned}$$

Let $\boldsymbol{\delta}^{(g)} := \ddot{\boldsymbol{B}}_{\cdot,g} - \boldsymbol{B}_{\cdot,g} \in \mathbb{R}^p$. Because $\delta_l^{(g)} = 0$ for $l \neq j$, we have

$$|\ddot{\boldsymbol{Q}}_{j,\cdot}^{(g)} \boldsymbol{\delta}^{(g)}| = \frac{1}{n} \left| \sum_{i=1}^{n_g} e_i^{(g)} \big(x_{ij}^{(g)}\big)^2 \delta_j^{(g)} \right| \leqslant \|\boldsymbol{\delta}^{(g)}\|_2,$$

where the last equality follows from the fact that $0 \leqslant e_i^{(g)} \leqslant 1/4$ and $\sum_{i=1}^{n_g} \big(x_{ij}^{(g)}\big)^2 = n_g$.

Hence, by Lemma 1, we have with probability at least $1 - 1/(pG)$,

$$\begin{aligned}
&\max_{j\in S^c} \|\mathrm{mat}[\nabla L_n(\ddot{\boldsymbol{B}})]_{j,\cdot}\|_2 \\
&\leqslant \max_{j\in S^c} \|\mathrm{mat}[\nabla L_n(\boldsymbol{B})]_{j,\cdot}\|_2 + \max_{1\leqslant g\leqslant G}\max_{j\in S^c} \left|\ddot{\boldsymbol{Q}}^{(g)}_{j,\cdot}\boldsymbol{\delta}^{(g)}\right| \\
&\leqslant \sqrt{\frac{16G\log(s+G)}{n}} + \max_{1\leqslant g\leqslant G}\|\boldsymbol{\delta}^{(g)}\|_2 \\
&\leqslant \sqrt{\frac{16G\log(s+G)}{n}} + \frac{\sqrt{18}}{\tilde{c}\epsilon}\Big(\lambda_1\tilde{w}_{\max}s^{1/2} \\
&\qquad + \lambda_2\tilde{v}_{\max}\tilde{s}^{1/2}\Big) \\
&=: \mathrm{I}(\lambda_1,\lambda_2)
\end{aligned}$$

where the third inequality follows from Lemma 2.

Combining with (S4), we obtain

$$L_n(\hat{\boldsymbol{B}}) \geqslant L_n(\ddot{\boldsymbol{B}}) - I(\lambda_1,\lambda_2)\cdot\|\hat{\boldsymbol{B}}_{j,\cdot} - \ddot{\boldsymbol{B}}_{j,\cdot}\|_2. \tag{S5}$$

Further, by the definition of $\ddot{\boldsymbol{B}}$, we have

$$\begin{aligned}
&\Big(\lambda_1\sum_{j=1}^p w_j\|\hat{\boldsymbol{B}}_{j\cdot}\|_2 + \lambda_2\sum_{j=1}^p\sum_{g<g'} v_{j,gg'}|\hat{B}_{jg} - \hat{B}_{jg'}|\Big) \\
&\ - \Big(\lambda_1\sum_{j=1}^p w_j\|\ddot{\boldsymbol{B}}_{j\cdot}\|_2 + \lambda_2\sum_{j=1}^p\sum_{g<g'} v_{j,gg'}|\ddot{B}_{jg} - \ddot{B}_{jg'}|\Big) \\
&\geqslant \lambda_1\sum_{j=1}^p w_j\|\hat{\boldsymbol{B}}_{j,\cdot}\|_2 - \lambda_1\sum_{j=1}^p w_j\|\ddot{\boldsymbol{B}}_{j,\cdot}\|_2 \\
&\geqslant \lambda_1 w_{\min}\|\hat{\boldsymbol{B}}_{j,\cdot} - \ddot{\boldsymbol{B}}_{j,\cdot}\|_2,
\end{aligned} \tag{S6}$$

where the last inequality follows from $\ddot{\boldsymbol{B}}_{j,\cdot} = \mathbf{0}_G$ and $\ddot{\boldsymbol{B}}_{l,\cdot} = \hat{\boldsymbol{B}}_{l,\cdot}$ for $l \neq j$. Because $\lambda_1 w_{\min} > I(\lambda_1,\lambda_2)$ by (S3), (S5)-(S6) imply that the objective value of $\ddot{\boldsymbol{B}}$ is less than that of $\hat{\boldsymbol{B}}$, which contradicts to the fact that $\hat{\boldsymbol{B}}$ is the minimizer. Thus, we have

$$\hat{\boldsymbol{B}}_{j,\cdot} = \mathbf{0}_G \quad \text{for} \quad j \in S^c. \tag{S7}$$

Next, for some $j \in S$ and $d \leqslant G$, define the set $A := \{g_1,\ldots,g_d\} \subseteq \{1,\ldots,G\}$ such that $B_{j,g_1} = \ldots = B_{j,g_d}$ and $B_{j,g_k} \neq B_{j,l}$ for $l \notin A$. Suppose that $\hat{B}_{j,g_k} \neq \hat{B}_{j,g_l}$ for some $g_k, g_l \in A$. Denote $\tilde{\boldsymbol{B}} = (\tilde{\beta}_{jg})_{1\leqslant j\leqslant p, 1\leqslant g\leqslant G}$ by $\tilde{\boldsymbol{B}}_{\tilde{j},\cdot} = \hat{\boldsymbol{B}}_{\tilde{j},\cdot}$ for $\tilde{j} \neq j$, and $\tilde{\beta}_{jg} = \sum_{l=1}^d \hat{\beta}_{j,g_l}/d$ for $g \in A$ and $\tilde{\beta}_{jg'} = \hat{\beta}_{jg'}$ for $g' \notin A$. Because $L_n(\cdot)$ is convex, we have

$$\begin{aligned}
L_n(\hat{\boldsymbol{B}}) &\geqslant L_n(\tilde{\boldsymbol{B}}) + \langle\mathrm{mat}[\nabla L_n(\tilde{\boldsymbol{B}})], \hat{\boldsymbol{B}} - \tilde{\boldsymbol{B}}\rangle \\
&\geqslant L_n(\tilde{\boldsymbol{B}}) - \|\mathrm{mat}[\nabla L_n(\tilde{\boldsymbol{B}})]_{j,\cdot}\|_{\max}\cdot\|\hat{\boldsymbol{B}} - \tilde{\boldsymbol{B}}\|_1.
\end{aligned}$$

Let

$$\tilde{\boldsymbol{Q}} = \int_0^1 \nabla^2 L_n\big(\boldsymbol{B} + t(\tilde{\boldsymbol{B}} - \boldsymbol{B})\big)dt,$$

where $\tilde{\boldsymbol{Q}}$ is a $pG \times pG$ block-diagonal matrix, consisting of G different $p \times p$ sub-matrices $\tilde{\boldsymbol{Q}}^{(g)}$. Specifically, we can write $\tilde{\boldsymbol{Q}} := \mathrm{diag}(\tilde{\boldsymbol{Q}}^{(1)}, \ldots, \tilde{\boldsymbol{Q}}^{(G)})$ for some $\tilde{\boldsymbol{\beta}}^{(g)}$, where

$$\tilde{\boldsymbol{Q}}^{(g)} := \sum_{i=1}^{n_g} \frac{1}{n} e_i^{(g)} \mathbf{x}_i^{(g)} (\mathbf{x}_i^{(g)})^\top$$

$$e_i^{(g)} := \frac{\exp\big(y_i^{(g)} (\mathbf{x}_i^{(g)})^\top \tilde{\boldsymbol{\beta}}^{(g)}\big)}{\Big(1 + \exp(y_i^{(g)} (\mathbf{x}_i^{(g)})^\top \tilde{\boldsymbol{\beta}}^{(g)})\Big)^2}.$$

Let $\boldsymbol{\delta}^{(k)} := \tilde{\boldsymbol{B}}_{\cdot,k} - \boldsymbol{B}_{\cdot,k} \in \mathbb{R}^p$. Because $\boldsymbol{\delta}_{S^c}^{(k)} = \mathbf{0}_{p-s}$, we have

$$\begin{aligned}
|\tilde{\boldsymbol{Q}}_{j,\cdot}^{(k)} \boldsymbol{\delta}^{(k)}| &= \frac{1}{n} \left| \sum_{i=1}^{n_k} e_i^{(k)} x_{ij}^{(k)} \mathbf{x}_{iS}^{(k)} \boldsymbol{\delta}_S^{(k)} \right| \\
&\leqslant \frac{n_k}{n} \left[\sum_{i=1}^{n_k} \left\{ e_i^{(k)} x_{ij}^{(k)} \mathbf{x}_{iS}^{(k)} \boldsymbol{\delta}_S^{(k)} \right\}^2 / n_k \right]^{1/2} \\
&\leqslant C c_{\max}^{1/2} \|\boldsymbol{\delta}_S^{(k)}\|_2,
\end{aligned}$$

where the last inequality follows from Condition 1.

Applying the proof of Lemma 1, we thus have with probability at least $1 - 1/(sG)$,

$$\begin{aligned}
&\max_{1 \leqslant g \leqslant G} |\mathrm{mat}[\nabla L_n(\tilde{\boldsymbol{B}})]_{jg}| \\
&\leqslant \max_{1 \leqslant g \leqslant G} |\mathrm{mat}[\nabla L_n(\boldsymbol{B})]_{jg}| + \max_{1 \leqslant g \leqslant G} |\mathrm{mat}[\tilde{\boldsymbol{Q}}(\tilde{\boldsymbol{B}} - \boldsymbol{B})]_{jg}| \\
&\leqslant \sqrt{\frac{16 \log(sG)}{n}} + C c_{\max}^{1/2} \cdot \max_{1 \leqslant g \leqslant G} \|\boldsymbol{\delta}_S^{(g)}\|_2 \\
&\leqslant \sqrt{\frac{16 \log(sG)}{n}} + \frac{\sqrt{18} C c_{\max}^{1/2}}{\tilde{c}\epsilon} \Big(\lambda_1 s^{1/2} \tilde{w}_{\max} + \lambda_2 \tilde{v}_{\max} \tilde{s}^{1/2} \Big) \\
&=: \text{II}(\lambda_1, \lambda_2),
\end{aligned}$$

where the last inequality follows from Lemma 2. Hence, we have

$$\begin{aligned}
L_n(\hat{\boldsymbol{B}}) &\geqslant L_n(\tilde{\boldsymbol{B}}) - \text{II}(\lambda_1, \lambda_2) \|\hat{\boldsymbol{B}}_{j,\cdot} - \tilde{\boldsymbol{B}}_{j,\cdot}\|_1 \\
&\geqslant L_n(\tilde{\boldsymbol{B}}) - \text{II}(\lambda_1, \lambda_2) G^{1/2} \|\hat{\boldsymbol{B}}_{j,\cdot} - \tilde{\boldsymbol{B}}_{j,\cdot}\|_2.
\end{aligned} \tag{S8}$$

Because $\tilde{\boldsymbol{B}}_{\tilde{j},\cdot} = \hat{\boldsymbol{B}}_{\tilde{j},\cdot}$ for $\tilde{j} \neq j$ and $\sum_{l=1}^d \hat{B}_{j,g_l}^2 \geqslant \sum_{l=1}^d \tilde{B}_{j,g_l}^2$, it holds that

$$\lambda_1 \sum_{j=1}^p w_j \|\hat{\boldsymbol{B}}_{j,\cdot}\|_2 \geqslant \lambda_1 \sum_{j=1}^p w_j \|\tilde{\boldsymbol{B}}_{j,\cdot}\|_2.$$

Further, by simple algebra, we have

$$
\begin{aligned}
d \cdot \|\hat{\boldsymbol{B}}_{j,\cdot} - \tilde{\boldsymbol{B}}_{j,\cdot}\|_2^2 &= d \cdot \sum_{l=1}^{d} (\hat{\beta}_{j,g_l} - \tilde{\beta}_{jg_l})^2 \\
&\leqslant \sum_{l,l' \in A} (\hat{\beta}_{j,g_l} - \hat{\beta}_{j,g_{l'}})^2.
\end{aligned}
$$

Thus, it holds that $\sum_{l,l' \in A} |\hat{\beta}_{j,g_l} - \hat{\beta}_{j,g_{l'}}| \geqslant \sqrt{d}\|\hat{\boldsymbol{B}}_{j,\cdot} - \tilde{\boldsymbol{B}}_{j,\cdot}\|_2$. Let $d_g := |\tilde{\beta}_{jg} - \hat{\beta}_{jg}|$. Note that $\tilde{\beta}_{jg} - \hat{\beta}_{jg} = 0$ for $g \notin A$. Then, for $g \notin A$ and $g' \in A$, we have

$$
|\tilde{\beta}_{jg} - \tilde{\beta}_{jg'}| \leqslant |\hat{\beta}_{jg} - \hat{\beta}_{jg'}| + d_{g'}.
$$

Combining these inequalities, we obtain

$$
\begin{aligned}
&\lambda_2 \sum_{j=1}^{p} \sum_{g<g'} v_{j,gg'} |\hat{\beta}_{jg} - \hat{\beta}_{jg'}| - \lambda_2 \sum_{j=1}^{p} \sum_{g<g'} v_{j,gg'} |\tilde{\beta}_{jg} - \tilde{\beta}_{jg'}| \\
&= \lambda_2 \sum_{g,g' \in A} v_{j,gg'} |\hat{\beta}_{jg} - \hat{\beta}_{jg'}| - \lambda_2 \sum_{g,g' \in A} v_{j,gg'} |\tilde{\beta}_{jg} - \tilde{\beta}_{jg'}| \\
&+ \lambda_2 \sum_{g \notin A, g' \in A} v_{j,gg'} |\hat{\beta}_{jg} - \hat{\beta}_{jg'}| - \lambda_2 \sum_{g \notin A, g' \in A} v_{j,gg'} |\tilde{\beta}_{jg} - \tilde{\beta}_{jg'}| \\
&+ \lambda_2 \sum_{g \in A, g' \notin A} v_{j,gg'} |\hat{\beta}_{jg} - \hat{\beta}_{jg'}| - \lambda_2 \sum_{g \in A, g' \notin A} v_{j,gg'} |\tilde{\beta}_{jg} - \tilde{\beta}_{jg'}| \\
&\geqslant \lambda_2 v_{\min} \sqrt{d} \|\hat{\boldsymbol{B}}_{j,\cdot} - \tilde{\boldsymbol{B}}_{j,\cdot}\|_2 - 2\lambda_2 \tilde{v}_{\max} \sum_{g \in A} |d_g| \\
&\geqslant \lambda_2 v_{\min} \sqrt{d} \|\hat{\boldsymbol{B}}_{j,\cdot} - \tilde{\boldsymbol{B}}_{j,\cdot}\|_2 - 2\lambda_2 \tilde{v}_{\max} \sqrt{d} \|\hat{\boldsymbol{B}}_{j,\cdot} - \tilde{\boldsymbol{B}}_{j,\cdot}\|_2 \\
&> \text{II}(\lambda_1, \lambda_2) G^{1/2} \|\hat{\boldsymbol{B}}_{j,\cdot} - \tilde{\boldsymbol{B}}_{j,\cdot}\|_2,
\end{aligned}
$$

where the second inequality follows from $\sum_{g \in A} |d_g|^2 = \|\hat{\boldsymbol{B}}_{j,\cdot} - \tilde{\boldsymbol{B}}_{j,\cdot}\|_2^2$ and the last inequality follows from (S3). Combining with (S8), this implies that the objective value of $\tilde{\boldsymbol{B}}$ is less than that of $\hat{\boldsymbol{B}}$, which contradicts to the fact that $\hat{\boldsymbol{B}}$ is the minimizer. Thus, $\hat{\beta}_{j,g_1} = \ldots = \hat{\beta}_{j,g_d}$. Combining with (S7), and the minimum signal and minimum signal difference conditions in Condition 4, we obtain that $\hat{\boldsymbol{B}}$ is the oracle estimator, i.e., $P(\hat{S} = S) \to 1$ and $P(\hat{\Omega} = \Omega) \to 1$. Thus, $\hat{\boldsymbol{B}} = \hat{\boldsymbol{B}}^{ora}$, where $\hat{\boldsymbol{B}}^{ora} := \left[(\hat{\boldsymbol{B}}_S^{ora})^\top, \mathbf{0}_{G,p-s}\right]^\top$. This completes the proof. $\square$

For any $y \in \{0, 1\}$ and $\nu \in \mathbb{R}$, recall that $\ell(y, \nu) = -y\nu + \log(1 + \exp(\nu))$. For the loss function $\ell(y, \nu)$, let $\dot{\ell}(y, \nu)$ and $\ddot{\ell}(y, \nu)$ denote its first and second derivatives with respect to ν, respectively. We obtain that the optimization (7) in the main paper is feasible. Specifically, $[(\boldsymbol{\Sigma}^{(\boldsymbol{g})})^{-1}]_{j,\cdot}$ is a feasible solution.

Proof of Theorem 3

Recall that the debiased estimator for $\boldsymbol{\beta}^{(g)}$ is

$$\hat{\boldsymbol{b}}^{(g)} := \hat{\boldsymbol{\beta}}^{(g)} - \frac{\hat{\boldsymbol{M}}^{(\boldsymbol{g})}}{n_g} \sum_{i=1}^{n_g} \dot{\ell}(y_i^{(g)}, (\mathbf{x}_i^{(g)})^\top \hat{\boldsymbol{\beta}}^{(g)}) \mathbf{x}_i^{(g)}. \tag{S9}$$

Note that

$$\begin{aligned} \dot{\ell}(y_i^{(g)}, (\mathbf{x}_i^{(g)})^\top \hat{\boldsymbol{\beta}}^{(g)}) &= \dot{\ell}(y_i^{(g)}, (\mathbf{x}_i^{(g)})^\top \boldsymbol{\beta}^{(g)}) \\ &\quad + \ddot{\ell}(y_i^{(g)}, \tilde{a}_{gi})(\mathbf{x}_i^{(g)})^\top (\hat{\boldsymbol{\beta}}^{(g)} - \boldsymbol{\beta}^{(g)}), \end{aligned}$$

where $\tilde{a}_{gi}$ is a point between $(\mathbf{x}_i^{(g)})^\top \hat{\boldsymbol{\beta}}^{(g)}$ and $(\mathbf{x}_i^{(g)})^\top \boldsymbol{\beta}^{(g)}$, i.e., $|\tilde{a}_{gi} - (\mathbf{x}_i^{(g)})^\top \hat{\boldsymbol{\beta}}^{(g)}| \leqslant |(\mathbf{x}_i^{(g)})^\top (\hat{\boldsymbol{\beta}}^{(g)} - \boldsymbol{\beta}^{(g)})|$. Let

$$\begin{aligned} e_{gi} := \frac{1}{n_g} [\ddot{\ell}(y_i^{(g)}, \tilde{a}_{g,i}) - \ddot{\ell}(y_i^{(g)}, (\mathbf{x}_i^{(g)})^\top \hat{\boldsymbol{\beta}}^{(g)})] \\ \cdot (\mathbf{x}_i^{(g)})^\top (\boldsymbol{\beta}^{(g)} - \hat{\boldsymbol{\beta}}^{(g)}). \end{aligned}$$

Then, we have

$$\begin{aligned} |e_{gi}| &\leqslant \frac{1}{n_g} \left|\tilde{a}_{g,i} - (\mathbf{x}_i^{(g)})^\top \hat{\boldsymbol{\beta}}^{(g)}\right| \cdot \left|(\mathbf{x}_i^{(g)})^\top (\hat{\boldsymbol{\beta}}^{(g)} - \boldsymbol{\beta}^{(g)})\right| \\ &\leqslant \frac{1}{n_g} |(\mathbf{x}_i^{(g)})^\top (\hat{\boldsymbol{\beta}}^{(g)} - \boldsymbol{\beta}^{(g)})|^2. \end{aligned}$$

Then, from (S9), we can write

$$\begin{aligned} &\hat{\boldsymbol{b}}^{(g)} - \boldsymbol{\beta}^{(g)} \\ &= \hat{\boldsymbol{\beta}}^{(g)} - \boldsymbol{\beta}^{(g)} - \frac{\hat{\boldsymbol{M}}^{(\boldsymbol{g})}}{n_g} \sum_{i=1}^{n_g} \dot{\ell}(y_i^{(g)}, (\mathbf{x}_i^{(g)})^\top \boldsymbol{\beta}^{(g)}) \mathbf{x}_i^{(g)} \\ &\quad - \frac{\hat{\boldsymbol{M}}^{(\boldsymbol{g})}}{n_g} \sum_{i=1}^{n_g} \ddot{\ell}(y_i^{(g)}, \tilde{a}_{g,i}) \mathbf{x}_i^{(g)} (\mathbf{x}_i^{(g)})^\top (\hat{\boldsymbol{\beta}}^{(g)} - \boldsymbol{\beta}^{(g)}) \\ &= \hat{\boldsymbol{\beta}}^{(g)} - \boldsymbol{\beta}^{(g)} - \frac{\hat{\boldsymbol{M}}^{(\boldsymbol{g})}}{n_g} \sum_{i=1}^{n_g} \dot{\ell}(y_i^{(g)}, (\mathbf{x}_i^{(g)})^\top \boldsymbol{\beta}^{(g)}) \mathbf{x}_i^{(g)} \\ &\quad - \hat{\boldsymbol{M}}^{(\boldsymbol{g})} \hat{\boldsymbol{\Sigma}}^{(\boldsymbol{g})} (\hat{\boldsymbol{\beta}}^{(g)} - \boldsymbol{\beta}^{(g)}) - \hat{\boldsymbol{M}}^{(\boldsymbol{g})} \sum_{i=1}^{n_g} e_{gi} \mathbf{x}_i^{(g)} \\ &= -\frac{\hat{\boldsymbol{M}}^{(\boldsymbol{g})}}{n_g} \sum_{i=1}^{n_g} \dot{\ell}(y_i^{(g)}, (\mathbf{x}_i^{(g)})^\top \boldsymbol{\beta}^{(g)}) \mathbf{x}_i^{(g)} \\ &\quad + (\boldsymbol{I} - \hat{\boldsymbol{M}}^{(\boldsymbol{g})} \hat{\boldsymbol{\Sigma}}^{(\boldsymbol{g})}) (\hat{\boldsymbol{\beta}}^{(g)} - \boldsymbol{\beta}^{(g)}) - \hat{\boldsymbol{M}}^{(\boldsymbol{g})} \sum_{i=1}^{n_g} e_{gi} \mathbf{x}_i^{(g)}. \end{aligned}$$

Then, we obtain

$$
\begin{aligned}
&\sqrt{n_g}(\hat{\boldsymbol{b}}^{(g)} - \boldsymbol{\beta}^{(g)}) \\
&= \underbrace{-\frac{\hat{\boldsymbol{M}}^{(\boldsymbol{g})}}{\sqrt{n_g}} \sum_{i=1}^{n_g} \dot{\ell}(y_i^{(g)}, (\mathbf{x}_i^{(g)})^\top \boldsymbol{\beta}^{(g)}) \mathbf{x}_i^{(g)}}_{\mathrm{I}_1^{(g)}} \\
&\quad + \underbrace{(\boldsymbol{I} - \hat{\boldsymbol{M}}^{(\boldsymbol{g})} \hat{\boldsymbol{\Sigma}}^{(\boldsymbol{g})}) \sqrt{n_g}(\hat{\boldsymbol{\beta}}^{(g)} - \boldsymbol{\beta}^{(g)})}_{\mathrm{I}_2^{(g)}} \\
&\underbrace{- \sqrt{n_g} \hat{\boldsymbol{M}}^{(\boldsymbol{g})} \sum_{i=1}^{n_g} e_{gi} \mathbf{x}_i^{(g)}}_{\mathrm{I}_3^{(g)}}.
\end{aligned}
$$

Because $\mu_g \asymp \sqrt{\log p / n_g}$, we have

$$
\begin{aligned}
&\max_{1 \leqslant g \leqslant G} \|\mathrm{I}_2^{(g)}\|_{\max} \\
&\leqslant \max_{1 \leqslant g \leqslant G} \sqrt{n_g} \|\boldsymbol{I} - \hat{\boldsymbol{M}}^{(\boldsymbol{g})} \hat{\boldsymbol{\Sigma}}^{(\boldsymbol{g})}\|_{\max} \cdot \|\hat{\boldsymbol{\beta}}^{(g)} - \boldsymbol{\beta}^{(g)}\|_1 \\
&= O_p\left(\max_g \sqrt{n_g} \mu_g s G \sqrt{\frac{\log(sG)}{n}} \right) \\
&= o_p(1),
\end{aligned}
$$

where the first equality follows from Lemma 3 and the last equality follows from Condition 5. Further, we have

$$
\begin{aligned}
&\max_{1 \leqslant g \leqslant G} \|\mathrm{I}_3^{(g)}\|_{\max} \\
&\leqslant \max_{1 \leqslant g \leqslant G} \frac{1}{\sqrt{n_g}} \sum_{i=1}^{n_g} |(\mathbf{x}_i^{(g)})^\top (\hat{\boldsymbol{\beta}}^{(g)} - \boldsymbol{\beta}^{(g)})|^2 \\
&\qquad\qquad \cdot \|\hat{\boldsymbol{M}}^{(\boldsymbol{g})}\|_1 \cdot \|\mathbf{x}_i^{(g)}\|_{max} \\
&\leqslant \max_{1 \leqslant g \leqslant G} \frac{1}{\sqrt{n_g}} \sum_{i=1}^{n_g} \|\mathbf{x}_i^{(g)}\|_{\max}^3 \cdot \|\hat{\boldsymbol{\beta}}^{(g)} - \boldsymbol{\beta}^{(g)}\|_1^2 \cdot \|\hat{\boldsymbol{M}}^{(\boldsymbol{g})}\|_1 \\
&= O_p\left(\max_g \left(\|\mathbf{X}^{(g)}\|_{\max}^3 \sqrt{n_g} \right) s_0 s^2 G^2 \frac{\log(sG)}{n} \right) \\
&= o_p(1),
\end{aligned}
$$

where the equality in the third line holds due to Condition 5 and the fact that

$$\begin{aligned}\|\hat{\boldsymbol{M}}^{(\boldsymbol{g})}\|_1 &\leqslant \|\hat{\boldsymbol{M}}^{(\boldsymbol{g})} - \boldsymbol{M}^{(\boldsymbol{g})}\|_1 + \|\boldsymbol{M}^{(g)}\|_1 \\ &= O_p\left(s_0\sqrt{\log p/n_g} + s_0\right) \\ &= O_p(s_0)\end{aligned}$$

as shown in the proof of Theorem 3.2 in Van de Geer et al. [2], where the last equality follows from Conditions 1 and 5. This completes the proof. □

Recall that $\hat{V}^{(g)} = \hat{M}^{(g)}\hat{\boldsymbol{\Sigma}}^{(\boldsymbol{g})}(\hat{\boldsymbol{M}}^{(\boldsymbol{g})})^\top$ and $\hat{V}_{(j)}$ be the $G \times G$ diagonal matrix with diagonal elements $\{\frac{1}{n_g}\hat{V}_{jj}^{(g)}\}_{g=1}^G$. Define $\boldsymbol{S}_j := \hat{\boldsymbol{V}}_{(\boldsymbol{j})}^{-\boldsymbol{1/2}}(\hat{\boldsymbol{b}}_{(j)} - \boldsymbol{\beta}_{(j)}) = [S_{j1}, \ldots, S_{jG}]^\top$. Below we only show the proofs of Theorem 4 and Corollary 1. Proofs of Theorem 5 and Corollary 2 are the same in principle.

Proof of Theorem 4 and Corollary1

By Theorem 3, we have for $1 \leqslant g \leqslant G$,

$$\begin{aligned}S_{jg} &= [\hat{V}_{jj}^{(g)}]^{-1/2}\sqrt{n_g}(\hat{b}_j^{(g)} - \beta_j^{(g)}) \\ &= -[\hat{V}_{jj}^{(g)}]^{-1/2}\frac{\hat{\boldsymbol{M}}_{\boldsymbol{j},\cdot}^{(\boldsymbol{g})}}{\sqrt{n_g}}\sum_{i=1}^{n_g}\dot{\ell}(y_i^{(g)}, (\mathbf{x}_i^{(g)})^\top\boldsymbol{\beta}^{(g)})\mathbf{x}_i^{(g)} \\ &\quad + o_p(1).\end{aligned}$$

Thus, we can only consider the first part of S_{jg} in the distribution. We can write $S_j = \sum_{i=1}^{\max_g n_g} \boldsymbol{Z}_i$, where $\boldsymbol{Z}_i$'s are independent, $E[\boldsymbol{Z}_i] = 0_G$, and $\sum_{i=1}^{\max_g n_g} \mathrm{var}(\boldsymbol{Z}_i) = I_G$. By the Berry-Essen Theorem for sums of independent random vectors [3],

$$|P(S_j \in A) - \Phi(A)| \leqslant (42G^{1/4} + 16)\sum_{i=1}^{\max_g n_g} E\|\boldsymbol{Z}_i\|_2^3.$$

for all measurable convex sets $A \subseteq \mathbb{R}^G$. Note that we have for some absolute constant $C_3 > 0$,

$$\begin{aligned}\sum_{i=1}^{\max_g n_g} E\|\boldsymbol{Z}_i\|_2^3 &\leqslant (\max_i \|\boldsymbol{Z}_i\|_2)\sum_{i=1}^{\max_g n_g} E\|\boldsymbol{Z}_i\|_2^2 \\ &= (\max_i \|\boldsymbol{Z}_i\|_2)\sum_{i=1}^{\max_g n_g} E[\mathrm{tr}(\boldsymbol{Z}_i\boldsymbol{Z}_i^\top)] \\ &= (\max_i \|\boldsymbol{Z}_i\|_2)\sum_{i=1}^{\max_g n_g} \mathrm{tr}(\mathrm{var}(\boldsymbol{Z}_i)) \\ &= G\max_i \|\boldsymbol{Z}_i\|_2 \\ &\leqslant G^{3/2}CC_3 s_0/\sqrt{\min_{1\leqslant g\leqslant G} n_g},\end{aligned}$$

where the last inequality holds due to the fact that

$$\begin{aligned}
&\left|[\hat{V}^{(g)}]_{jj}^{-1/2}\frac{\hat{[\hat{\boldsymbol{M}}^{(g)}]}_{j,\cdot}}{\sqrt{n_g}}\dot{\ell}(y_i^{(g)},(x_i^{(g)})^\top\boldsymbol{\beta}^{(g)})\mathbf{x}_i^{(g)}\right|\\
&\leqslant [\hat{V}^{(g)}]_{jj}^{-1/2}\frac{|\hat{[\hat{\boldsymbol{M}}^{(g)}]}_{j,\cdot}\mathbf{x}_i^{(g)}|}{\sqrt{n_g}}\\
&\leqslant CC_3 s_0/\sqrt{\min_{1\leqslant g\leqslant G} n_g}.
\end{aligned}$$

Hence, we have

$$|P(\boldsymbol{S}_j\in A)-\Phi(A)|\leqslant 42CC_3G^{7/4}s_0/\sqrt{\min_{1\leqslant g\leqslant G} n_g}.$$

Define the set A by

$$A:=\{\boldsymbol{v}\in\mathbb{R}^G:\ (\|\boldsymbol{v}\|_2^2-G)/\sqrt{2G}\leqslant t\}.$$

Then, the set A is a measurable convex set, thus we have that for any t,

$$\begin{aligned}
&\left|P\left(\frac{\|\boldsymbol{S}_j\|_2^2-G}{\sqrt{2G}}\leqslant t\right)-P\left(\frac{\chi_G^2-G}{\sqrt{2G}}\leqslant t\right)\right|\\
&\leqslant 42CC_3G^{7/4}s_0/\sqrt{\min_{1\leqslant g\leqslant G} n_g},
\end{aligned}$$

where χ_G^2 is Chi-squared distributed with a degree of freedom G. Thus, we obtain that for any t,

$$\begin{aligned}
&\left|P\left(\|\boldsymbol{S}_j\|_2^2\leqslant t\right)-P\left(\chi_G^2\leqslant t\right)\right|\\
&\leqslant 42CC_3G^{7/4}s_0/\sqrt{\min_{1\leqslant g\leqslant G} n_g}\\
&=o(1).
\end{aligned}$$

This completes the proof. □

S2 Additional Lemmas

The following Lemma 2 presents an estimation error bound for $\hat{\boldsymbol{B}}$. Note that in Theorem 2, we prove that $\hat{\boldsymbol{B}}$ is equivalent to the oracle estimator $\hat{\boldsymbol{B}}_S^{gra}$ defined at (S11) with probability tending to one. Let $\epsilon:=\exp(-2C\tilde{C})/\Big(1+\exp(-2C\tilde{C})\Big)^2$ and recall that C is defined in Condition1 and $\tilde{C}$ satisfies $\max_{1\leqslant g\leqslant G}\|\boldsymbol{\beta}^{(g)}\|_1\leqslant\tilde{C}$.

Lemma 2. *Assume that the conditions of Theorems 1 and 2 hold. Then with probability at least* $1-1/(pG)$,

$$\|\hat{\boldsymbol{B}}-\boldsymbol{B}\|_F^2\leqslant\frac{9}{2\tilde{c}\epsilon}\lambda_1^2\tilde{w}_{\max}^2 s+\frac{18}{\tilde{c}\epsilon}\lambda_2^2\tilde{v}_{\max}^2\tilde{s}.$$

Proof. Note that Condition 4 and Theorem 1 imply

$$\sqrt{\frac{nG^2}{(sG^3+\tilde{s})\log(p\vee G)}} = O(w_{\min}\wedge v_{\min})$$
$$\tilde{w}_{\max}\wedge\tilde{v}_{\max} > 1/(2\tilde{C}).$$

Combined with the rate conditions of λ_1 and λ_2 in Theorem 2, we also have $w_{\min}\wedge v_{\min} > \tilde{w}_{\max}\vee\tilde{v}_{\max}$, $\sqrt{16G(\log p+\log G)/n} < \lambda_1\tilde{w}_{\max}$, and $\sqrt{64(\log p+\log G)/(nG^2)} < \lambda_2\tilde{v}_{\max}$.

For a minimizer $\hat{\boldsymbol{B}} = (\hat{\beta}_{jg})_{1\leqslant j\leqslant p,1\leqslant g\leqslant G}$ of the original optimization (5) in the main paper, let $\hat{\boldsymbol{\beta}} := \mathrm{vec}(\hat{\boldsymbol{B}}) \in \mathbb{R}^{pG}$, $\boldsymbol{\Delta} := \hat{\boldsymbol{B}} - \boldsymbol{B} \in \mathbb{R}^{p\times G}$, and $\boldsymbol{\delta} := \mathrm{vec}(\boldsymbol{\Delta}) = \hat{\boldsymbol{\beta}} - \boldsymbol{\beta}$. Let $\boldsymbol{\delta}^{(g)} \in \mathbb{R}^p$ be the gth vector in $\boldsymbol{\delta}$, i.e., $\boldsymbol{\delta} = [(\boldsymbol{\delta}^{(1)})^\top,\ldots,(\boldsymbol{\delta}^{(G)})^\top]^\top \in \mathbb{R}^{pG}$. We have

$$\begin{aligned}
&P(\boldsymbol{B},\hat{\boldsymbol{B}})\\
&:= \lambda_1\sum_{j=1}^p w_j\|\boldsymbol{B}_{j,\cdot}\|_2 + \lambda_2\sum_{j=1}^p\sum_{g<g'} v_{j,gg'}|\boldsymbol{B}_{jg}-\boldsymbol{B}_{jg'}|\\
&\quad - \lambda_1\sum_{j=1}^p w_j\|\hat{\boldsymbol{B}}_{j,\cdot}\|_2 - \lambda_2\sum_{j=1}^p\sum_{g<g'} v_{j,gg'}|\hat{\boldsymbol{B}}_{jg}-\hat{\boldsymbol{B}}_{jg'}|\\
&\leqslant \lambda_1\tilde{w}_{\max}\sum_{j\in S}\|\boldsymbol{\Delta}_{j,\cdot}\|_2 - \lambda_1 w_{\min}\sum_{j\in S^c}|\boldsymbol{\Delta}_{j,\cdot}\|_2\\
&\quad + \lambda_2\tilde{v}_{\max}\|(\boldsymbol{A\delta})_\Omega\|_1 - \lambda_2 v_{\min}\|(\boldsymbol{A\delta})_{\Omega^c}\|_1.
\end{aligned}$$

Combining this and the same arguments in the proof of Theorem 1, it holds that with probability at least $1-1/(pG)$,

$$\begin{aligned}
&\frac{\epsilon}{n}\|\mathbf{X}\boldsymbol{\delta}\|_2^2\\
&\leqslant \sqrt{\frac{4G\log(pG)}{n}}\sum_{j=1}^p\|\boldsymbol{\Delta}_{j,\cdot}\|_2 + \sqrt{\frac{16\log(pG)}{nG^2}}\|\boldsymbol{A\delta}\|_1\\
&\quad + P(\boldsymbol{B},\hat{\boldsymbol{B}})\\
&\leqslant \frac{\lambda_1\tilde{w}_{\max}}{2}\sum_{j\in S}\|\boldsymbol{\Delta}_{j,\cdot}\|_2 + \frac{\lambda_1 w_{\min}}{2}\sum_{j\in S^c}\|\boldsymbol{\Delta}_{j,\cdot}\|_2\\
&\quad + \frac{\lambda_2\tilde{v}_{\max}}{2}\|(A\boldsymbol{\delta})_\Omega\|_1 + \frac{\lambda_2 v_{\min}}{2}\|(A\boldsymbol{\delta})_{\Omega^c}\|_1 + P(\boldsymbol{B},\hat{\boldsymbol{B}})\\
&\leqslant \frac{3\lambda_1\tilde{w}_{\max}}{2}\sum_{j\in S}\|\boldsymbol{\Delta}_{j,\cdot}\|_2 - \frac{\lambda_1 w_{\min}}{2}\sum_{j\in S^c}\|\boldsymbol{\Delta}_{j,\cdot}\|_2\\
&\quad + \frac{3\lambda_2\tilde{v}_{\max}}{2}\|(\boldsymbol{A\delta})_\Omega\|_1 - \frac{\lambda_2 v_{\min}}{2}\|(\boldsymbol{A\delta})_{\Omega^c}\|_1,
\end{aligned}\tag{S10}$$

where the second inequality follows from the conditions of λ_1 and λ_2, and the last inequality follows from $w_{\min}\geqslant\tilde{w}_{\max}$. Because $w_{\min}\wedge v_{\min} > \tilde{w}_{\max}\vee\tilde{v}_{\max}$ and $\lambda_2 =$

$G^{-3/2}\lambda_1$, (S10) gives the following inequality:

$$
\begin{aligned}
&G\sqrt{G}\sum_{j\in S^c}\|\boldsymbol{\Delta}_{j,\cdot}\|_2 + \|(\boldsymbol{A}\boldsymbol{\delta})_{\Omega^c}\|_1 \\
&\quad\leqslant 3G\sqrt{G}\sum_{j\in S}\|\boldsymbol{\Delta}_{j,\cdot}\|_2 + 3\|(\boldsymbol{A}\boldsymbol{\delta})_{\Omega}\|_1.
\end{aligned}
$$

Using the restricted strong convexity presented in Condition 2, we obtain

$$
\begin{aligned}
&\tilde{c}\epsilon\|\boldsymbol{\delta}\|_2^2 \\
&\leqslant \frac{\epsilon}{n}\|\mathbf{X}\boldsymbol{\delta}\|_2^2 \\
&\leqslant 1.5\lambda_1\tilde{w}_{\max}\sum_{j\in S}\|\boldsymbol{\Delta}_{j,\cdot}\|_2 + 1.5\lambda_2\tilde{v}_{\max}\|(\boldsymbol{A}\boldsymbol{\delta})_{\Omega}\|_1 \\
&\leqslant 1.5\lambda_1\tilde{w}_{\max}\sqrt{s}\|\boldsymbol{\delta}\|_2 + 1.5\lambda_2\tilde{v}_{\max}\sqrt{\tilde{s}}\|\boldsymbol{A}\boldsymbol{\delta}\|_2 \\
&\leqslant \frac{9}{4\tilde{c}\epsilon}\lambda_1^2\tilde{w}_{\max}^2 s + \frac{1}{4}\tilde{c}\epsilon\|\boldsymbol{\delta}\|_2^2 + \frac{9}{\tilde{c}\epsilon}\lambda_2^2\tilde{v}_{\max}^2\tilde{s} + \frac{1}{4}\tilde{c}\epsilon\|\boldsymbol{\delta}\|_2^2,
\end{aligned}
$$

where the last inequality uses $\|\boldsymbol{A}\boldsymbol{\delta}\|_2 \leqslant 2\|\boldsymbol{\delta}\|_2$ and $2ab \leqslant a^2 + b^2$ for any numbers a and b. This gives

$$
\|\boldsymbol{\delta}\|_2^2 \leqslant \frac{9}{2\tilde{c}\epsilon}\lambda_1^2\tilde{w}_{\max}^2 s + \frac{18}{\tilde{c}\epsilon}\lambda_2^2\tilde{v}_{\max}^2\tilde{s}.
$$

Combining with the definitions of λ_1 and λ_2, this completes the proof. □

For a $s \times G$ matrix $\boldsymbol{\Delta} = (\Delta_{jg})_{1\leqslant j\leqslant s, 1\leqslant g\leqslant G} = [\boldsymbol{\Delta}^{(1)}, \ldots, \boldsymbol{\Delta}^{(g)}]$, let

$$
\bar{L}_n(\boldsymbol{\Delta}) = \frac{1}{n}\sum_{g=1}^{G}\sum_{i=1}^{n_g}\ell(y_i^{(g)}, (\mathbf{x}_{iS}^{(g)})^\top\boldsymbol{\Delta}^{(g)}).
$$

Next, define the oracle penalized estimator using the underlying support set S and Ω:

$$
\begin{aligned}
\hat{\boldsymbol{B}}_S^{ora} &= [\hat{\boldsymbol{\beta}}^{(1),ora}, \ldots, \hat{\boldsymbol{\beta}}^{(G),ora}] \\
&:= \underset{\boldsymbol{\Delta}=[\boldsymbol{\Delta}^{(1)},\ldots,\boldsymbol{\Delta}^{(G)}]\in\mathbb{R}^{s\times G}}{\arg\min} \quad \bar{L}_n(\boldsymbol{\Delta}) + P_\lambda(\boldsymbol{\Delta}), \\
&\quad s.t. \quad \Delta_{jg} = \Delta_{jg'} \text{ for } (j,g,g') \notin \Omega,
\end{aligned}
\tag{S11}
$$

where

$$
P_\lambda(\boldsymbol{\Delta}) := \lambda_1\sum_{j=1}^{s} w_j\|\boldsymbol{\Delta}_{j,\cdot}\|_2 + \lambda_2\sum_{j=1}^{s}\sum_{g<g'} v_{j,gg'}|\Delta_{jg} - \Delta_{jg'}|.
$$

The following Lemma provides an estimation error bound for the oracle estimator $\hat{\boldsymbol{B}}_S^{ora}$.

Lemma 3. *Assume that the conditions of Theorem 2 hold. Then, we have*

$$\max_{1\leqslant g\leqslant G}\|\hat{\boldsymbol{\beta}}^{(g),ora}-\boldsymbol{\beta}_S^{(g)}\|_2=O_p\left(\sqrt{\frac{sG^2\log(sG)}{n}}\right).$$

Proof. Let $\boldsymbol{\beta}_S := \mathrm{vec}(\boldsymbol{B}_{S,\cdot})$, $\hat{\boldsymbol{\beta}}_S^{ora} := \mathrm{vec}(\hat{\boldsymbol{B}}_S^{ora})$, $\boldsymbol{\Delta} := \hat{\boldsymbol{B}}_S^{ora} - \boldsymbol{B}_{S,\cdot}$ and

$$\boldsymbol{\delta} := [(\boldsymbol{\delta}^{(1)})^\top,\ldots,(\boldsymbol{\delta}^{(G)})^\top]^\top := \mathrm{vec}(\Delta) = \hat{\boldsymbol{\beta}}_S^{ora} - \boldsymbol{\beta}_S,$$

i.e., $\boldsymbol{\delta}^{(g)} = \hat{\boldsymbol{\beta}}^{(g),ora} - \boldsymbol{\beta}_S^{(g)}$. Let $[\nabla\bar{L}_n(\boldsymbol{B}_{S,\cdot})]_g \in \mathbb{R}^s$ be the subvector of $\nabla\bar{L}_n(\boldsymbol{B}_{S,\cdot})$ with elements in the index set $\{sg-s+1, sg-s+2,\ldots,sg-1,sg\}$. Let

$$\epsilon := \exp(-2C\tilde{C})/\Big(1+\exp(-2C\tilde{C})\Big)^2.$$

Comparing the objective values at $\hat{\boldsymbol{B}}_S^{ora}$ and

$$\ddot{\boldsymbol{B}} := [\hat{\boldsymbol{\beta}}^{(1),ora},\ldots,\hat{\boldsymbol{\beta}}^{(g-1),ora},\boldsymbol{\beta}_S^{(g)},\hat{\boldsymbol{\beta}}^{(g+1),ora},\ldots,\hat{\boldsymbol{\beta}}^{(G),ora}],$$

and use the same arguments in the proof of Theorem 1, we have for $\ddot{\boldsymbol{\beta}} := \mathrm{mat}(\ddot{\boldsymbol{B}})$,

$$\begin{aligned}
&\frac{\epsilon}{n}\|\mathbf{X}_{\cdot,S}^{(g)}\boldsymbol{\delta}^{(g)}\|_2^2\\
&\leqslant |\langle[\nabla\bar{L}_n(\hat{\boldsymbol{B}}_{S,\cdot}^{ora})]_g,\boldsymbol{\delta}^{(g)}\rangle| + \lambda_1\sum_{j\in S}w_j\|\ddot{\boldsymbol{B}}_{j,\cdot}\|_2\\
&-\lambda_1\sum_{j\in S}w_j\|\hat{\boldsymbol{B}}_{j,\cdot}^{ora}\|_2+\lambda_2\sum_{(j,g,g')\in\Omega}v_{j,gg'}|\ddot{\boldsymbol{B}}_{jg}-\ddot{\boldsymbol{B}}_{jg'}|\\
&\quad-\lambda_2\sum_{(j,g,g')\in\Omega}v_{j,gg'}|\hat{\boldsymbol{B}}_{jg}^{ora}-\hat{\boldsymbol{B}}_{jg'}^{ora}| \qquad\qquad (S12)\\
&\leqslant \max_{j\in S}\Big|[[\nabla\bar{L}_n(\hat{\boldsymbol{B}}_{S,\cdot}^{ora})]_g]_j\Big|\cdot\|\boldsymbol{\delta}^{(g)}\|_1\\
&\quad+\lambda_1\sum_{j\in S}w_j\|\ddot{\boldsymbol{B}}_{j\cdot}-\hat{\boldsymbol{B}}_{j,\cdot}^{ora}\|_2\\
&\quad+\lambda_2\sum_{(j,g,g')\in\Omega}v_{j,gg'}|\hat{\boldsymbol{B}}_{jg}^{ora}-\ddot{\boldsymbol{B}}_{jg}-\hat{\boldsymbol{B}}_{jg'}^{ora}+\ddot{\boldsymbol{B}}_{jg'}|.
\end{aligned}$$

Note that we have

$$\sum_{j\in S} w_j \|\ddot{\boldsymbol{B}}_{j,\cdot} - \hat{\boldsymbol{B}}^{ora}_{j,\cdot}\|_2 = \sum_{j\in S} w_j |\boldsymbol{\delta}^{(g)}_j| \leqslant \tilde{w}_{\max}\|\boldsymbol{\delta}^{(g)}\|_1,$$

$$\begin{aligned}
&\sum_{(j,g,g')\in\Omega} v_{j,gg'}|\hat{\boldsymbol{B}}^{ora}_{jg} - \ddot{\boldsymbol{B}}_{jg} - \hat{\boldsymbol{B}}^{ora}_{jg'} + \ddot{\boldsymbol{B}}_{jg'}| \\
&\quad\leqslant 2\sum_{(j,g,g')\in\Omega} v_{j,gg'}|\hat{\boldsymbol{B}}^{ora}_{jg} - \ddot{\boldsymbol{B}}_{jg}| \\
&\quad\leqslant 2\sum_{(j,g,g')\in\Omega} v_{j,gg'}|\delta^{(g)}_j| \\
&\quad\leqslant 2\left(\sum_{(j,g,g')\in\Omega} v^2_{j,gg'}\right)^{1/2}\|\boldsymbol{\delta}^{(g)}\|_2 \\
&\quad\leqslant 2\sqrt{\tilde{s}}\tilde{v}_{\max}\|\boldsymbol{\delta}^{(g)}\|_2.
\end{aligned} \tag{S13}$$

Combining (S12)-(S13), and the fact that

$$\max_{j\in S,\ 1\leqslant g\leqslant G} |[[\nabla \bar{L}_n(\hat{\boldsymbol{B}}^{ora}_{S,\cdot})]_g]_j| \leqslant \sqrt{16\log(sG)/n}$$

holds with probability at least $1 - 1/(sG)$, as in the proof of Lemma 1, we have for all $1 \leqslant g \leqslant G$,

$$\begin{aligned}
&\frac{\epsilon}{n}\|\mathbf{X}^{(g)}_{\cdot,S}\boldsymbol{\delta}^{(g)}\|_2^2 \\
&\leqslant \sqrt{\frac{16\log(sG)}{n}}\|\boldsymbol{\delta}^{(g)}\|_1 + \lambda_1\tilde{w}_{\max}\|\boldsymbol{\delta}^{(g)}\|_1 \\
&\qquad + \lambda_2\sqrt{\tilde{s}}\tilde{v}_{\max}\|\boldsymbol{\delta}^{(g)}\|_2 \\
&\leqslant \sqrt{\frac{16s\log(sG)}{n}}\|\boldsymbol{\delta}^{(g)}\|_2 + \lambda_1\tilde{w}_{\max}\sqrt{s}\|\boldsymbol{\delta}^{(g)}\|_2 \\
&\qquad + \lambda_2\sqrt{\tilde{s}}\tilde{v}_{\max}\|\boldsymbol{\delta}^{(g)}\|_2 \\
&\leqslant \frac{6s\log(sG)}{nc_{\min}} + \frac{3s\lambda_1^2\tilde{w}^2_{\max}}{2c_{\min}} + \frac{3\tilde{s}\lambda_2^2\tilde{v}^2_{\max}}{2c_{\min}} + \frac{c_{\min}\|\boldsymbol{\delta}^{(g)}\|_2^2}{2}.
\end{aligned}$$

Applying Conditions 1 and 5, we get

$$\begin{aligned}
&\epsilon c_{\min}\|\boldsymbol{\delta}^{(g)}\|_2^2 \\
&\leqslant \frac{G}{C_2}\Big(\sqrt{\frac{16s\log(sG)}{n}} + \lambda_1\tilde{w}_{\max}\sqrt{s} + \lambda_2\sqrt{\tilde{s}}\tilde{v}_{\max}\Big)\|\boldsymbol{\delta}^{(g)}\|_2 \\
&\leqslant \frac{\epsilon c_{\min}}{2}\|\boldsymbol{\delta}(g)\|_2^2 + \frac{24sG^2\log(sG)}{n\epsilon c_{\min}C_2^2} + \frac{3\lambda_1^2\tilde{w}^2_{\max}sG^2}{2\epsilon c_{\min}C_2^2} \\
&\quad + \frac{3\lambda_2^2\tilde{v}^2_{\max}\tilde{s}G^2}{2\epsilon c_{\min}C_2^2},
\end{aligned}$$

where we use the fact that $2ab \leqslant a^2 + b^2$ for any numbers a and b. Combining with Condition 1, we obtain

$$\max_{1\leqslant g\leqslant G} \|\boldsymbol{\delta}^{(g)}\|_2^2 \leqslant \frac{48sG^2\log(sG)}{n\epsilon^2 c_{\min}^2 C_2^2} + \frac{3\lambda_1^2 \tilde{w}_{\max}^2 sG^2}{\epsilon^2 c_{\min}^2 C_2^2} + \frac{3\lambda_2^2 \tilde{v}_{\max}^2 \tilde{s} G^2}{\epsilon^2 c_{\min}^2 C_2^2}.$$

This completes the proof. □

S3 Technical conditions

In this section, we provide the technical conditions required to prove Theorems 1-2. Specifically, Subsection S3.1 discusses the technical conditions required to establish Theorems 1-2, while Subsection S3.2 introduces an additional technical condition that is crucial to prove Theorems 3-5.

S3.1 Technical conditions for Theorems 1 and 2

Condition 1. *There exist positive constants* $c_{\min}, c_{\max}, C$ *such that* $\max_{g\in\{1,\ldots,G\}} \|\mathbf{X}^{(g)}\|_{\max} \leqslant C$ *and for* $g = 1, \ldots, G$,

$$c_{\min} \leqslant \lambda_{\min}\left(\frac{[\mathbf{X}_{\cdot,S}^{(g)}]^\top \mathbf{X}_{\cdot,S}^{(g)}}{n_g}\right) \leqslant \lambda_{\max}\left(\frac{[\mathbf{X}_{\cdot,S}^{(g)}]^\top \mathbf{X}_{\cdot,S}^{(g)}}{n_g}\right) \leqslant c_{\max}.$$

Condition 2. *For the restricted set*

$$A = \left\{\boldsymbol{\Delta} \in \mathbb{R}^{p\times G} :\ G\sqrt{G}\sum_{j\in S^c} \|\boldsymbol{\Delta}_{j,\cdot}\|_2 + \sum_{(j,g,g')\in\Omega^c} |\Delta_{jg} - \Delta_{jg'}| \leqslant 3G\sqrt{G}\sum_{j\in S} \|\boldsymbol{\Delta}_{j,\cdot}\|_2 + 3\sum_{(j,g,g')\in\Omega} |\Delta_{jg} - \Delta_{jg'}|\right\}$$

the following restricted strong convexity holds:

$$\inf_{\Delta\in A, \Delta\neq 0} \frac{\|\mathbf{X} vec(\boldsymbol{\Delta})\|_2^2}{n\|\boldsymbol{\Delta}\|_F^2} > \tilde{c} \quad \textit{for some} \quad \tilde{c} > 0.$$

Condition 3. *For* $j \in S^c$ *and* $1 \leqslant g \leqslant G$, *it holds that* $\sup_{\boldsymbol{\delta}\in\mathbb{R}^s} s\sum_{i=1}^{n_g} \left\{x_{ij}^{(g)} \mathbf{x}_{iS}^{(g)} \boldsymbol{\delta}\right\}^2 / (n\|\boldsymbol{\delta}\|_2^2) = O(1)$.

Condition 4. *The true coefficient matrix* $\boldsymbol{B}$ *satisfies*

$$\min_{j\in S} \|\boldsymbol{\beta}_{(j)}\|_2 \wedge \min_{(j,g,g')\in\Omega} |\beta_j^{(g)} - \beta_j^{(g')}| \gg \sqrt{\frac{G^2 s(sG^3 + \tilde{s})\log(p\vee G)}{n}}.$$

Condition 1 assumes uniform boundness and minimum and maximum eigenvalue condition on the true support set of $\mathbf{X}^{(g)}$, which is commonly assumed in the literature [4, 5, 6, 7]. Condition 2 means that $\mathbf{X}$ should satisfy the Restricted Strong Convexity (RSC) condition over a restricted set A. Note that Condition 2 is different from the restricted strong convexity condition in existing high-dimensional regression models that use decomposable regularizers [8]. Our regularizer is the sum of a group Lasso type and a fused Lasso type, which is not decomposable. Condition 3 is also imposed in Zheng et al. [5] with a slight modification to prove the oracle property of their estimator, which restricts the correlations between relevant covariates and irrelevant covariates. Condition 4 constrains the minimum signal strength, which is commonly assumed in the literature for variable selection [5, 7, 9, 10].

S3.2 An additional technical condition for Theorems 3-5

We impose an additional assumption to analyze theoretically our debiased estimator $\hat{\boldsymbol{b}}^{(g)}$.

Condition 5. *There exist an absolute constant* $0 < C_2 \leqslant 1$ *such that*

$$\frac{C_2 n}{G} \leqslant \min_{1\leqslant g\leqslant G} n_g$$

$$s^2 G^2 \log p \cdot \log(s \vee G) = o(n)$$

$$\max_{1\leqslant g\leqslant G} |||\boldsymbol{M}^{(g)}|||_1 \leqslant s_0 = o\Big(\frac{n}{s^2 G^2 \log(sG)\ \sqrt{\max_g n_g}}\Big)$$

$$\log p = o\big(\min_{1\leqslant g\leqslant G} n_g\big).$$

Condition 5 imposes constraints on the sparsity parameter s, the number of groups G, the number of covariates p, the sample sizes n_g's, and the norm of $\boldsymbol{M}^{(g)}$. In particular, the condition $\max_{1\leqslant g\leqslant G} |||\boldsymbol{M}^{(g)}|||_1 \leqslant s_0$ is crucial to show the theoretical properties of our debiased estimator. The similar condition is also imposed in Javanmard and Montanari [11] in the linear regression setting.

S4 Additional numerical results

In this section we present additional numerical results. Subsection S4.1 reports the results of sensitivity analyses of the constraint parameter in quadratic programming. Subsection S4.2 compares the proposed method with DL and DL-E, which are Lasso-based bias correction methods, in terms of bias, standard error, and coverage probability for individual coefficients. Subsection S4.3 provides additional simulation results using the simulation model introduced in Section 4.1, focusing specifically on a case where the sample sizes for the groups differ. Subsection S4.4 provides the results of a sensitivity analysis of the results of the CCLE data analysis using DFGL based on parametric bootstrapping.

S4.1 Sensitivity analysis of constraint parameter

In the proposed DFGL, standard errors are obtained via the quadratic programming. We conduct sensitivity analyses of the constraint parameter μ_g in the quadratic programming using a simulation model described in Section 4.1. Specifically, we consider the first-order autoregressive correlation structure for the correlation structure of the covariates. We set $\mu_g = c\sqrt{\log p/n_g}$ for $g = 1, \ldots, G$, where c is set as $c \in \{0.1, 0.5, 0.7, 1.0, 1.5, 2.0, 3.0\}$. The sensitivity of the constraint parameter μ_g is assessed based on type I error and power. Tables S1 and S2 record results when testing the homogeneity and significance, respectively. In summary, large c generally provides high power and type I error. Thus, we recommend using $c = 0.7$ or $c = 1.0$, which balances the type I error and power.

Table S1: Probability of rejection of $H_0 : \beta_j^{(1)} = \ldots = \beta_j^{(G)}$ at $\alpha = 0.05$ in the sensitivity analysis of μ_g, where μ_g is set as $\mu_g = c\sqrt{\log p/n_g}$ for $g = 1, \ldots, G$, and $n_1, \ldots, n_G$ are set as $n_1 = \ldots = n_G = m$.

(m, p)	j	$\min_g \beta_j^{(g)}$	$\max_g \beta_j^{(g)}$	c						
				0.1	0.5	0.7	1.0	1.5	2.0	3.0
Power										
(200, 80)	1	−0.6	0.6	0.64	0.92	0.93	0.96	0.96	0.95	0.94
	2	−0.6	0.6	0.60	0.83	0.87	0.86	0.83	0.76	0.75
	3	−0.4	0.6	0.30	0.59	0.62	0.65	0.65	0.56	0.57
	4	−0.4	0.6	0.36	0.63	0.76	0.80	0.85	0.85	0.84
(300, 120)	1	−0.50	0.50	0.95	1.00	1.00	1.00	1.00	1.00	1.00
	2	−0.50	0.50	0.79	0.96	0.99	0.99	1.00	1.00	1.00
	3	−0.35	0.50	0.50	0.89	0.91	0.95	0.95	0.95	0.93
	4	−0.35	0.50	0.54	0.90	0.92	0.95	0.96	0.98	0.98
Type I error										
(200, 80)	5	0.4	0.4	0.00	0.01	0.01	0.04	0.05	0.07	0.07
	8	−0.4	−0.4	0.02	0.02	0.01	0.01	0.02	0.02	0.02
	9	1.5	1.5	0.01	0.00	0.02	0.04	0.05	0.07	0.11
	10	1.5	1.5	0.00	0.02	0.05	0.06	0.07	0.08	0.08
	11	0.0	0.0	0.04	0.05	0.04	0.05	0.04	0.04	0.04
	12	2.5	2.5	0.01	0.05	0.04	0.05	0.08	0.07	0.06
(300, 120)	5	0.4	0.4	0.00	0.02	0.03	0.02	0.03	0.05	0.09
	8	−0.4	−0.4	0.00	0.01	0.01	0.01	0.02	0.03	0.04
	9	1.5	1.5	0.03	0.03	0.04	0.03	0.03	0.03	0.04
	10	1.5	1.5	0.02	0.01	0.02	0.01	0.03	0.01	0.02
	11	0.0	0.0	0.01	0.02	0.02	0.02	0.02	0.02	0.02
	12	2.5	2.5	0.01	0.00	0.00	0.00	0.02	0.04	0.04

Table S2: Probability of rejection of $H_0 : \beta_j^{(1)} = \ldots = \beta_j^{(G)} = 0$ at $\alpha = 0.05$ in the sensitivity analysis of μ_g, where μ_g is set as $\mu_g = c\sqrt{\log p/n_g}$ for $g = 1, \ldots, G$, and $n_1, \ldots, n_G$ are set as $n_1 = \ldots = n_G = m$.

(m, p)	j	$\min_g \beta_j^{(g)}$	$\max_g \beta_j^{(g)}$	c						
				0.1	0.5	0.7	1.0	1.5	2.0	3.0
Power										
$(200, 80)$	1	-0.6	0.6	0.78	0.99	0.99	1.00	1.00	1.00	1.00
	2	-0.6	0.6	0.73	0.91	0.95	0.97	0.98	0.98	0.98
	3	-0.4	0.6	0.51	0.84	0.88	0.92	0.93	0.94	0.91
	4	-0.4	0.6	0.58	0.88	0.91	0.97	0.98	0.98	0.98
	5	0.4	0.4	0.30	0.58	0.67	0.73	0.82	0.84	0.86
	8	-0.4	-0.4	0.20	0.38	0.45	0.50	0.49	0.44	0.44
	9	1.5	1.5	1.00	1.00	1.00	1.00	1.00	1.00	1.00
	10	1.5	1.5	1.00	1.00	1.00	1.00	1.00	1.00	1.00
$(300, 120)$	1	-0.6	0.6	0.97	1.00	1.00	1.00	1.00	1.00	1.00
	2	-0.6	0.6	0.92	1.00	1.00	1.00	1.00	1.00	1.00
	3	-0.4	0.6	0.82	0.99	1.00	1.00	1.00	1.00	1.00
	4	-0.4	0.6	0.79	0.97	0.99	1.00	1.00	1.00	1.00
	5	0.4	0.4	0.52	0.80	0.87	0.92	0.97	0.98	1.00
	8	-0.4	-0.4	0.41	0.78	0.88	0.92	0.94	0.97	0.99
	9	1.5	1.5	1.00	1.00	1.00	1.00	1.00	1.00	1.00
	10	1.5	1.5	1.00	1.00	1.00	1.00	1.00	1.00	1.00
Type I error										
$(200, 80)$	6	0.0	0.0	0.02	0.00	0.01	0.03	0.06	0.07	0.08
	7	0.0	0.0	0.00	0.02	0.03	0.02	0.02	0.04	0.05
	11	0.0	0.0	0.01	0.03	0.04	0.05	0.05	0.05	0.05
$(300, 120)$	6	0.0	0.0	0.02	0.02	0.02	0.03	0.03	0.04	0.07
	7	0.0	0.0	0.00	0.00	0.01	0.01	0.01	0.02	0.03
	11	0.0	0.0	0.00	0.00	0.02	0.03	0.11	0.14	0.14

S4.2 Bias, standard error, and coverage probability

In this subsection, we examine the bias, standard error, and coverage probability of the proposed method and Lasso-based methods when considering individual coefficients. Tables S3 and S4 summarize the bias, standard error, and coverage probability, respectively, under the simulation models described in Section 4.1. DL, a debiased Lasso based on node-wise regression, generally produces large biases compared to other methods. DL-E, the approach based on the exact inverse of the information matrix, provides large standard errors, so it has relatively small biases and desired coverage probabilities compared to other methods. Although the proposed DFGL has relatively smaller standard errors compared to DL-E, the two methods provide similar biases when the covariates

of interest have relatively weak signals. Therefore, the proposed DFGL has more power than DL and DL-E when testing the overall significance of the coefficients for a covariate with weak signals. Furthermore, although DL-E has large standard errors, DL-E fails to control for Type I errors when testing the homogeneity of the coefficients for a covariate with strong signals. In contrast to DL-E, the proposed DFGL gives type I errors close to or less than the nominal level in these cases.

S4.3 An additional simulation study for unbalanced designs

We perform an additional simulation to evaluate the performance of DFGL under the simulation models in Section 4.1 by considering different sample sizes across groups. We consider model parameters as follows: $p = 120$, $G = 7$, $n_1 = \ldots = n_4 = 200$, but $n_5 = n_6 = n_7 = 300$.

S4.3.1 Testing homogeneity and significance

Tests of homogeneity and significance were performed at $\alpha = 0.05$, as described in Section 4.2. Table S7 summarizes the powers for testing homogeneity and significance, while Table S8 summarizes the type I errors. Overall, the DFGL method has higher powers compared to the other methods, while keeping the type I errors close to the nominal level, except in the cases of testing the homogeneity of the effects of the covariate with the strongest signal. DL, which has low power compared to DFGL, has higher type I errors compared to DFGL in these cases. Although the four methods, DL-B, DL-E, DL-E-B, and DR-B, have type I errors below the nominal level, these methods are conservative.

S4.3.2 Multiple testing

We consider the following two multiple testing problems (S14) and (S15).

$$H_{0,j} : \beta_j^{(1)} = \ldots = \beta_j^{(G)} \text{ vs } H_1 : \text{not } H_{0,j}, \quad j = 1, \ldots, p \tag{S14}$$

$$H_{0,j} : \beta_j^{(1)} = \ldots = \beta_j^{(G)} = 0 \text{ vs } H_1 : \text{not } H_{0,j}, \quad j = 1, \ldots, p \tag{S15}$$

In this analysis, methods with Bonferroni correction, i.e. DL-B, DL-E-B and DR-B, are excluded. This is because they are conservative, as observed in Table S7. Table S9 summarizes the results when p-values are adjusted by the BH procedure to control the familywise error rate. Compared to the results when $n_1 = \ldots = n_G = 300$, the power tends to decrease for all methods. However, the DFGL still has the highest power in all cases, while providing FWER below the nominal level $\alpha = 0.05$. Similar to the results when $n_1 = \ldots = n_G = 300$, DL fails to control FWER when considering the homogeneity test (S14) and DL-E shows low power compared to the two methods, DFGL and DL.

S4.4 Sensitiviy analysis of the results of CCLE data analysis

To investigate the sensitivity of the results of CCLE data analysis using DFGL, we consider parametric bootstrapping. For each drug, we generate response variables from the logistic regression model with coefficients as the FGL estimate obtained from the CCLE data, while conditioning the design matrix. We simulate a total of $B = 300$ bootstrap samples.

For gene-drug pairs previously identified by applying our significance test to the CCLE data, Figure S1 shows the frequency of identification of each of these pairs at the 0.05 significance level over $B = 300$ replicates. We observe that some gene-drug pairs are identified relatively consistently. For example, SLFN11 is identified as a significant gene for Irinotecan and Topotecan in over 45% of the total 300 replicates, and NQO1 is identified as a significant gene for 17-AAG in about 40% of the total 300 replicates. However, some of the gene-drug pairs identified using whole cell lines in Section 5.2 are not identified in most of the bootstrap samples. This may be because we allow for type I errors by setting the significance level to $\alpha = 0.05$; thus, DFGL may identify relatively less significant gene-drug pairs. For the gene-drug pairs that were not identified by applying our significance test to the CCLE data, each gene is identified on average in approximately 6%, 7%, 10%, 6%, and 6% of the $B = 300$ bootstrap samples for 17-AAG, AZD6244, Irinotecan, PD-0325901, and topotecan, respectively. These results suggest that, on average, these gene-drug pairs may be less significant than relatively frequently identified gene-drug pairs, including SLFN11-Topotecan SLFN11-Irinotecan, NQO1-17AAG, SIRPA-AZD6244, and ETV4-PD-0325901. The related results to the significance for SLFN11-Topotecan, SLFN11-Irinotecan, NQO1-17AAG, SIRPA-AZD6244, and ETV4-PD-0325901 were also observed in previous literature [12, 13, 14, 15].

When considering the homogeneity test, the gene SLFN11 is identified relatively frequently for Topotecan compared to other gene-drug pairs, as shown in Figure S2. The application of a penalized mixture regression to the CCLE data [13] also suggests the heterogeneous effects of SLFN11 on Topotecan across clusters.

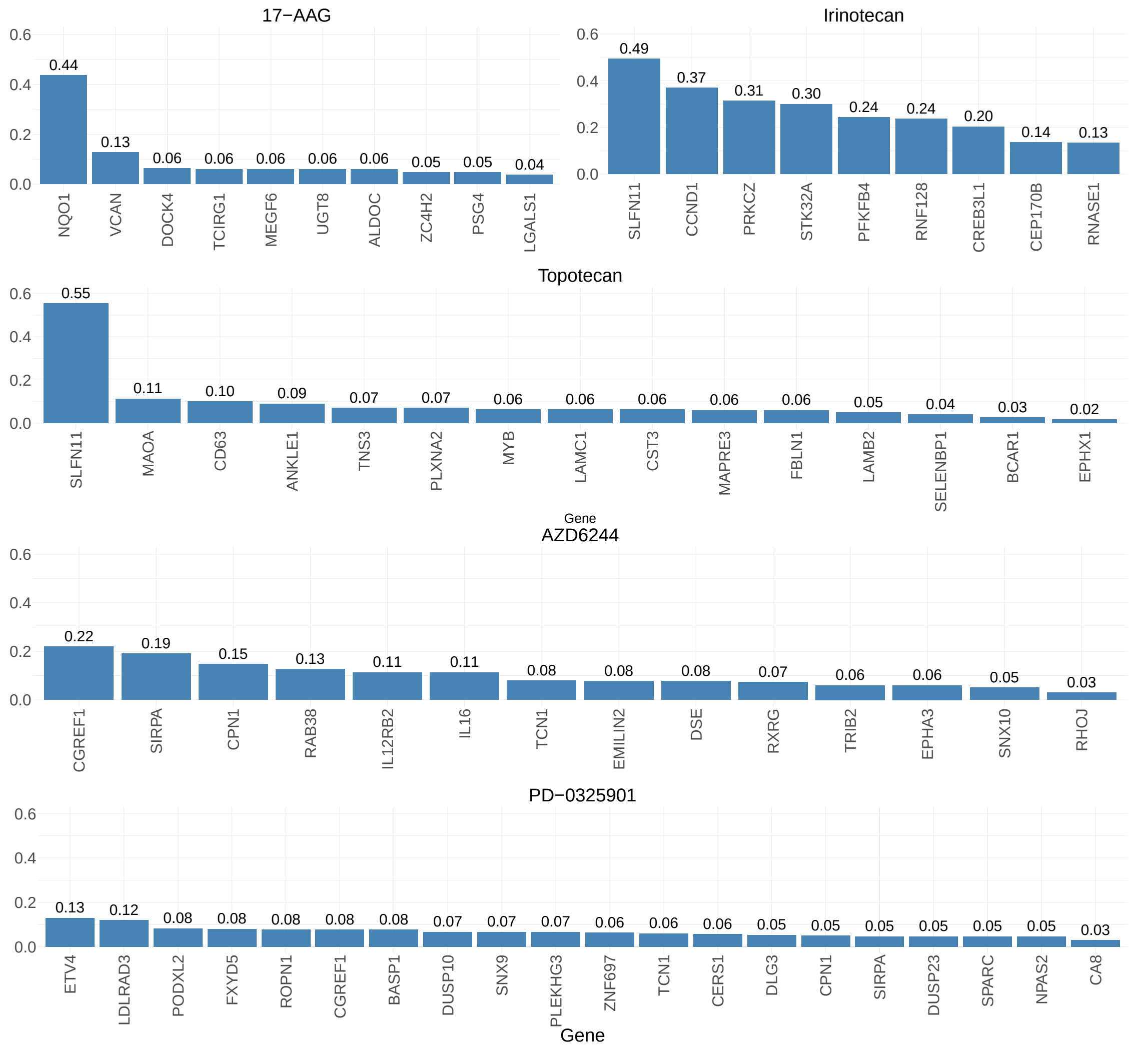

Figure S1: Proportion of rejection of the null hypothesis when testing significance. The figure was created using the ggplot2 package [16] (version 3.4.4; https://cran.r-project.org/web/packages/ggplot2/index.html) in R software [17] (version 4.3.1 for Windows; https://cran.r-project.org/bin/windows/base/old/).

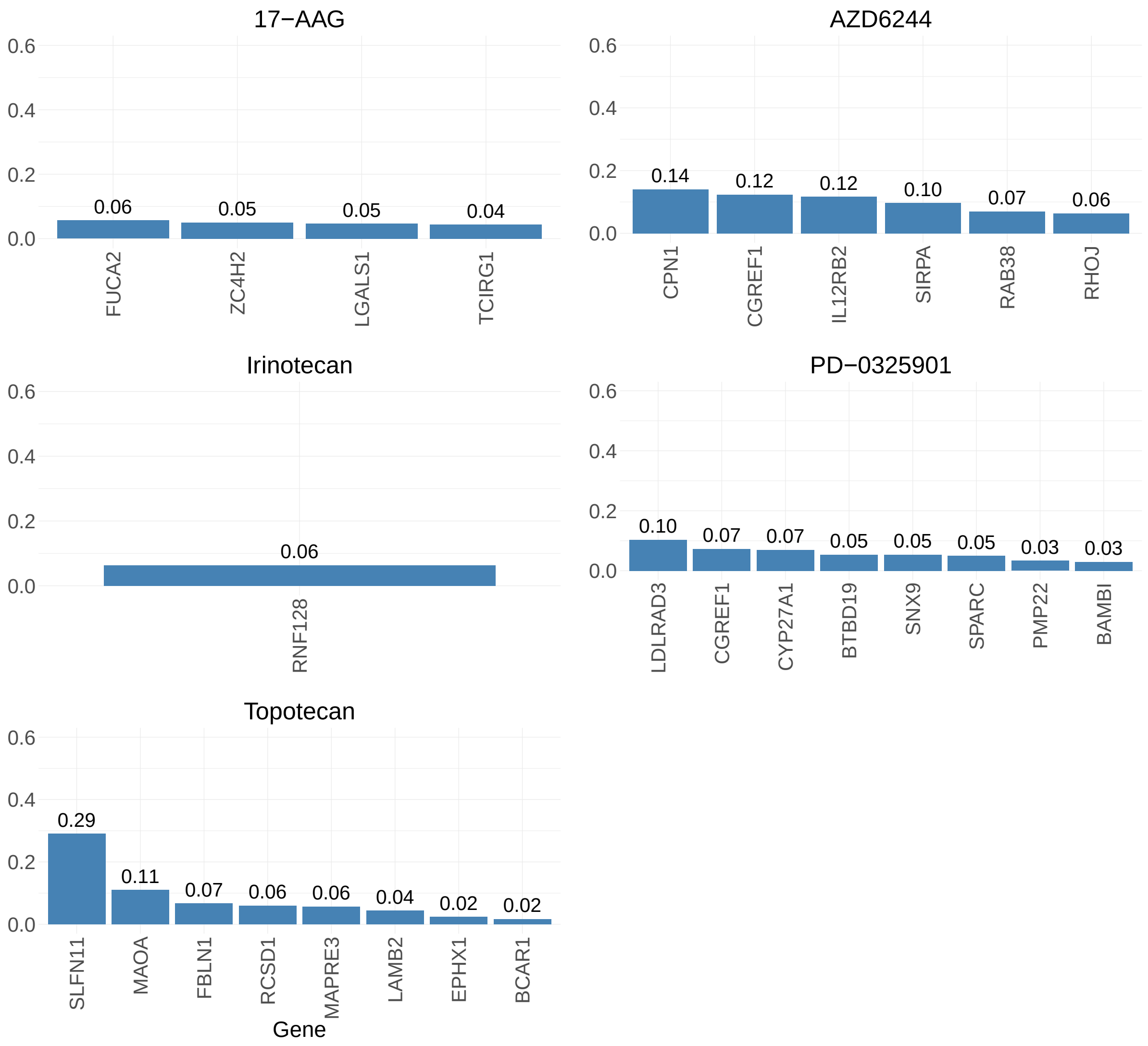

Figure S2: Proportion of rejection of the null hypothesis when testing homogeneity. The figure was created using the ggplot2 package [16] (version 3.4.4; https://cran.r-project.org/web/packages/ggplot2/index.html) in R software [17] (version 4.3.1 for Windows; https://cran.r-project.org/bin/windows/base/old/).

S5 Additional tables

In this section, we include additional tables.

(m, p)	Measure	Methods	$\beta_j^{(g)}$						
			-0.6	-0.4	0.0	0.4	0.6	1.5	2.5
$(200, 80)$	Bias	DFGL	0.21(0.03)	0.19(0.01)	0.00(0.03)	-0.09(0.04)	-0.13(0.04)	-0.21(0.03)	-0.30(0.03)
		DL	0.28(0.04)	0.25(0.01)	0.04(0.07)	-0.11(0.04)	-0.17(0.05)	-0.33(0.04)	-0.60(0.03)
		DL-E	0.19(0.02)	0.18(0.01)	0.00(0.03)	-0.08(0.05)	-0.10(0.06)	-0.11(0.04)	-0.13(0.04)
	S.E	DFGL	0.23(0.02)	0.24(0.02)	0.24(0.02)	0.24(0.02)	0.24(0.02)	0.27(0.03)	0.31(0.03)
		DL	0.23(0.03)	0.24(0.03)	0.24(0.03)	0.24(0.03)	0.24(0.03)	0.26(0.03)	0.28(0.04)
		DL-E	0.38(0.08)	0.40(0.08)	0.40(0.08)	0.40(0.08)	0.39(0.08)	0.42(0.09)	0.46(0.01)
	C.P	DFGL	0.84(0.01)	0.90(0.03)	0.97(0.02)	0.95(0.03)	0.92(0.03)	0.86(0.04)	0.81(0.05)
		DL	0.78(0.04)	0.87(0.04)	0.96(0.03)	0.95(0.03)	0.90(0.03)	0.64(0.06)	0.36(0.02)
		DL-E	0.97 (0.01)	0.98(0.01)	0.99(0.01)	0.99(0.01)	0.96(0.02)	0.91(0.03)	0.85(0.04)
$(300, 120)$	Bias	DFGL	0.18(0.03)	0.16(0.02)	0.01(0.03)	-0.08(0.03)	-0.13(0.03)	-0.22(0.03)	-0.34(0.02)
		DL	0.27(0.04)	0.23(0.02)	0.03(0.05)	-0.11(0.03)	-0.18(0.03)	-0.36(0.05)	-0.64(0.03)
		DL-E	0.19(0.01)	0.17(0.00)	0.01(0.03)	-0.08(0.04)	-0.10(0.04)	-0.12(0.04)	-0.16(0.04)
	S.E	DFGL	0.19(0.01)	0.20(0.01)	0.20(0.01)	0.20(0.01)	0.19(0.01)	0.22(0.01)	0.25(0.01)
		DL	0.19(0.02)	0.20(0.02)	0.20(0.02)	0.20(0.02)	0.19(0.02)	0.21(0.02)	0.23(0.02)
		DL-E	0.31(0.04)	0.32(0.04)	0.32(0.04)	0.32(0.04)	0.32(0.04)	0.34(0.05)	0.37(0.06)
	C.P	DFGL	0.82(0.04)	0.88(0.03)	0.97(0.02)	0.94(0.02)	0.89(0.04)	0.84(0.06)	0.78(0.05)
		DL	0.76(0.07)	0.84(0.06)	0.96(0.03)	0.94(0.03)	0.86(0.05)	0.56(0.09)	0.23(0.04)
		DL-E	0.95(0.02)	0.96(0.02)	0.99(0.01)	0.97(0.01)	0.95(0.03)	0.90(0.03)	0.83(0.05)

Table S3: Bias, standard error, and coverage probability of the proposed method and Lasso-based methods when the covariance structure for the covariates is AR(1). S.E and C.P respectively represent standard error and coverage probability. We consider $j = 6, 7, 11, 15, 20$ to measure performances for zero coefficients. Here, the numbers in parentheses represent standard deviations.

(m,p)	Measure	Methods	$\beta_j^{(g)}$						
			-0.6	-0.4	0.0	0.4	0.6	1.5	2.5
$(200,80)$	Bias	DFGL	0.19(0.03)	0.16(0.03)	0.01(0.03)	-0.08(0.01)	-0.13(0.04)	-0.18(0.03)	-0.28(0.05)
		DL	0.30(0.05)	0.22(0.03)	0.04(0.08)	-0.15(0.01)	-0.19(0.04)	-0.33(0.04)	-0.63(0.06)
		DL-E	0.18(0.04)	0.15(0.01)	0.03(0.04)	-0.10(0.02)	-0.11(0.05)	-0.10(0.06)	-0.13(0.08)
	S.E	DFGL	0.23(0.02)	0.23(0.02)	0.24(0.02)	0.22(0.04)	0.23(0.04)	0.27(0.03)	0.30(0.03)
		DL	0.23(0.02)	0.23(0.03)	0.24(0.03)	0.23(0.02)	0.23(0.03)	0.26(0.03)	0.27(0.04)
		DL-E	0.38(0.08)	0.37(0.07)	0.39(0.08)	0.36(0.07)	0.38(0.08)	0.41(0.09)	0.43(0.10)
	C.P	DFGL	0.84(0.04)	0.90(0.03)	0.96(0.02)	0.93(0.02)	0.90(0.04)	0.87(0.03)	0.82(0.03)
		DL	0.77(0.06)	0.88(0.05)	0.96(0.04)	0.91(0.03)	0.88(0.04)	0.64(0.05)	0.34(0.09)
		DL-E	0.96(0.03)	0.97(0.03)	0.99(0.01)	0.98(0.02)	0.96(0.02)	0.90(0.03)	0.81(0.04)
$(300,120)$	Bias	DFGL	0.18(0.01)	0.14(0.02)	0.01(0.03)	-0.09(0.01)	-0.13(0.03)	-0.19(0.03)	-0.32(0.02)
		DL	0.29(0.03)	0.21(0.02)	0.02(0.06)	-0.14(0.01)	-0.19(0.03)	-0.33(0.03)	-0.63(0.02)
		DL-E	0.19(0.02)	0.15(0.01)	0.00(0.03)	-0.10(0.02)	-0.12(0.05)	-0.10(0.04)	-0.16(0.05)
	S.E	DFGL	0.19(0.01)	0.19(0.01)	0.20(0.01)	0.18(0.01)	0.19(0.01)	0.22(0.02)	0.24(0.01)
		DL	0.19(0.02)	0.19(0.02)	0.19(0.02)	0.18(0.02)	0.19(0.02)	0.21(0.02)	0.22(0.02)
		DL-E	0.31(0.05)	0.30(0.05)	0.32(0.05)	0.29(0.04)	0.31(0.05)	0.33(0.05)	0.35(0.06)
	C.P	DFGL	0.85(0.02)	0.88(0.05)	0.97(0.02)	0.92(0.02)	0.90(0.03)	0.87(0.02)	0.75(0.04)
		DL	0.68(0.04)	0.83(0.07)	0.97(0.03)	0.90(0.02)	0.84(0.05)	0.58(0.05)	0.22(0.04)
		DL-E	0.95(0.02)	0.96(0.02)	0.99(0.01)	0.97(0.01)	0.95(0.03)	0.90(0.03)	0.83(0.05)

Table S4: Bias, standard error, and coverage probability of the proposed method and Lasso-based methods when the covariance structure for the covariates is the block diagonal. S.E and C.P respectively represent standard error and coverage probability. We consider $j = 6, 7, 11, 15, 20$ to measure performances for zero coefficients. Here, the numbers in parentheses represent standard deviations.

(m,p)	j	$\min_g \beta_j^{(g)}$	$\max_g \beta_j^{(g)}$	Methods					
				DFGL	DL	DL-E	DR-B	DL-B	DL-E-B
AR(1)									
$(200,80)$	1	-0.6	0.6	0.99	0.94	0.71	0.00	0.00	0.00
	2	-0.6	0.6	0.95	0.92	0.44	0.00	0.00	0.00
	3	-0.4	0.6	0.88	0.80	0.43	0.00	0.00	0.00
	4	-0.4	0.6	0.91	0.86	0.46	0.00	0.00	0.00
	5	0.4	0.4	0.67	0.54	0.15	0.00	0.00	0.00
	8	-0.4	-0.4	0.45	0.14	0.04	0.00	0.00	0.00
	9	1.5	1.5	1.00	1.00	1.00	0.17	0.73	0.13
	10	1.5	1.5	1.00	1.00	1.00	0.26	0.78	0.18
	12	2.5	2.5	1.00	1.00	1.00	0.99	1.00	0.99
$(300,120)$	1	-0.6	0.6	1.00	1.00	0.89	0.00	0.00	0.00
	2	-0.6	0.6	1.00	0.96	0.80	0.00	0.00	0.00
	3	-0.4	0.6	0.99	0.99	0.72	0.00	0.00	0.00
	4	-0.4	0.6	0.97	0.96	0.79	0.00	0.00	0.00
	5	0.4	0.4	0.88	0.82	0.34	0.00	0.00	0.00
	8	-0.4	-0.4	0.65	0.27	0.09	0.00	0.00	0.00
	9	1.5	1.5	1.00	1.00	1.00	0.62	0.97	0.58
	10	1.5	1.5	1.00	1.00	1.00	0.76	0.98	0.66
	12	2.5	2.5	1.00	1.00	1.00	1.00	1.00	1.00
Block									
$(200,80)$	1	-0.6	0.6	0.97	0.94	0.65	0.00	0.00	0.00
	2	-0.6	0.6	0.90	0.83	0.49	0.00	0.00	0.00
	3	-0.4	0.6	0.93	0.85	0.40	0.00	0.00	0.00
	4	-0.4	0.6	0.95	0.93	0.55	0.00	0.00	0.00
	5	0.4	0.4	0.75	0.47	0.20	0.00	0.00	0.00
	8	-0.4	-0.4	0.63	0.36	0.12	0.00	0.00	0.00
	9	1.5	1.5	1.00	1.00	1.00	0.42	0.90	0.36
	10	1.5	1.5	1.00	1.00	1.00	0.17	0.80	0.10
	12	2.5	2.5	1.00	1.00	1.00	1.00	1.00	1.00
$(300,120)$	1	-0.6	0.6	1.00	0.98	0.86	0.00	0.00	000
	2	-0.6	0.6	1.00	0.99	0.71	0.00	0.00	0.00
	3	-0.4	0.6	0.99	0.98	0.66	0.00	0.00	0.00
	4	-0.4	0.6	0.99	0.99	0.87	0.00	0.00	0.00
	5	0.4	0.4	0.90	0.78	0.28	0.00	0.00	0.00
	8	-0.4	-0.4	0.92	0.78	0.42	0.00	0.00	0.00
	9	1.5	1.5	1.00	1.00	1.00	0.62	1.00	0.90
	10	1.5	1.5	1.00	1.00	1.00	0.76	0.99	0.63
	12	2.5	2.5	1.00	1.00	1.00	1.00	1.00	1.00

Table S5: Power for testing $H_0 : \beta_j^{(1)} = \ldots = \beta_j^{(G)} = 0$ vs H_1 : not H_0 at $\alpha = 0.05$, where $n_1, \ldots, n_G$ are set as $n_1 = \cdots = n_G = m$.

(m,p)	j	$\min_g \beta_j^{(g)}$	$\max_g \beta_j^{(g)}$	Methods					
				DFGL	DL	DL-E	DR-B	DL-B	DL-E-B
AR(1)									
$(200,80)$	6	0.0	0.0	0.01	0.01	0.00	0.00	0.00	0.00
	7	0.0	0.0	0.03	0.00	0.00	0.00	0.00	0.00
	11	0.0	0.0	0.04	0.15	0.00	0.00	0.00	0.00
	15	0.0	0.0	0.01	0.01	0.00	0.00	0.00	0.00
	20	0.0	0.0	0.01	0.00	0.01	0.00	0.00	0.00
$(300,120)$	6	0.0	0.0	0.02	0.00	0.00	0.00	0.00	0.00
	7	0.0	0.0	0.01	0.01	0.00	0.00	0.00	0.00
	11	0.0	0.0	0.03	0.12	0.01	0.00	0.00	0.00
	15	0.0	0.0	0.02	0.00	0.00	0.00	0.00	0.00
	20	0.0	0.0	0.00	0.01	0.00	0.00	0.00	0.00
Block									
$(200,80)$	6	0.0	0.0	0.04	0.00	0.00	0.00	0.00	0.00
	7	0.0	0.0	0.00	0.01	0.00	0.00	0.00	0.00
	11	0.0	0.0	0.06	0.21	0.02	0.00	0.00	0.00
	15	0.0	0.0	0.01	0.02	0.00	0.00	0.00	0.00
	20	0.0	0.0	0.03	0.03	0.00	0.00	0.00	0.00
$(300,120)$	6	0.0	0.0	0.05	0.00	0.01	0.00	0.00	0.00
	7	0.0	0.0	0.03	0.01	0.00	0.00	0.00	0.00
	11	0.0	0.0	0.02	0.11	0.00	0.00	0.00	0.00
	15	0.0	0.0	0.03	0.03	0.00	0.00	0.00	0.00
	20	0.0	0.0	0.00	0.00	0.00	0.00	0.00	0.00

Table S6: Type I error for testing $H_0 : \beta_j^{(1)} = \ldots = \beta_j^{(G)} = 0$ vs H_1 : not H_0 at $\alpha = 0.05$, where $n_1, \ldots, n_G$ are set as $n_1 = \ldots = n_G = m$.

Testing	Covariates	j	$\min_g \beta_j^{(g)}$	$\max_g \beta_j^{(g)}$	Methods					
					DFGL	DL	DL-E	DR-B	DL-B	DL-E-B
Homogeneity	AR(1)	1	-0.6	0.6	0.99	0.86	0.19	0.00	0.01	0.00
		2	-0.6	0.6	0.87	0.67	0.08	0.00	0.01	0.00
		3	-0.4	0.4	0.83	0.73	0.19	0.00	0.00	0.00
		4	-0.4	0.4	0.88	0.77	0.11	0.00	0.01	0.00
	Block	1	-0.6	0.6	0.96	0.83	0.25	0.00	0.00	0.00
		2	-0.6	0.6	0.94	0.72	0.11	0.00	0.00	0.00
		3	-0.4	0.4	0.80	0.68	0.09	0.00	0.00	0.00
		4	-0.4	0.4	0.91	0.78	0.25	0.00	0.00	0.00
Significance	AR(1)	1	-0.6	0.6	1.00	1.00	0.60	0.00	0.00	0.00
		2	-0.6	0.6	0.95	0.92	0.34	0.00	0.00	0.00
		3	-0.4	0.4	0.92	0.92	0.25	0.00	0.00	0.00
		4	-0.4	0.4	0.96	0.95	0.33	0.00	0.00	0.00
		5	0.4	0.4	0.74	0.66	0.09	0.00	0.00	0.00
		8	-0.4	-0.4	0.36	0.15	0.02	0.00	0.00	0.00
		9	1.5	1.5	1.00	1.00	1.00	0.03	0.81	0.02
		11	2.5	2.5	1.00	1.00	1.00	0.03	0.87	0.00
		12	2.5	2.5	1.00	1.00	0.00	0.92	1.00	0.82
	Block	1	-0.6	0.6	0.98	0.97	0.60	0.00	0.00	0.00
		2	-0.6	0.6	0.98	0.94	0.37	0.00	0.00	0.00
		3	-0.4	0.4	0.90	0.85	0.12	0.00	0.00	0.00
		4	-0.4	0.4	0.97	0.96	0.41	0.00	0.00	0.00
		5	0.4	0.4	0.86	0.59	0.06	0.00	0.00	0.00
		8	-0.4	-0.4	0.82	0.57	0.09	0.00	0.00	0.00
		9	1.5	1.5	1.00	1.00	1.00	0.15	0.91	0.04
		11	1.5	1.5	1.00	1.00	1.00	0.04	0.92	0.01
		12	2.5	2.5	1.00	1.00	1.00	1.00	1.00	0.93

Table S7: Power at $\alpha = 0.05$ where $n_1 = \ldots = n_4 = 200$, $n_5 = n_6 = n_7 = 300$, and $p = 120$.

Testing	Covariates	j	$\min_g \beta_j^{(g)}$	$\max_g \beta_j^{(g)}$	Methods					
					DFGL	DL	DL-E	DR-B	DL-B	DL-E-B
Homogeneity	AR(1)	5	0.4	0.4	0.02	0.01	0.00	0.00	0.00	0.00
		8	−0.4	−0.4	0.03	0.02	0.01	0.00	0.00	0.00
		9	1.5	1.5	0.07	0.16	0.03	0.00	0.02	0.00
		10	1.5	1.5	0.04	0.19	0.03	0.00	0.01	0.00
		12	2.5	2.5	0.09	0.39	0.08	0.00	0.00	0.00
	Block	5	0.4	0.4	0.05	0.02	0.00	0.00	0.00	0.00
		8	−0.4	−0.4	0.03	0.01	0.01	0.00	0.00	0.00
		9	1.5	1.5	0.08	0.23	0.02	0.00	0.00	0.00
		10	1.5	1.5	0.01	0.20	0.00	0.00	0.00	0.00
		12	2.5	2.5	0.16	0.39	0.07	0.00	0.00	0.00
Significance	AR(1)	6	0.0	0.0	0.02	0.01	0.00	0.00	0.00	0.00
		7	0.0	0.0	0.02	0.01	0.00	0.00	0.00	0.00
		11	0.0	0.0	0.03	0.16	0.00	0.00	0.00	0.00
		15	0.0	0.0	0.02	0.00	0.00	0.00	0.00	0.00
		20	0.0	0.0	0.01	0.02	0.00	0.00	0.00	0.00
	Block	6	0.0	0.0	0.01	0.01	0.00	0.00	0.00	0.00
		7	0.0	0.0	0.03	0.00	0.00	0.00	0.00	0.00
		11	0.0	0.0	0.02	0.16	0.00	0.00	0.00	0.00
		15	0.0	0.0	0.02	0.01	0.00	0.00	0.00	0.00
		20	0.0	0.0	0.01	0.00	0.00	0.00	0.00	0.00

Table S8: Type I error at $\alpha = 0.05$ where $n_1 = \ldots = n_4 = 200$, $n_5 = n_6 = n_7 = 300$, and $p = 120$.

Testing	Covariates	Power			FWER		
		DFGL	DL	DL-E	DFGL	DL	DL-E
Homogeneity	AR(1)	0.402(0.241)	0.160(0.186)	0.005(0.035)	0.020	0.140	0.000
	Block	0.495(0.261)	0.170(0.194)	0.005(0.035)	0.020	0.120	0.000
Significance	AR(1)	0.691(0.116)	0.578(0.101)	0.367(0.058)	0.000	0.030	0.000
	Block	0.723(0.120)	0.551(0.105)	0.370(0.059)	0.000	0.010	0.000

Table S9: Performances of multiple testing at $\alpha = 0.05$, where $n_1 = \ldots = n_4 = 200$, $n_5 = n_6 = n_7 = 300$, and $p = 120$.

References

[1] Wainwright, M.J. *High-Dimensional Statistics: A Non-Asymptotic Viewpoint.* Cambridge Series in Statistical and Probabilistic Mathematics, (2019).

[2] Van de Geer, S., Bühlmann, P., Ritov, Y.A., and Dezeure, R. On asymptotically

optimal confidence regions and tests for high-dimensional models. *Annals of Statistics*, **42**, 1166–1202. (2014).

[3] Raič, M. A multivariate Berry–Esseen theorem with explicit constants. *Bernoulli*, **25**, 2824–2853 (2019).

[4] Zhao, S.D. and Li, Y. Principled sure independence screening for cox models with ultra-high-dimensional covariates. *Journal of Multivariate Analysis*, **105**, 397–411 (2012).

[5] Zheng, Q., Peng, L., and He, X. Globally adaptive quantile regression with ultra-high dimensional data. *Annals of Statistics*, **43**, 2225–2258 (2015).

[6] Li, J., Zheng, Q., Peng, L., and Huang, Z. Survival impact index and ultrahigh-dimensional model-free screening with survival outcomes. *Biometrics*, **72**, 1145–1154 (2016).

[7] Park, S., He, X., and Zhou, S. Dantzig-type penalization for multiple quantile regression with high dimensional covariates. *Statistica Sinica*, **27** (2017).

[8] Negahban, S.N., Ravikumar, P., Wainwright, M.J., and Yu, B. A unified framework for high-dimensional analysis of m -estimators with decomposable regularizers. *Statistical Science*, **27**, 538–557 (2012).

[9] Lee, E.R., Cho, J., and Park, S. Penalized kernel quantile regression for varying coefficient models. *Journal of Statisical Planning and Inference*, **217**, 8–23 (2022).

[10] Park, S., Lee, E.R., and Zhao, H. Low-rank regression models for multiple binary responses and their applications to cancer cell-line encyclopedia data. *Journal of the American Statistical Association*, pages 1–15 (2022).

[11] Javanmard, A. and Montanari, A. Confidence intervals and hypothesis testing for high-dimensional regression. *The Journal of Machine Learning Research*, **15**, 2869–2909. (2014).

[12] Liang, F., Li, Q., and Zhou, L. Bayesian neural networks for selection of drug sensitive genes. *Journal of the American Statistical Association*, **113**, 955–972 (2018).

[13] Li, Q., Shi, R., and Liang, F. Drug sensitivity prediction with high-dimensional mixture regression. *PloS one*, **14**, e0212108 (2019).

[14] Jing, J. et al. Comprehensive predictive biomarker analysis for MEK inhibitor GSK1120212. *Molecular cancer therapeutics*, **11**, 720–729 (2012).

[15] Hayashi, A. et al. Positive regulation of phagocytosis by SIRPβ and its signaling mechanism in macrophages. *Journal of Biological Chemistry*, **279**, 29450–29460 (2004).

[16] Wickham, H. ggplot2. *Wiley interdisciplinary reviews: computational statistics*, **3**, 180–185 (2011).

[17] R Core Team. *R: A Language and Environment for Statistical Computing.* R Foundation for Statistical Computing, (2021).
